# *Ma* Orthologous Genes in *Prunus* spp. Shed Light on a Noteworthy NBS-LRR Cluster Conferring Differential Resistance to Root-Knot Nematodes

**DOI:** 10.3389/fpls.2018.01269

**Published:** 2018-09-11

**Authors:** Cyril Van Ghelder, Daniel Esmenjaud, Caroline Callot, Emeric Dubois, Marianne Mazier, Henri Duval

**Affiliations:** ^1^Institut Sophia Agrobiotech, INRA, CNRS, Université Côte d’Azur, Sophia Antipolis, France; ^2^Centre National de Ressources Génomiques Végétales, INRA, CNRS, Université de Toulouse, Castanet-Tolosan, France; ^3^MGX-Montpellier GenomiX, CNRS, Montpellier, France; ^4^Unité de Génétique et Amélioration des Fruits et Légumes (GAFL), INRA, Montfavet, France

**Keywords:** almond, *Meloidogyne*, nucleotide-binding site - leucine-rich repeat (NBS-LRR), *Prunus*, resistance gene, root-knot nematodes (RKNs), TIR-NBS-LRR (TNL)

## Abstract

Root-knot nematodes (RKNs) are considerable polyphagous pests that severely challenge plants worldwide and especially perennials. The specific genetic resistance of plants mainly relies on the NBS-LRR genes that are pivotal factors for pathogens control. In *Prunus* spp., the *Ma* plum and *RMja* almond genes possess different spectra for resistance to RKNs. While previous works based on the *Ma* gene allowed to clone it and to decipher its peculiar TIR-NBS-LRR (TNL) structure, we only knew that the *RMja* gene mapped on the same chromosome as *Ma*. We carried out a high-resolution mapping using an almond segregating F2 progeny of 1448 seedlings from resistant (R) and susceptible (S) parental accessions, to locate precisely *RMja* on the peach genome, the reference sequence for *Prunus* species. We showed that the *RMja* gene maps in the *Ma* resistance cluster and that the *Ma* ortholog is the best candidate for *RMja*. This co-localization is a crucial step that opens the way to unravel the molecular determinants involved in the resistance to RKNs. Then we sequenced both almond parental NGS genomes and aligned them onto the RKN susceptible reference peach genome. We produced a BAC library of the R parental accession and, from two overlapping BAC clones, we obtained a 336-kb sequence encompassing the *RMja* candidate region. Thus, we could benefit from three *Ma* orthologous regions to investigate their sequence polymorphism, respectively, within plum (complete R spectrum), almond (incomplete R spectrum) and peach (null R spectrum). We showed that the *Ma* TNL cluster has evolved orthologs with a unique conserved structure comprised of five repeated post-LRR (PL) domains, which contain most polymorphism. In addition to support the *Ma* and *RMja* orthologous relationship, our results suggest that the polymorphism contained in the PL sequences might underlie differential resistance interactions with RKNs and an original immune mechanism in woody perennials. Besides, our study illustrates how PL exon duplications and losses shape TNL structure and give rise to atypical PL domain repeats of yet unknown role.

## Introduction

Plant-parasitic nematodes cause huge losses on agriculture worldwide (Nicol et al., 2011). Based on their economic impact, the root-knot nematodes (RKNs) *Meloidogyne* spp. are considered as the number one threat caused by nematodes on crops (Jones et al., 2013). The predominant RKN species are the tropical parthenogenetic mitotic species *Meloidogyne arenaria*, *M. incognita* and *M. javanica* and to a lesser extent the temperate parthenogenetic meiotic *M. hapla*. Nevertheless, minor RKN species such as *M. enterolobii, M. chitwoodi*, or *M. fallax* have been recently described as emerging problems for agriculture possibly promoted by climate change or transition in agronomic production (Elling, 2013). The extreme polyphagy of RKNs, characterized by their ability to infect over 5500 plant species, highly increased the interest to identify the actors and generic mechanisms underlying their interaction with plants, especially those involved in plant immunity (Blok et al., 2008).

The plant innate immunity relies on two main processes. The first level involves transmembrane receptors that detect generic molecules usually associated with pathogens. Following this non-specific recognition, this pathway, called pathogen-associated molecular patterns (PAMP) triggered immunity (PTI), activates MAP-kinases cascades leading to plant immunity. This first level of defense can be targeted at different levels by particular pathogen-secreted molecules, known as effectors, leading to inhibition of immunity. In return, effectors can be recognized in a specific manner through a second mechanism so called effector-triggered immunity (ETI) (Jones and Dangl, 2006). The cornerstone of ETI is the NBS-LRR gene family that is involved in plant adaptation to biotic and abiotic stresses. Hundreds of NBS-LRR genes are usually found in genomes of flowering plants (Meyers et al., 1999). They can be subject to deletion through unequal crossing-over or (non)-homologous repair events (Luo et al., 2012) or to expansion involving tandem, ectopic duplications and, to a lesser extent, segmental duplications (Leister, 2004). The dynamic mechanism of gene duplications explained the generic cluster organization of NBS-LRR genes found in plant genomes. This large gene family is split formally into two sub-groups, the TIR-NBS-LRR (TNL) and non-TIR NBS-LRR according to the presence or not of a Toll-interleukin-1 receptor domain in the N-terminal part of the protein (Meyers et al., 1999). Given their modular organization and distinctive domain functions, TNLs act as a multi-function tool for plant immunity. Indeed, the leucine-rich repeat (LRR) domain is polymorphic and often described by its ability to detect pathogens through direct or indirect effector recognition (Jones and Dangl, 2006). The latter process gives rise to some conceptual examples illustrated by the guard protein and decoy models (van der Hoorn and Kamoun, 2008). The central nucleotide-binding site (NBS) domain binds alternatively ADP or ATP leading to conformational modification and by consequence to switch from “OFF” to “ON” states of the protein (Bernoux et al., 2016). The transition between the LRR and the NBS domains is carried by a conserved exon coding for the NBS-LRR Linker (NLL). Once the protein is activated, the TIR domain is released triggering a downstream signaling pathway leading to plant immunity that is often associated with a programmed cell death called Hypersensitive-like Response (Heath, 2000). However, each domain presumably covers a larger field of competence such as intra or extra-molecular interactions involved in the maintenance of the 3D protein folding, dimerization or alternative effector recognition (Rairdan and Moffett, 2006; Burch-Smith et al., 2007). Besides these three canonical domains, it exists a forth domain, peculiar to TNLs, named post-LRR (PL) domain after its regular position (Van Ghelder and Esmenjaud, 2016). This domain, with unknown role or structure, is usually present in one copy in most of the cloned TNL genes such as *N*, *RPS4*, or *Gro1-4* (Whitham et al., 1994; Gassmann et al., 1999; Paal et al., 2004). Over the last decades, many efforts have been deployed to identify novel genes conferring strong and sustainable natural resistance in plants with agronomical interest. The *Prunus* species are important fruit crops worldwide and, amongst them, *Prunus persica* (peach), *P. cerasifera* (Myrobalan plum) and *P. dulcis* (almond), *qua* perennials, are challenged continuously by RKNs. The common ancestor of the *Prunus* genus emerged approximately 61 Myr in Eastern. The plums (subgenus *Prunophora*) originating from the Eastern Europe and the Middle East, diverged earlier than the almond and peach (subgenus *Amygdalus*) that are native from Western and Eastern Asia (estimated absolute age of 54.6 Myr for *Prunophora* versus 48.9 Myr for *Amygdalus*) (Chin et al., 2014). Although the almond genome is not available yet, the compact peach genome (265 Mbp; Verde et al., 2013) can be considered as the reference genome for *Prunus* species in particular for the diploid (2n = 16) species, peach, almond, Japanese and Myrobalan plums, and apricot.

The *Ma* gene, from *P. cerasifera*, was the first TNL gene, conferring resistance to RKNs, cloned in a woody perennial plant (Claverie et al., 2011). Conversely to the *Mi-1* tomato CNL R gene (Milligan et al., 1998), the *Ma* gene confers a heat-stable and complete-spectrum resistance to RKNs (Claverie et al., 2011), and no natural or selected virulent isolates have been detected yet (Khallouk et al., 2013). Besides these attractive biological properties, this gene displays a unique C-terminal structure, which is only found in the *Prunus* and *Malus* genera, made of five PL domains completing the TIR-NBS-LRR canonical part (Van Ghelder and Esmenjaud, 2016). Apart from the *Ma* gene, lying on *Prunus* chromosome 7, several other RKNs *R* genes with more restricted resistance spectra have been identified (Saucet et al., 2016). These are the *RMia* gene from peach, which is most probably a TNL located in a 92-kb interval on the chromosome 2 (Duval et al., 2014) and the *RMja* gene from almond. Interestingly, the latter confers a resistance to *M. javanica* but not to *M. incognita*. A low-resolution mapping strategy carried out on few progenies localized the *RMja* gene on the chromosome 7 (linkage group 7) (Van Ghelder et al., 2010).

The overall goal of this study was to pave the way to identify the molecular determinants involved in the RKN resistance. We have considered three *Prunus* species through accessions that display distinct spectra for resistance to RKNs ranging from complete (plum P.2175/*Ma*) to partial (almond Alnem1/*RMja*) and null (peach Lovell/*Prupe.7G065400*). The specific research objectives were: 1) to localize the *RMja* gene in almond using high-resolution mapping; 2) to obtain a reliable sequence of the *RMja*-candidate region; 3) to identify the polymorphism that could be involved in the resistance mechanism; 4) and to investigate the evolution of the complete *Ma*/*RMja* TNL cluster. We showed that the *RMja* gene is located in the *Ma* TNL cluster. Almond NGS genomes, BAC sequences and expression data strongly suggest that the *RMja* gene is the ortholog of the *Ma* plum gene. Additionally, we shed light on the dynamic evolution of the complete *Ma* TNL cluster in plum, peach and almond revealing an atypical focal point for RKN resistance in *Prunus* spp.

## Materials and Methods

### Plant and Nematode Materials

The resistant (R) accession *P. dulcis* cv. ‘Alnem1’ controls *M. arenaria* and *M. javanica* but not *M. incognita* and a single gene named *RMja* has been identified to confer resistance against *M. javanica* (Kochba and Spiegel-Roy, 1975; Esmenjaud et al., 2009; Van Ghelder et al., 2010). We produced an intraspecific progeny from almond. Accession ‘Alnem1,’ carrying the homozygous dominant *RMja* gene (*RMja/RMja*), was crossed with the susceptible (S) accession ‘Lauranne’ (recessive for *RMja*; *rmja1/rmja2*) to create a F1 heterozygous progeny. Several Lauranne x Alnem1 F1 hybrids were self-pollinated in greenhouses to produce a segregating F2 progeny (F2 LxA) with three different resistance genotypes (*RMja/RMja, RMja/rmja1or2*, and *rmja1or2/rmja1or2*). Shelled almonds were collected and placed onto a culture medium to induce germination.

The population *M. javanica* “Higuera” from Cabrils (Cataluna, Spain) was reared from a single egg mass and maintained on RKN susceptible tomatoes (*Lycopersicon esculentum* cv. ‘St. Pierre’). Its RKN species identity was verified using isoesterase profiling method as defined by Janati et al. (1982).

### Experimental Procedure for Phenotyping

A number of 1448 F2 LxA seedlings, 6-month-old, were planted in pairs in 5-liter pots, placed in greenhouse, irrigated individually every 2 days and grown until harvested for rating at a mean temperature of 25°C (extremes 20–30°C). In parallel, 5-leaf tomato plantlets grown in 250-ml containers were inoculated with 500 24–72-h old juveniles of *M. javanica*. After 2 months, aerial parts of tomato plants were removed and the contaminated soil of one container, including the infected root system, was transplanted into each Prunus pot to induce a high- and durable-inoculum pressure. After 6 months, the root system of each Prunus plant was carefully examined and rated into two classes: resistant (R) (absence of galls) or susceptible (S) (presence of gall(s)).

### Nucleic Acid Extraction, cDNA Synthesis and Genotyping

The genomic DNA of all F2 plants was extracted using 100 mg of frozen young leaves. Each sample was ground with a mixer mill in 330 μl of extraction buffer (sorbitol 0,35M, Tris 0,1M, EDTA 5 mM, 4 mg of sodium metabisulfite), 330 μl of lysis buffer (Tris 0.2 M, EDTA 50 mM, NaCl 2 M, CTAB 2%) and 130 μl of sarkosyl 5%. After a chloroform-isoamylalcohol (24:1) procedure, a precipitation with isopropanol and three ethanol washes, the DNA content was eluted in water and treated with RNAse.

RNA was extracted using 100 mg of young roots or leaves according to the procedure of Tong et al. (2012) with modifications. Fresh RNA samples were treated with DNAse using the Turbo DNA-free kit and evaluated with a Nanodrop. First-strand cDNA were synthesized using the maxima H minus first strand synthesis kit with either specific and oligo (dT) primers. 3 μl of first-strand cDNA were used in PCR using primers defined in **Supplementary Table S1** to conduct transcript detection. The *P. persica* actin gene Prupe.02G235000 was used as control for cDNA synthesis.

PCR were carried out using the MyTaq DNA polymerase kit or the Expand long range kit for longer fragments following the manufacturer’s instructions. SSR primers used (**Supplementary Table S1**) for the genotyping were 5′-fluorescent-labeled with different fluorophores and PCR products were analyzed with a 3130xl Genetic Analyzer.

### Genome Sequencing of the Almond Parents

DNA extracted from leaves of accessions ‘Alnem1’ and ‘Lauranne’ was used to prepare genomic libraries. Tagmentation and PCR amplifications were carried out using the Illumina Nextera DNA sample preparation kit following the supplier’s instructions. The validation of the genomics libraries were performed with a DNA quantification using an Agilent high sensitivity chip and by qPCR. The cluster was carried out inside a flow-cell using the Illumina cluster generation kit. Then, a paired-end 125 sequencing was achieved using an Illumina Hiseq 2500 with a sequence by synthesis (SBS) technique.

Image analysis and base calling were performed using the HiSeq Control Software and Real-Time Analysis component. Demultiplexing was performed using Illumina’s Conversion Software (bcl2fastq 2.17). The quality of the data was assessed using FastQC from the Babraham Institute and the Illumina software SAV (Sequence Analysis Viewer). Potential contaminants were investigated with the FastQ Screen software from the Babraham Institute.

BWA-MEM (v 0.7.12) was used to align reads to the *Prunus persica* genome (v 2.0). MGX-Montpellier GenomiX core facility performed the sequencing of both clones. The raw data of both genomes have been deposited with links to the NCBI BioProject accession number PRJNA448729 (BioSamples numbers SAMN08863554 and SAMN08863555).

### Genomic Library and Bacterial Artificial Chromosome (BAC) Sequencing

High molecular weight (HMW) genomic DNA was prepared from young frozen leaves from accession ‘Alnem1’ as described by Peterson et al. (2000) and Gonthier et al. (2010). Agarose embedded HMW DNA was partially digested with HindIII, subjected to two size selection steps by pulsed-field electrophoresis using a CHEF Mapper system. DNA was eluted, ligated into the pIndigoBAC-5 HindIII-Cloning Ready vector and transformed in Escherichia coli electrocompetent cells. Pulsed-field migration programs electrophoresis buffer and ligation desalting conditions were performed based on Chalhoub et al. (2004). The resulting library represents ∼7-fold coverage of accession ‘Alnem1’ (48 plates, 18,432 BAC clones and mean insert size of 134 kb). BAC clones were spotted on nylon membrane, screened with radioactive labeled probes designed in exons PL1, PL2 and PL5 and revealed by High-density filter reader program. The positive clones were verified by real-time PCR using the specific primers designed in exons PL1, PL2 and PL5. 2 μg of each individual BAC clone of interest were pooled for the construction of a SMRT^®^ library using the standard Pacific Biosciences preparation protocol for 10 kb library with PacBio^®^ Barcoded Adapters. The pool was then sequenced in one SMRT cell using the P6 polymerase with C4 chemistry. Sequencing was performed on PacBio RS II sequencer. After a demultiplexing step, the sequence assembly was performed following the HGAP PacBio workflow (Chin et al., 2013), and using the SMRT^®^ Analysis (v2.3) software suite for HGAP implementation^1^. BAC ends sequences confirmed the position of selected clones, on the peach genome. BAC library construction and screening, and BAC clone sequencing and assembly were performed by the INRA-CNRGV. The data have been deposited with links to NCBI BioProject accession number PRJNA448736.

### Sequence Analysis

HMM-based gene structure predictions were carried out using FGENESH (Solovyev et al., 2006) and the *P. persica* specific gene-finding parameters. Read alignment and index files (BAM and BAI files), genomic feature files (GFF3) and *P. persica* v2.0.a1 genome^2^ were analyzed with the Integrative Genomics Viewer (IGV) (Robinson et al., 2011). Alignments, sequence trimmings, mutation rate and phylogenetic analyses were evaluated using MEGA7 (Kumar et al., 2016). Conservative, semi- and non-conservative amino acid substitutions were determined using the Clustal Omega assignment (Sievers et al., 2011). Detection of promoter and regulatory elements were carried out using Cister (Frith et al., 2001) with the *Ma*, *RMjaGC2* and *Prupe.7G065400* genes extended with 7101 bp upstream each of them. We screened 5414, 5414, and 2721 bp upstream the *Ma*, *RMjaGC2* and *Prupe.7G065400* genes, respectively, for W-boxes using PLACE 26.0 (Higo et al., 1999) and PlantTFDB 4.0 (Jin et al., 2017).

## Results

### High-Resolution Mapping of the *RMja* Gene

**FIGURE 1:**
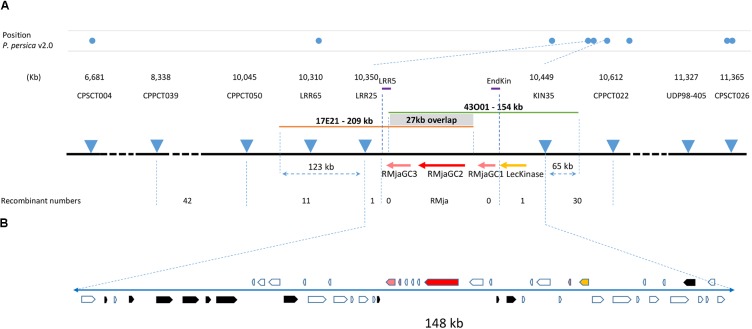
High-resolution mapping of the *RMja* region. **(A)** Molecular markers used in this study and their position in the peach genome v2.0 are indicated at scale with blue dots. The numbers of recombinants are indicated below the respective markers and the positions of the two BACs, 17E21 and 43O01, are indicated with orange and green lines, respectively. **(B)** Magnification of the final 148-kb interval in almond containing the *RMja* gene. The *RMjaGC2* gene (*Ma* ortholog) is highlighted in red, the *RMjaGC1* and *RMjaGC3* genes in pink, the lectin tyrosine kinase in yellow and transposable elements are indicated in black. The white arrows indicate predicted peptides in the interval that are described in **Supplementary Table S3**.

We localized the *RMja* gene between two SSR markers that cover a window of 0.14 cM. This region corresponds to a physical interval of 99 kb in the peach genome v2.0.a1. The high-resolution mapping strategy conducted on 1448 total F2 almond seedlings revealed a single recombination event for each of the flanking markers, LRR25 and KIN35. Internal primers named LRR5 and EndKin designed within this final interval, amplified fragments that eliminated any recombination events allowing us to refine the *RMja* gene localization (**Figure 1A**). This mapping was actually performed in two steps. In a first step, we used a progeny composed of 907 F2 seedlings that clearly split into 625 R individuals and 282 S individuals. We tested the S and R parental accessions together with twenty hybrids to select reliable and polymorphic 5′-fluorescent-labeled SSR markers spread in this LG7 region from the peach genome (Verde et al., 2013). Genotyping the 907 progenies with the flanking markers CPPCT039 and CPPCT022 (**Supplementary Table S1**) revealed 27 and 16 recombinant individuals on each side of the gene, respectively. In a second step, the 541 F2 individuals obtained segregated into 411 R: 130 S individuals (3 R: 1S ratio; χ^2^ = 0.27; *P* = 0.6). The overall individuals segregated into 1036 R: 412 S individuals (χ^2^ = 9.2; *P* = 0.002). In the candidate interval, we developed three new SSR markers, LRR65, LRR25 and Kin35 (**Supplementary Table S1**) that revealed eleven, one and one recombinant individuals in the total progeny, respectively (**Figure 1A**).

### The *RMja* Gene Is Located in a Region Containing the *Ma* Ortholog

**FIGURE 2:**
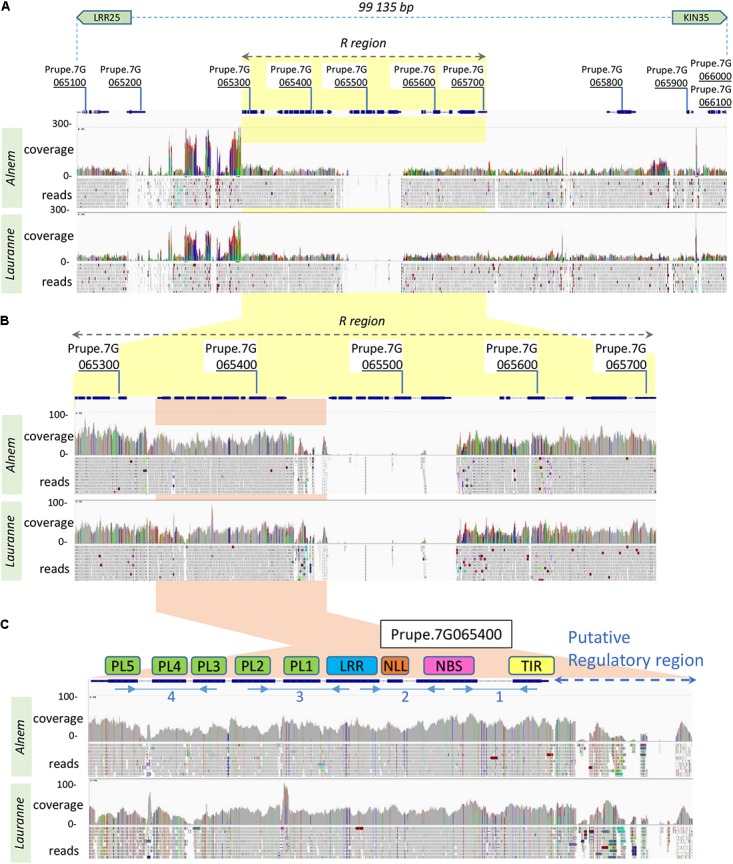
Mapping of the R and S almond reads onto the peach genome v2.0 at the *RMja* candidate region. **(A)** Region covering the interval between the Kin35 and LRR25 markers including the eleven predicted genes in the peach genome. **(B)** Magnification of the R region containing four TNL genes (Prupe.7G065300 to Prupe.7G065600) and the tyrosine kinase (Prupe.7G065700) with an adapted coverage scale. **(C)** Enlargement of the *Prupe.7G065400* gene (*Ma*/*RMjaGC2* ortholog) with its putative regulatory region. Localizations of the four fragments, chosen to demonstrate the *RMjaGC2* transcription, are indicated with blue arrows under the predicted exons. Reads coverage and mapping are indicated in gray or color bars for homologous or polymorphic nucleotides with the peach sequence, respectively.

In the peach genome, a further examination of the *RMja* final interval marked out by the LRR25 and KIN35 markers, revealed eleven candidate genes named *Prupe.7G065100* to *Prupe.7G066100* (**Supplementary Table S2**). Among these candidate genes, a functional annotation identified one tyrosine kinase (*Prupe.7G065700*) and four TNLs (*Prupe.7G065300* to *Prupe.7G065600*) including the *Ma* ortholog (*Prupe.7G065400*). We sequenced the genome of the parental accessions ‘Alnem1’ (R) and ‘Lauranne’ (S) to further characterize the candidate region in almond. The phyletic proximity between peach and almond allowed us to map accurately the reads on the *P. persica* genome assembly (v2.0.a1). All over the region, the alignment of both the R and S almond reads onto the *P. persica* genome indicated a similar pattern involving alternation between syntenic regions with peach, and indels of unknown size (**Figure 2A**). We identified two profiles in the syntenic regions showing either (i) a standard coverage (20× – 50×) and moderate nucleotide polymorphism or (ii) a high coverage (>100×) and nucleotide polymorphism. The first pattern corresponds to orthologous loci whereas the second, presumably, reflects the misalignment of ectopic transposable elements (TEs). Magnification of the region spanning from the first TNL (*Prupe.7G065300*) to the lectin kinase (*Prupe.7G065700*), arbitrarily named R region, highlights the absence of the ortholog of the TNL *Prupe.7G065500* in almond (**Figure 2B**). The almond orthologs of *Prupe.7G065700* (lectin kinase) and *Prupe.7G065400* (*Ma* ortholog TNL) display a significant level of homology (illustrated in gray in **Figure 2B**) unlike the orthologs of *Prupe.7G065300* and *Prupe.7G065600* showing nucleotide polymorphism and numerous indels. More specifically, the *Prupe.7G065400* gene shows conserved structural features and low polymorphism in predicted coding regions in either the homozygote ‘Alnem1’ (R), or ‘Lauranne’ (S), or peach ‘Lovell’ accessions. Only the upstream putative regulatory region displays substantial divergences between both almonds and the peach accessions (**Figure 2C**).

### BAC Sequences and Expression Data Strongly Suggest That *RMja* Is the Ortholog of the *Ma* Gene

We selected BAC clones that correspond to the region of interest from a genomic library constructed from the R accession ‘Alnem1.’ We sequenced two overlapping BAC clones (‘17E21’ and ‘43O01’) [PRJNA448736] covering the KIN35-LRR25 interval, using long-read sequencing technology to overcome potential misassembly due to repeated sequences. These two sequences meet the quality criteria with a quality value QV > 48 meaning a high confidence in nucleotide identification. The BAC clone ‘43O01’ contains an insert of 154 kb that can be aligned from 65 kb upstream the KIN35 marker to the *RMjaGC3* gene. The second BAC clone sequence ‘17E21’ is 209 kb long and maps from the *RMjaGC1*- *RMjaGC2* intergenic region to 123 kb downstream the LRR25 marker (**Figure 1A**). The overlapping region between the two BAC clones covers a sequence of 27 kb that displays a similarity of 99.97 %. Only eight nucleotide deletions were found and localized in five SSR regions in the 27 kb, whereas the remainder 190 kb identified SSRs were strictly identical. The fragment length between the KIN35 and LRR25 markers is 49 kb longer in almond (**Supplementary Table S5**) than in peach.

Gene finding analyses and functional annotations revealed 48 predicted ORFs including one lectin kinase, only three TNLs of different sizes, small peptides (potential artefacts) and TEs that explain the size variation in this region (**Figure 1B** and **Supplementary Table S3**). The lectin kinase that precedes the TNL cluster is highly conserved between plum, peach and almond (>98% amino acid identity). The protein is composed of a signal peptide, an extra-cellular carbohydrate-binding domain, a transmembrane portion and an intra-cellular tyrosine kinase domain (**Supplementary Table S4**). This gene is the ortholog (54.8% identity) of the *Arabidopsis thaliana* gene coding protein *At2g41890* assigned to the GO biological process of pollen recognition and self-incompatibility. A blast analysis revealed that the *Ma* and lectin kinase loci existed separately in Rosales species but only co-localized in *Prunoideae* species as there are only found physically close to each other in *Malus*, *Pyrus* and *Prunus* genomes (**Supplementary Table S6**).

Immediately after the kinase, we found a TIR domain-only protein (*RMjaGC1*). The analysis of the immediate downstream sequence revealed a disrupted NBS domain followed by parts of retrotransposons suggesting a progressive gene loss. Except the *RMjaGC2* gene, the candidate interval then only contains a last TNL, *RMjaGC3*. This gene is predicted to be split into a TIR domain-only protein followed by NBS-LRR protein in which, the LRR domain is truncated (232 aa) due to a frameshift mutation inducing a premature stop codon. Furthermore, a retrotransposon fragment, present on the minus strand, disrupts its putative C-terminal part (**Figure 1B**).

**FIGURE 3:**
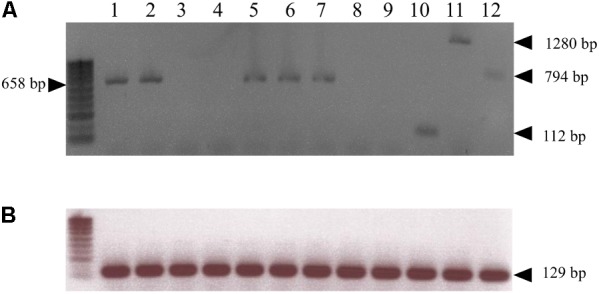
Transcript detection of *Ma* homologous genes. **(A)** cDNA amplifications for the *Ma* orthologous genes from ‘Alnem1’ (*RMjaGC2*) (lanes 1 and 2, from leaves and roots, respectively), ‘Lauranne’ (S) (lanes 3 and 4, from leaves and roots, respectively), the ‘R recombinant’ (between markers KIN35 and EndKin) (lane 5 from leaves), ‘P.2175’ (*Ma*) (lane 6 from leaves), and ‘Lovell’ (*Prupe.7G065400*) (lane 7 from leaves) using primers ORF5F/LRRF1_RC amplifying the fragment 2 (658 bp) shown in **Figure 2C**. The corresponding genomic fragment size of *RMjaGC2* is 1042 bp. cDNA amplifications targeting *RMjaGC1* (lane 8) and *RMjaGC3* (lane 9) sequences in ‘Alnem1’ (leaves) (using the specific primers TIRRMjaGC1F/R, TIRRMjaGC3F/NBSRMjaGC3R, respectively) failed to amplify any fragments. Amplifications of the fragments 1 (TIRRMjaGC2F/NBSRMjaGC2R; 112 bp), 3 (primers EPISLRRF/ PL2F2_RC; 1280 bp) and 4 (primers PL3F/PL5R3; 794 bp) from *RMjaGC2* transcript in ‘Alnem1,’ shown in **Figure 2C** (lanes 10, 11 and 12, respectively). **(B)** cDNA amplifications of the actin gene (Prupe.2G235000; 129 bp) used as control.

The *RMjaGC2* gene is the sole TNL in the R candidate-region to display a complete structure with full-length domains and to be constitutively transcribed. Indeed, we carried out transcript analysis in the ‘Alnem1’ R and ‘Lauranne’ S parental accessions and in several R F2 individuals. In Alnem1 as in R F2 individuals, we successfully amplified and sequenced four fragments from the *RMjaGC2* transcript, using primers designed across exons (**Supplementary Table S1** and **Figure 2C**) and cDNA from leaves and roots as illustrated in **Figure 3**. We also detected the *RMjaGC2* transcript in the single recombinant between KIN35 and EndKin markers, which was phenotypically resistant. Unlike R accessions, the same manipulation failed to detect any *RMjaGC2* transcripts in ‘Lauranne’ (S). In parallel, we failed to amplify any transcripts of *RMjaGC1* and *RMjaGC3* genes in ‘Alnem1.’ A similar approach detected the transcripts of the *Ma* and *Prupe.7G065400* genes in leaves of RKN-free plants of each of the accessions *P. cerasifera* cv. ‘P.2175’(the *Ma* donor) and *P. persica* cv. ‘Lovell,’ respectively, suggesting a constitutive expression and an absence of tissue-specificity in the three genes (**Figure 3**). Moreover, a blast analysis using the *Ma*, *RMjaGC2* and *Prupe.7G065400* transcript sequences as queries against RNAseq and ESTs data from diverse studies, confirmed the transcription of this gene in (i) roots and leaves of *P. persica* and (ii) in various cultivars from different *Prunus* species (**Supplementary Table S6**).

An *in silico* analysis predicted a regulatory region approximately 1kb upstream the start codon of the *RMjaGC2* gene while no consistent *cis*-element binding sites were detected in the vicinity of the *RMjaGC1* and *RMjaGC3* genes. The predicted *RMjaGC2* regulatory region appeared to be very similar to that of peach but quite different from that of plum where multiple regulatory regions are detected (**Supplementary Figure S1**). In parallel, we screened the upstream region of the *Ma*, *RMjaGC2* and *Prupe.7G65400* genes for putative W-boxes (WRKY binding site). Two independent predictions identified an enrichment (>40%) of putative W-boxes localized upstream the *Ma* and *RMjaGC2* genes compared to the single one present upstream the *Prupe.7G65400* (**Supplementary Figure S1**). In peach, an insertion of a TNL gene (*Prupe.7G65500*) has probably replaced a part of the W-boxes in this region.

### The PL Domains Contain Substantial Variations Between *RMjaGC2* and Its Orthologs

To date, the *Ma* gene is the sole gene conferring a full resistance to all RKN species tested including *M. incognita*, *M. arenaria* and *M. javanica*. It belongs to a cluster of three TNL genes (of which two display structural defects) preceded by a tyrosine kinase. The three orthologous genes (*Ma*, *RMjaGC2*, *Prupe.7G65400*) are highly similar (>96% CDS identity). While the *Ma* coding sequence displays a similar identity with peach and almond, its predicted protein shows a slightly higher identity to *RMjaGC2* (94.41%) than to *Prupe.7G065400* (94.12%) (**Table 1**).

**Table 1:** Percent identity matrix between the three orthologous genes, CDS and proteins, *Ma*, *Prupe.7G065400* and *RMjaGC2*.

		*Prupe.7G065400*	*Ma*
Gene UTR incl.	*RMjaGC2*	98.42	91.41
	*Prupe.7G065400*		91.52
CDS	*RMjaGC2*	99.20	96.65
	*Prupe.7G065400*		96.67
Protein	RMjaGC2	98.78	94.41
	Prupe.7G065400		94.12

**FIGURE 4:**
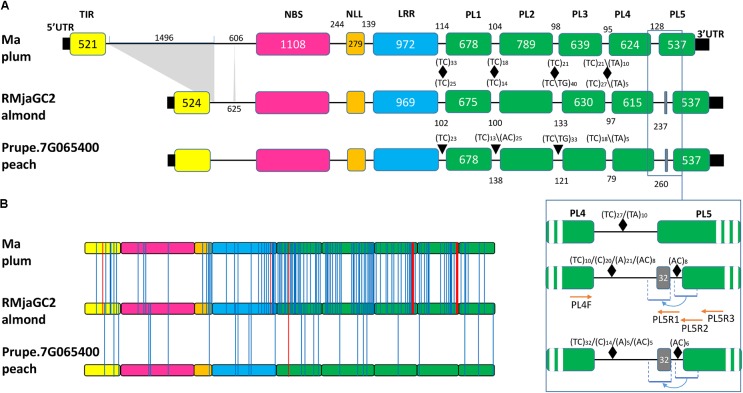
Structural and sequence comparison between the three orthologous genes and proteins, *Ma*, *RMjaGC2* and Prupe.7G065400. **(A)** Intron-exon structures of the three orthologous genes. Only the differential sizes of introns and exons are indicated in base pairs. SSRs are indicated with black triangles together with repeat numbers. The blue frame shows the enlargement of the PL4-PL5 junction that highlights a partial duplication of the last exon and the primer localizations (red arrows) used in the study. **(B)** Localization of the amino acid substitutions (blue lines) between the RMjaGC2 predicted protein and its two orthologs. Indels are illustrated with red lines. The TIR, NBS, NLL, LRR and PL domains are shown in yellow, pink, orange blue and green, respectively.

A gene structure analysis revealed a disparity localized in the first and last intronic regions of these genes. In *Ma*, the first intron between the TIR and NBS-coding exons displays an approximate 1.5 kb fragment that is absent in both *RMjaGC2* and *Prupe.7G065400* (**Figure 4A**). Each PL-coding exon is preceded by dinucleotide SSRs implying polymorphic areas. The final intron displays a differential pattern. Indeed, we noticed that in the *RMjaGC2* and *Prupe.7G065400* genes, the final intron exhibits a duplication of a fragment containing a part of the intron and 32 nucleotides of the last exon including the splicing acceptor site (**Figure 4A**). This singularity might induce alternative splicing and/or truncated mRNA. Using selective primers, PCR experiments carried out with Alnem1 cDNA, failed to amplify any alternative transcripts and only the predicted PL4-PL5 was detected (**Figure 4A**). Nevertheless, we cannot exclude that alternative forms might exist in low numbers or might be induced under specific conditions.

At the peptide level, we identified 110 amino acid mutations that are specific to Ma, 12 that are specific to RMjaGC2 and 14 that are only present in Prupe.7G065400. Unlike the polymorphism detected between RMjaGC2 and Prupe.7G065400, the mutations between RMjaGC2 and Ma are unevenly distributed. Indeed, the variation are mostly assigned to specific domains or parts of domains such as the C-terminal parts of the NLL, LRR, PL2, PL3 and PL4 domains. The highest numbers of mutations are present in PL1 and PL2 and indels in PL3 and PL4 domains (**Figure 4B**).

We established a repertoire of mutations that are (1) only present in Ma (Ma specific) compared to both RMjaGC2 and Prupe.7G065400, (2) specific to RMjaGC2 (RMjaGC2 specific) or (3) common to both Ma and RMjaGC2 and so differing from Prupe.7G065400 (Ma-RMjaGC2 specific). This latter category might highlight crucial amino acid for resistance whereas the first two categories might be associated to spectrum determinants of the resistance. Each mutation is assigned to conservative, semi-conservative or non-conservative mutation subsets as such characteristics often have a differential impact on protein functioning (**Table 2**). In Ma-RMjaGC2 specific mutations, we detected two non-conservative events in both PL1 (T1103R; G1150R) and PL2 (P1408Q; T1423R) domains that may influence the resistance mechanism. The non-conservative mutations that are specific to Ma are mostly localized in the LRR, PL1, PL2, PL3 and PL4 domains with a maximum of 12 non-conservative amino acid mutations in the PL2. RMjaGC2 displays an alanine deletion in the PL1 and one non-conservative mutation in PL2, PL3 and PL4 (S1281L, Q1570P and T1668L, respectively) that may be in relation to its selective spectrum of resistance.

**Table 2:** Distribution of the conservative (C), semi-conservative (S) and non-conservative (N) amino acid mutations over the different domains.

	Total			TIR			NBS			NLL			LRR			PL1			PL2			PL3			PL4			PL5		
Mutations	C	S	N	C	S	N	C	S	N	C	S	N	C	S	N	C	S	N	C	S	N	C	S	N	C	S	N	C	S	N
Ma specific	53	13	44	3	0	3	3	0	0	4	1	0	5	1	6	8	7	7	12	1	12	8	2	7	6	1	9	4	0	1
RMjaGC2 specific	2	5	5	0	0	1	1	0	0	0	1	0	1	2	0	0	1	1	0	0	1	0	0	1	0	0	1	0	1	0
RMjaGC2 & Ma specific	8	2	4	2	0	0	1	1	0	0	0	0	2	0	0	1	0	2	0	1	2	0	0	0	0	0	0	2	0	0

**FIGURE 5:**
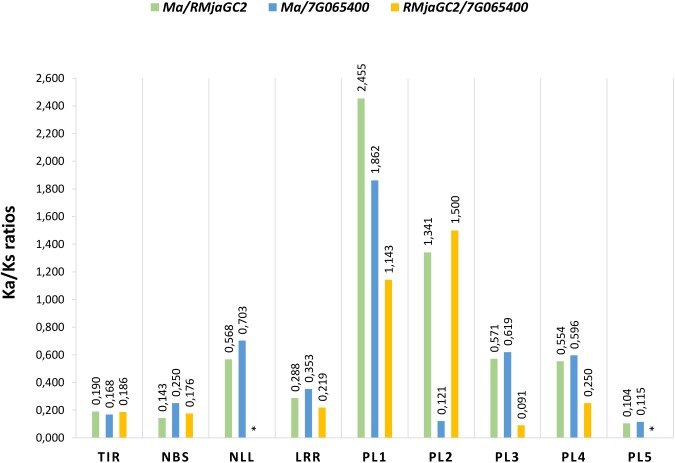
Non-synonymous (Ka) to synonymous (Ks) substitution ratios (Ka/Ks) in the different domains between the three orthologs *Ma*, *RMjaGC2* and Prupe.7G065400. ^∗^Indicates absence of Ka/Ks ratio due to a null Ks.

The non-synonymous (Ka) to synonymous (Ks) substitutions ratio measured on the entire CDS (0.413, 0.368, 0.278 for *Ma/ Prupe.7G065400, Ma/RMjaGC2* and *RMjaGC2/Prupe7G.065400*, respectively) suggest that a negative selection occurs. Nevertheless, an exon-based Ka/Ks ratio analysis revealed that the different domains show highly variable ratios (**Figure 5**). Indeed, the Ka/Ks ratios of the TIR, NBS and PL5 domains are <<1 suggesting that these domains are under strong negative selection. Conversely, the PL1 and PL2 domains display Ka/Ks ratios >>1 suggesting that adaptive evolution occurs in this region. The PL2 domain is atypical as the measured ratios are extremely different between the genes considered, thus preventing any definitive conclusion related to it. The remaining domains (NLL, LRR, PL3 and PL4) display variable Ka/Ks ratios, mainly between 0.2 and 0.7 (**Figure 5**).

### *Ma* Cluster Evolution

**FIGURE 6:**
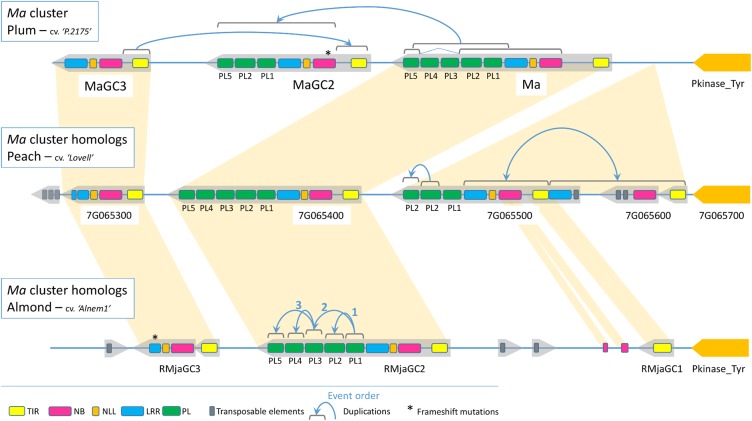
Schematic representation of the R region containing the *Ma* TNL cluster. Structural composition and rearrangements occurring in the ten TNL homologous genes in peach, plum and almond.

The generation of the almond sequence, together with its syntenic regions in plum and in peach, offered us the opportunity to study the evolution of a cluster containing atypical and functional TNL genes. The pairwise analysis of the ten different TNL genes detected in the cluster revealed two conserved loci over the three species (**Figure 6**). The first conserved locus includes the *MaGC3, Prupe.7G065300* and *RMjaGC3* genes that display similar structure with intact TIR, NBS and NLL domains but a truncated LRR domain and no PL domains due to TEs insertion or frameshift mutations inducing premature stop codons. The second conserved locus includes the *Ma*, *RMjaGC2* and *Prupe.7G065400* genes, characterized by a complex structure. The *Prupe.7G065500, Prupe.7G065600* and *RMjaGC1* genes are related to each other and underwent severe reorganizations including TEs insertions or duplications leading to chimeric structures. They emerged either from the *Prunus* ancestor and have been lost in plum, or they are posterior to the split between plums and *Amygdalus*.

In plum, pairwise and phylogenetic analyses of the diverse domains revealed that the *MaGC2* gene recently emerged from partial duplications of the *Ma* and *MaGC3* genes with internal rearrangements. Indeed, the reorganization involves a TIR domain originating from *MaGC3*, and a remaining sequence originating from the *Ma* gene except two lost exons (PL3 and PL4). In *RMjaGC2* and its orthologs, the PL1 domain is the closest PL domain to the outgroup (the PL domain of the tobacco *N* gene). It probably duplicated to give birth to PL2, which then diverged. PL3, PL4 and PL5 domains display a significant identity between each other and are closer to PL1 than the PL2 domains (**Figure 6** and **Supplementary Figure S2**). These results suggest that the PL1 domain underwent exon duplication to generate (i) PL2, a long time ago, and (ii) one of PL3, PL4 or PL5, which rapidly duplicated itself to produce this unique structure.

## Discussion

### The *RMja* Gene Is Presumably the *Ma* Ortholog

Our high-resolution mapping together with the peach genome and *Ma* gene resources helped us to precisely locate the *RMja* gene. This approach, based on a large progeny, points out a limited window in which very few predicted ORFs are credible candidate genes to confer such a resistance. In ‘Alnem1,’ the precise annotation of the *RMjaGC2* gene and its environment together with expression data strongly suggest that the *RMjaGC2* gene is *RMja* and so confers resistance to *M. javanica*. Nevertheless, the poor *Prunus* ability to transformation, the peculiar recalcitrance of almond to produce roots spontaneously together with the large size of *RMjaGC2* are so many obstacles to engineer *RMja*-transformed roots and so validate the *RMjaGC2* gene. Its transfer in species with a more favorable genetic background would be an alternative. In this perspective, preliminary works to transfer *Ma* in tomato failed to provide any resistance, even though a constitutive transcription has been detected (H. Duval, unpublished). Missing components in *Solanaceae* probably explains the unsuccessful transfer of resistance, which seriously restrains this approach at least in these models. The dual transfer of *Arabidopsis* NBS-LRR genes, *RRS1* and *RPS4*, circumvented the restricted taxonomic functionality (Tai et al., 1999) in *Solanaceae* species (Narusaka et al., 2013). In the future, the identification of a *Ma*/*RMja* partner could help consequently to break down the technical barriers that are obstructing deeper functional analyses.

### Insight Into the Molecular Determinants Involved in RKN Resistance

The *Ma* gene is not circumvented so far and confers resistance to mitotic species *M. incognita, M. javanica, M. arenaria, M. ethiopica, M. enterolobii* and the meiotic *M. floridensis* (Claverie et al., 2011). A recent study showed that *Ma* and *RMja* resistance spectra are closer than expected. Indeed, the sole species uncontrolled by *RMja* (*M. incognita* and *M. floridensis*) (D. Esmenjaud, unpublished) are also close in the phylogenetic tree of the *Meloidogynidae* family (Holterman et al., 2009). The weak polymorphism between *RMjaGC2* and its peach ortholog suggests that few mutations may confer resistance to most RKNs. The differential specificity between *Ma* and *RMja* may be due either to the gain of recognition of a ubiquitous effector from *Meloidogyne* spp. in *Ma* or to the loss of recognition of an effector specific to *M. incognita* and *M. floridensis* in *RMja*. In the case of a direct recognition of effectors by NBS-LRRs, few mutations may lead to extended recognition specificity or, on the contrary, to a failure of recognition due to a rapid co-evolutionary arms race (Karasov et al., 2014). In this context, we should observe among *Prunus* accessions a panel of alleles conferring variable resistance interactions to RKNs for species specificity and/or resistance strength.

Structurally, the three genes *RMjaGC2*, *Ma* and *Prupe.7G065400* display an identical repetition of five PL domains. The cost for the plant to produce and conserve such massive C-terminal ends in *Prunus* species is in line with an essential role of these repeats. PLs may function as baits mimicking more or less fitted RKN effector targets and thus may reveal quite original immune mechanisms in woody perennial plants. Screening identified RKN effectors [reviewed with their potential targets in Truong et al. (2015)] for Ma recognition is a strategy that should help to clarify the RKN resistance mechanism in Prunus. Even though the role of the PL domain is still unclear, mutation in a conserved PL-domain motif (i.e., the second motif defined in Van Ghelder and Esmenjaud, 2016) of the Arabidopsis *RPS4* gene, impaired the dual-*RPS4/RRS1* mediated resistance suggesting a role of this domain in immune signaling and/or partner interactions (Sohn et al., 2014).

### Position and Role of the 5-PL-Containing Genes in the Resistance Mechanism

*Ma* and *RMja* are single homologous genes conferring resistance to different RKN species. Nevertheless, other partners could also be actively involved in this process. The orthologous genes *Rpi-blb2* and *Mi-1.2* from potato and tomato, respectively, conferred resistance to certain RKNs, aphids and white flies (van der Vossen et al., 2005). Recent studies showed that these two genes required other NBS-LRRs (NLR required for cell death-helpers or NRC-helpers) to function. Besides, transfers of diverse tomato NRC-helpers together with *Rpi-blb2/Mi* in NRC-silenced *N. benthamiana* showed contrasting rescues of *Rpi-blb2/Mi*–mediated cell death (Wu et al., 2017). This highlights the influence of the NBS-LRR sensor-helper affinity to transfer successfully a complete resistance and interrogates about a model in which *Ma, RMja* or *Prupe.7G065400* sensors would confer distinct resistance spectra in accordance with a divergent affinity between sensors and helper(s) in plum, almond, and peach.

As crucial elements of PTI, kinase proteins are effector targets, which will modify and alter kinases activity favoring pathogen growth and development. The conserved lectin S-domain receptor kinase in the R region, which mostly segregates with Ma orthologs, is classified within the *Galanthus nivalis* agglutinin (GNA) homologs (Eggermont et al., 2017). Some members are known to be upregulated in presence of pathogens while others are presumably involved in calcium influx, which is one of the PTI characteristics. At the same time, many targets of RKN effectors are unknown, even in the well-described *M. incognita* calreticulin effector Mi-CRT, which is crucial for infection by possibly altering calcium homeostasis (Jaouannet et al., 2013). In this perspective, the lectin kinase might be targeted by RKN effectors and monitored by Ma/RMja as illustrated in the kinase/NBS-LRR (PBS1/ RPS5; Pto/Prf) models in which kinase modifications induced by bacterial effectors are detected by NBS-LRRs inducing immune responses (Zhang et al., 2010; Ntoukakis et al., 2014). Interestingly, the CNL *Prf* also lies in the middle of the kinase *Pto* cluster (Salmeron et al., 1996). As, effectors can target different kinases, some NBS-LRRs can monitor multiple kinases (Lewis et al., 2013). Unlike direct recognitions, the indirect strategy potentially provides a wider protection against pathogens (Cesari, 2017) and may be favored in long-lived organisms. Guard components will be potentially modified by effectors in different ways (e.g., phosphorylation, acetylation, cleavage, etc.) and consequently detected by NBS-LRRs proteins as illustrated by the RIN4 guardee/RPS2-RPM1 CNLs model (Mackey et al., 2003). In this case, the only way for the pathogen to escape recognition is a loss of function of the effector, which may be insurmountable and lead to durable resistance.

### Expression and Regulatory Region

The predicted regulatory region of *Ma* is large and complex. Nevertheless, the transfer of *Ma* together with the 5.4 kb region localized immediately upstream the ATG in susceptible plants, was shown to be sufficient to confer the resistance (Claverie et al., 2011). The pattern of *cis*-acting binding sites is very similar between *RMjaGC2* and *Prupe.7G065400* putative promoter regions but quite different from the *Ma* region. The WRKY transcription factors, which bind to *cis*-regulatory W-boxes, respond to activation of abscisic acid-dependent pathway and belong to SA signaling that are both linked to NBS-LRR regulation (Eulgem et al., 2000). We found more W-boxes in the *Ma* regulatory region than in the *RMjaGC2* region. The *Prupe.7G065400* upstream region is clearly the least endowed with W-boxes. The insertion of the *Prupe.7G065500* gene that does not exist in plum and almond might disturb the regulatory region of the downstream *Prupe.7G065400*. Beyond the expression level, the sensitivity of the ON/OFF equilibrium of NBS-LRRs plays a crucial role to trigger or not the resistance. Indeed the switch toward an ON state of the *L6/L7* alleles of the *L* locus is governed by the ligand affinity and the intramolecular constraints existing between TNL domains (Bernoux et al., 2016). These attributes defined how NBS-LRRs may cross the threshold conferring them the ability to produce, or not, cell death and/or resistance (Zhang et al., 2017).

We showed that *Ma* orthologs exhibit various and sizeable intronic microsatellites upstream each PL-coding exon with a particular feature in the last intron (PL4-PL5). This intriguing structural characteristic is present in 60% of the PL-containing TNLs in peach (Van Ghelder and Esmenjaud, 2016). We do not know yet if the presence of SSRs exclusively before PL-coding exons is only a consequence of genetic processes or if this structure plays a role in the modulation of TNL expression or splicing. Besides, several studies established a direct link between microsatellite size in intron and certain human diseases (Kersting et al., 2008; Suryadevara et al., 2013). Although the precise mechanisms are not fully understood, the microsatellite expansion seems to disrupt the targeted gene transcription (Bidichandani et al., 1998).

### The R Region Is a Combination of Dynamic and Preserved Loci

Transposable elements (TEs) are key factors in the birth-and-death model of R genes (Michelmore and Meyers, 1998). The increase of the KIN35-LRR25 interval size between peach and almond is due to TEs insertion. TEs disrupted the TNL environment inducing premature ends (*Prupe.7G065300, RMjaGC3*), quasi-total disruption (*RMjaGC1*) or NBS-LRR split (*Prupe.7G065500 - Prupe.7G065600*) as also reported for *RMia* candidate genes in peach (Duval et al., 2014). The lectin tyrosine kinase is a highly preserved milestone contrasting with NBS-LRR cluster dynamism. Interestingly the kinase ortholog in Arabidopsis (*At2G41890*) lies in a region of the chromosome 2 that lacks the TNL cluster as the co-localization of the kinase and the *Ma* loci emerged in the *Prunoideae*. Our cluster study revealed two conserved TNL loci and other TNLs originated, disrupted or disappeared through recombination events. Evolution studies of R genes in the Arabidopsis genome proposed segmental and ectopic duplication as mode of NBS-LRR dispersion (Baumgarten et al., 2003; Leister, 2004). A ratio estimation of each mechanisms in three legume genomes revealed that local tandem duplication is the main mode of expansion (>75%) before ectopic or segmental duplications (Shao et al., 2014). Regardless of the type of mechanism, NBS-LRR duplications induce phenotypic variations through the diversification of sequences, and therefore in potential functionality, but also in gene expression (Wang et al., 2012). As illustrated in the *Ma* cluster, the modular structure of TNL genes together with a combination of partial duplications and deletions shape the TNL diversity. The chimeric *MaGC2* gene originated from an incomplete gene duplication of the *Ma* and *MaGC3* genes. Interestingly this gene includes a frameshift mutation close to the recombination site, making it prematurely truncated and perhaps preventing deleterious effects due to auto activity. In this cluster, PL-coding exons are prone to intragenic duplications creating high sequence diversity. While *Prupe.7G065500* underwent PL2 duplication, the *Ma* orthologs underwent PL1 duplication followed by rapid PL3-PL4-PL5 duplications.

Finally, our mapping pointed out a restricted region conferring resistance to *M. javanica* in almond. The functional annotation, the genetic resources related to *Ma* and the peach genome, together with the transcript expression analysis strongly support that the *RMja* gene is the *Ma* ortholog. In a dynamic environment, the high conservation of the three orthologs, either conferring a complete-spectrum resistance (*Ma*/plum), or conferring an incomplete-spectrum resistance (*RMjaGC2*/almond), or conferring no resistance (*Prupe.7G065400*/peach), argues for a major role of these genes. Our study also identified the highest gene polymorphism in the atypical PL region that might be involved in the RKN resistance spectrum. These results pave the way to reveal the role of the PL domain and a putative original immune process for RKN control in *Prunus*.

## Data Availability Statement

All relevant data is contain in the manuscript or in **supplementary material**, except the almond genome sequencing reads and the BAC clone sequences.

These datasets are publicly available in the NCBI BioProject accession number [PRJNA448729] [https://www.ncbi.nlm.nih.gov/bioproject/PRJNA448729] (BioSamples numbers [SAMN08863554] [https://www.ncbi.nlm.nih.gov/biosample/SAMN08863554/] and [SAMN08863555] [https://www.ncbi.nlm.nih.gov/biosample/SAMN08863555/]) and in the NCBI BioProject accession number [PRJNA448736] [https://www.ncbi.nlm.nih.gov/bioproject/PRJNA448736], respectively.

## Author Contributions

HD, DE, and CVG designed the research project. HD, DE, MM, and CVG carried out and supervised the greenhouse and laboratory works. ED supervised and carried out the sequencing of almond genomes and produced the alignment files. CC supervised and carried out the almond genomic library, selected BAC clones and produced BAC clone sequences. HD supervised the data releases, CVG planned and designed the sequence analyses, analyzed the data, and drafted the manuscript. CVG and DE revised the manuscript. All authors commented and approved the manuscript.

## Conflict of Interest Statement

The authors declare that the research was conducted in the absence of any commercial or financial relationships that could be construed as a potential conflict of interest.

## Footnotes

1 https://github.com/PacificBiosciences/Bioinformatics-Training/wiki/HGAP

2 ftp://ftp.bioinfo.wsu.edu/species/Prunus_persica/Prunus_persica-genome.v2.0.a1/assembly/
