# Supplementary material for: Ma Orthologous Genes in Prunus spp. Shed Light on a Noteworthy NBS-LRR Cluster Conferring Differential Resistance to Root-Knot Nematodes
Source: Front Plant Sci. 2018 Sep 11;9:1269. doi: 10.3389/fpls.2018.01269 (PMC6141779; doi:10.3389/fpls.2018.01269)
Supplement: Supplementary file 1 [file Table_1.DOCX]

**Table S1**: List of genetic markers and primers used in this study.

| Molecular markers  *5'-fluorescent-labeled oligo | Sequences | Localisation on the LG7 of the peach genome v2.0 | Allele sizes  R / S (bp) |
| --- | --- | --- | --- |
| CPPCT022F*vic  CPPCT022R | CAATTAGCTAGAGAGAATTATTG  GACAAGAAGCAAGTAGTTTG | 10 612 187  10 612 485 | 244 / 229-263 |
| Kin35-F1*pet  Kin35-R1 | ACGCTTGTTGCGCTCTTAAT  CGCGCAAACTCATAAAGACA | 10 449 670  10 449 429 | 280 / 248-260 |
| ENDKIN-F  ENDKIN-R | CCACTTGAGGAAGAAGAGGAG  AGGAGGTTTGGGTGGTCAT | 10 408 974  10 407 779 |  |
| LRR5-F  LRR5-R | CGGCAGCAGAGGACAATTAT  TTCTCAACGTCTCAGGGTGT | 10 375 633  10 375 866 |  |
| LRR25-F1*  LRR25-R | GGCTTGCAATCAAATCAACA  GTGAATTGAATGCGACCACA | 10 350 796  10 350 535 | 257 / 255-259 |
| LRR65-F*  LRR65-R | AGAGGATAGGGAGCAACTCATT  CGGAAGAATCTAGAGAGACTGACA | 10 310 694  10 310 513 | 160 / 190 |
| CPPCT050F*fam  CPPCT050R | CAGGACTAGCAAAGCCGAAG  CGGAAATGGAAACGGTTTAG | 10 045 750  10 045 603 | 154 / 176-186 |
| CPPCT039F*pet  CPPCT039R | GCACCAGTTCTTCGTCATCTC  GCATGCATAAAACCTTTATTGG | 8 338 230  8 338 130 | 101 / 99-123 |

| Primers | Sequences | Localisation | Tm (°C) |
| --- | --- | --- | --- |
| TIRRMjaGC1F | CCATTTCGACACTCTTGAAAGC | RMjaGC1 | 59 |
| TIRRMjaGC1R | ACCTCTTACATCAAATCCTGAGAGG | RMjaGC1 | 59 |
| TIRRMjaGC2F | GCCGAAGGATTTGATCTGAC | RMjaGC2 | 58 |
| NBSRMjaGC2R | cctttaaatcatcatttgagaatg | RMjaGC2 | 56 |
| ORF5F | aagaacagcaggacatgtttc | RMjaGC2 | 55 |
| LRRF1_RC | AGGATTAGCTGCTCAAGGTT | RMjaGC2 | 54 |
| EPISLRRF | tacctgaaagcatctctcagc | RMjaGC2 | 55 |
| PL2F2_RC | AATAATGAGGAGTCGATGTGA | RMjaGC2 | 53 |
| PL3F | GGTAGGATCATCTAGTATAACTGGC | RMjaGC2 | 55 |
| PL4F | GGTATCATCTAGTAGGCCTAGCAG | RMjaGC2 | 56 |
| PL5R1 | AAGAGAGACAAGGTATCAATGTATT | RMjaGC2 | 53 |
| PL5R2 | GTTATATATGGAGTGACGATCAAAG | RMjaGC2 | 54 |
| PL5R3 | AGATTGTTGCCGAGGAGTCAT | RMjaGC2 | 59 |
| TIRRMjaGC3F | GCCAACCTCTCGGGATATGAT | RMjaGC3 | 60 |
| NBSRMjaGC3R | AGTCCCTATACGACTCATGTTGGAT | RMjaGC3 | 60 |
