# Supplementary material for: Ma Orthologous Genes in Prunus spp. Shed Light on a Noteworthy NBS-LRR Cluster Conferring Differential Resistance to Root-Knot Nematodes
Source: Front Plant Sci. 2018 Sep 11;9:1269. doi: 10.3389/fpls.2018.01269 (PMC6141779; doi:10.3389/fpls.2018.01269)
Supplement: Supplementary file 2 [file Table_2.DOCX]

**Table S2:** ORFs identified in the KIN35-LRR25 interval of the peach genome

|  | **HMMSCAN Results** | Localisation on the pseudomolecule 7 of the peach genome v2.0 | Peptide size |
| --- | --- | --- | --- |
| Prupe.7G066100 | MATE efflux family protein | 10 448 921 – 10 455 714 | 469 aa |
| KIN35 | SSR marker | 10 449 429 – 10 449 670 | marker |
| Prupe.7G066000 | cytochrome P450 | 10 446 745 – 10 448 654 | 340 aa |
| Prupe.7G065900 | GAG-pre-integrase domain | 10 443 511 – 10 444 585 | 139 aa |
| Prupe.7G065800 | Ankyrin -zinc finger CCCH | 10 431 283 – 10 435 753 | 703 aa |
| Prupe.7G065700 | Tyrosine kinase | 10 408 597 – 10 413 116 | 753 aa |
| Prupe.7G065600 | TIR-NBS-LRR | 10 403 063 – 10 407 797 | 729 aa |
| Prupe.7G065500 | TIR-NBS-LRR | 10 392 103 – 10 399 974 | 1890 aa |
| Prupe.7G065400 | TIR-NBS-LRR – Ma orthologue | 10 381 090 – 10 389 382 | 2027 aa |
| Prupe.7G065300 | TIR-NBS-LRR | 10 375 757 – 10 379 123 | 853 aa |
| Prupe.7G065200 | ATPase | 10 358 144 – 10 360 983 | 168 aa |
| LRR25 | SSR marker | 10 350 535 – 10 350 796 | marker |
| Prupe.7G065100 | Guanylate kinase | 10 348 684 – 10 355 424 | 411 aa |
