# Supplementary material for: Ma Orthologous Genes in Prunus spp. Shed Light on a Noteworthy NBS-LRR Cluster Conferring Differential Resistance to Root-Knot Nematodes
Source: Front Plant Sci. 2018 Sep 11;9:1269. doi: 10.3389/fpls.2018.01269 (PMC6141779; doi:10.3389/fpls.2018.01269)
Supplement: Supplementary file 4 [file Table_4.DOCX]

**Table S4:** Alignment and functional annotation of the amino acid sequence of the Tyrosine kinase *Prupe.7G065700* in peach and its orthologues in the ‘2175’ plum accession and ‘Alnem1’ almond accession.

**Signal Peptide**

2175 MPEQDRLLLKLCFLLCIFAGFLLHSLGASEIPLDSKLSIVDKDMWVSPNGDFAFGFFNSP

ALNEM MPEQDRHLLKLCFLLCIFAGFLLHSLGASEIPLDSKLSIVDKDMWVSPNGDFAFGFFNSP

Prupe.7G065700 MPEQDRYLLKLCFLLCIFAGFLLHSLGASEIPLDSKLSIVDKDMWVSPNGDFAFGFFNSL

****** ****************************************************

**alpha-D-mannose-specific plant lectins**

2175 DEPNYSVGIRSNSKSIPLDKQIVVWIAGADLILGNNSYVQLTQDGELILFDSLKGVIWSS

ALNEM DEPNYSVGIRSNSKSIPLDKQIVVWIAGADLILGNNSYVQLTQDGELILFDSLKGVIWSS

Prupe.7G065700 DEPNYSVGIRSNSKSIPLDKQIVVWIAGADLILGNNSYAQLTQDGELILFDSLKGVIWSS

**************************************.*********************

**alpha-D-mannose-specific plant lectins**

2175 KTRQLSVVSAALNDNGNLVLLNKEKHIVWQSFDTPSDTLLPGQNFSIFQTLRAASKNSVS

ALNEM KTRQLSVVSAALNDNGNLVLLNKEKHIVWQSFDTPSDTLLPGQNFSMFQTLRAASKSSVS

Prupe.7G065700 KTRQLSVVSAALNDNGNLVLLNKEKHIVWQSFDTPSDTLLPGQNFSMFQTLRAASKNSVS

**********************************************:*********.***

S-locus glycoprotein domain

OUTSIDE

2175 SYYTLFMNASGQLQLRWESHVIYWTSGSPSSSNLSAFLTSDGALQLRDQNLKPVWSLFGE

ALNEM SYYTLFMNASGQLQLRWESHVIYWTSGSPSSSNLSAFLTSDGALQLRDQNLKPVWSLFGE

Prupe.7G065700 SYYTLFMNASGQLQLRWESHVIYWTSGSPSSSNLSAFLTSDGALQLRDQNLKPVWSLFGE

************************************************************

S-locus glycoprotein domain

2175 DHNDSVSYRFLRLDVDGNLRLYSWVEPSKSWRSVWQAVENQCNVFATCGQHGICVFTESG

ALNEM DHNDSVSYRFLRLDVDGNLRLYSWVEPSKSWRPVWQAVENQCNVFATCGQHGICVFTESG

Prupe.7G065700 DHNDSVSYRFLRLDVDGNLRLYSWVEPSKSWRPVWQAVENQCNVFATCGQHGICVFTESG

********************************.***************************

PAN-like domain

2175 SPDCECPFKHTNESISRCLIPNHPCDSGTDMLKYMHTFLYGMYPPTDDLVAKVSLQECKS

ALNEM SPDCECPFKHTNESISRCLIPNHPCDSGPDMLKYMHTFLYGMYPPTDDLVAKVSLQECKS

Prupe.7G065700 SPDCECPFKHTNESISRCLIPNHPCDSGSDMLKYMHTFLYGMYPPTDDLVAKVSLQECKN

****************************.******************************.

PAN-like domain

2175 LCLNDPSCIAATFSNDGTARCLMKRTQYVTGYSDPSLSSVSFVKTCAYPLAVNPNHVTTS

ALNEM LCLNDPSCTAATFSNDGTARCLMKRTQYVTGYSDPSLSSVSFVKMCAYPLAVNPNHVTTS

Prupe.7G065700 LCLNDPSCTAATFSNDGTARCLMKRTQYVTGYSDPSLSSVSFVKMCAYPLAVNPNHVTTS

******** *********************************** ***************

TMHMM

2175 PSPLEQSHKFCFPCVIGVASGMFVVFVLVQLALGFWFFRRRNLDRKKAALAYTSPNSNGL

ALNEM PSPLEQSHKFCFPCVIGVASGMFVVFVLVQLALGFWFFRRRNLDRKKAAFAYTSPNSNGL

Prupe.7G065700 SSPLEQSHKFCFPCVIGVASGMFVVFVLVQLALGFWFFRRRNLDRKKAAFAYTSPNSNGL

.************************************************:**********

Protein tyrosine kinase

2175 IVLSFSEIEELTENFKHQIGPKMFKGVLPNKKPVAIKDLNITIEERKYRSAVSKIGSIHH

ALNEM IVLSFSELEELTENFKHQIGPKMFKGVLPNKKPVAIKDLNITIEERKYRSAVSKIGSIHH

Prupe.7G065700 IVLSFSEIEELTENFKHQIGPKMFKGVLPNKKPVAIKDLNITIEERKYRSAVSKIGSIHH

*******:****************************************************

Protein tyrosine kinase

2175 KNLVKLQGYCCELDHRFLVYEYAKNGSVEKYLEDLKLCKKLTWGKRFDICLSVARAICYL

ALNEM KNLVKLQGYCCELDHRFLVYEYAKNGSVEKYIEDLKLCKKLTWGKRFDICLSVARAICYL

INSIDE

Prupe.7G065700 KNLVKLQGYCCELDHRFLVYEYAKNGSVEKYIEDLKLCKKLTWGKRFDICLSVARAICYL

*******************************:****************************

Protein tyrosine kinase

2175 HTSCREFMSHGNLKCENVVLEENLEAKVTEFGLGKVVSEASCSSAERDVEDFGKMVLVLV

ALNEM HTSCREFMSHGNLKCENVVLEENLEAKVTEFGLGKVVSEASCSSAERDVEDFGKMVLVLV

Prupe.7G065700 HTSCREFMSHGNLKCENVVLEENLEAKVTEFGLGKVVSEASCSSAERDVEDFGKMVLVLV

************************************************************

2175 SGCRGVGDLCEWAYREWMEGRPENVADKRISGGFNLQELERSLRIAFWCLQIDERRRPSM

ALNEM SGCRGVGDLCEWAYKEWMEGRPENVVDKRISGGFNLQELERSLRIAFWCLQIDERRRPSM

Prupe.7G065700 SGCRGVGDLCEWAYKEWMEGRPENVVDKRISGGFNLQELERSLRIAFWCLQIDERRRPSM

**************.**********.**********************************

2175 REVVKVLEGTLSVDPPPPPFGCNGPPEEEEEP

ALNEM REVVKVLEGTLSVDPPPPPFGCNGPLEEEEEP

Prupe.7G065700 REVVKVLEGTLSVDPPPPPFGCNGPLEEEEEP

************************* ******
