# Supplementary material for: Ma Orthologous Genes in Prunus spp. Shed Light on a Noteworthy NBS-LRR Cluster Conferring Differential Resistance to Root-Knot Nematodes
Source: Front Plant Sci. 2018 Sep 11;9:1269. doi: 10.3389/fpls.2018.01269 (PMC6141779; doi:10.3389/fpls.2018.01269)
Supplement: Supplementary file 5 [file Table_5.DOCX]

Table S5: Sequence of the KIN35-LRR25 interval of the R accession ‘Alnem1’

>Kin35-LRR25

ACGCTT

GTTGCGCTCTTAATCAATTATTTTTTATTTTGCTTCTTGTTTACCGGTGGTAGAATAGAT

GTCTGTACAGACAAATCTCTCTCTCTCTCTCTCTCTCTCTCTCTCTCTCTCTCTCTCTCT

CTCTCTCTCTCTCTCTCTCTCTCTCTCTCTCATTATTCTCTGTTAATTCCAGGTTTTGGT

GGCTTTAGTGAGCATATTGTGCCTATTTCTATTATCAAGCCACGGCTTTGTAGGTATCTG

GGTTGCTTTATCTGTCTTTATGAGTTTGCGCGCACTGGTTGGATTTTGGAGGTAAATATC

CAGTTGAACTAAATTGTTTAACAAAGATTGTTGTCATAACATATTTTGTGATTTGTAAAT

TCAAAGTTGCTTCAACTGCATGAAACCTTTTGCAGGATAGGAACGGGAACGGGACCATGG

AGCTTTCTTAGGGAGTAAATTCTGAATAATATGTTTCTGCATATATTCCTTCTTTTCCAA

ACATTATTTATTGTCGAGCATGTCTTACAAGCTCACCAGAAGGGCAAAGAGGAAACTCTT

ATGAGGGGATGATATTACTGATATGCCATCACCATTTCTTGTTTGGGGATGATTGAGATA

ACCACACATAGGGGTGGGCATCGGGACAGGAAAATCGGAACACTGAACCGAACTGGTGAA

AAAACTGGAAAAAAATCGGTTGACCAAAAAAGTCAACAAACCGGACCAGAACCAGTTCCA

ACCGATTTCGGTTTCAGTTTCGGTTTTACATCCCACCGCACCGGACCGGATTGAACCGGA

CCGGTCATATATATATATATATATTTAAAATTTAAAAAATTAATAATCCGGTTCAAAATC

GGAACCGGTCAGAACCGAATCAGTCAATTTTTGAATTTTTTTTTTTGCCTAAACCGGATC

GAACCAGACCGATTAAATAGTAACGGTTCCTATTTGAGGTGAGAACCGGACCGAACCAGA

CCGCCTCCACCCCTAACCACACATATGTAATGAAAAACAAGAGAGCACCATTCTTTTACT

GCTTTCTCAAGTTAGGTCGAATGAATCGTAATTGGTGATCTATACTGTTGTTTATGTTCA

CGCGTGAATTATGATAAGGATTGGGTGATTGTCAGCCGTTGATGCATGTAGACTATTATC

AGCCGTACTCACATGCATCAACAGTTAACGTTCATCTAGAAGTGTGTGGGCAACTATTCT

TAGCACCCCACAAGTTCACGTCCTTTAGCATCCCCAGTTAACTAGATGGCAAACTAAATC

CCTTAAAAGATCTTATTTACCATGTTGATGACACTAACTTATCTGATAGATGAGATGGCA

ACTCACATCATCTACCACGTGAGATGGCACAAAAATAAATAGAAAACCACTTACAAACAA

CAGCAGCAGCGACAACAACGCTGTATGTAGTGTAGTGATCATCAGGGAATGAAAGGAATG

GCGATAACATACAGGTCATTCTTCTTGATAGCAGCCAACTTGATAGGTTCCGTCATGTCA

AACTGAACCTGTTGCTGTTGCTCTTGTTCTTCTTCTTCTTCAGTGGCCTTAAAGGCATTT

TTTTAAGACAACAGGAGTGCAGGGGGGAGTGAGTTTCCAGTTAAAGTGGTAGAGCAGTTG

AGCAAGTGCAAGCTCAATGGATGTAGTTCCAAATAATATGCCTGTACACATTCTCTTACC

TCCCCCAAATGGAATAAATTTGAAGTCATTCCCTCGAAAATCAACCCGACAACCTTGGAA

CCTGTCAGGCATAAAGCAATCAGGATTTTCCACATCCCTCAAGCATTGATTAACACTCTC

GATTTACTTGGTATATCGTACCCGCCAATTTTATGTAATAATAAGCCGCTTTGGTCATCA

TCATCTGCTTCAGCTAATTGCCTTGGGATTAAGGAAAGAGGAGGGTGTAATCATAGAGTT

TCTTTTATCACCATCTTCAAGTAGTCTAGCTTCTTCATGTCTGCATCTTCAAGTTTTTTC

TCATTTCCCAATCCTGCAACAACTTGGCGTACCTCCACCTGTGCTTTCTCCATTGCTCTA

GGATTTCTCACAAGTTCTGACATTGCCCATTCTGTGGTAGCCGCTGCAGTCTCACTTCCA

GCAAAATACAAGTCCTATAAAAATGAAAGAAAAATAAAAGTACTTTTATTCACTTAATAT

CATTTTCATACCAACTAATTATAATGTCATGTCCTGATGACCTTGACAGAACAGAAATTA

ATTAGTCAAGAAACAAAAGAAGTAGATTATAACACTCATAGAGGTAACGTTATGCACATA

CGTACCAGCGTGACAGCTTTGATTATGGTGTTTGTGAGATCAATTTGGAGTTCACCAGAC

TCATGGAGTTGTAAAAGCACATCAACTAGATCTTCTTCTTCTTCCTGATGGCATGATTCT

TTGTTATTTTTGTTGGTTAATAATGCAAAGGTGGCCTTTCTTTTCTTTCTATGGTCATCG

ATGACTTCCTCTAGAATCCTGTCCATCTTTCGGTATATTTTCTCCGAGGCAGGCTTCAAA

CCAATGACATAACGAAGGAGCTAAAGCGAAGGGAAGAGATCAGGCAGATCAAAGCCCGCC

GCGAACTTTCCCATTTCCTCCACCAATGATATAAATTCTTGTTGATGTTTGCACTTTTTC

CCCAAGGCTGCACGGGCAATAATGCAATTCTGCATGTTGAAGATCATGTCGCTAAGATTG

ATAGGATGACCCTCAGATGAGGAGATTGATTGAACTAGACTCGATGCCTCTTCGTTTCTT

ATCGATGAAAAAGACCACACCCGTTTCGCGCTCAGGAGCTCAAGCATGCAAACCTTTCTC

ATATCTCTCCAGTGATCATTATCAGGAACAAAGATAAGACCTGCACAATTGTAGGAAAAA

ATTTCAGCAGCGATAAGAGCTGGCCGGCAGTTCGGAGAAAGCGGCCTTGTTTATCTTCAA

CACCTCTTCAGCTAACTGTGGTGATGAAATGACTATGGGGTTGATACTTGTCACAGTTTG

AGGTGCATGATAGGCCCATACTTCTTGGCCAAGTCTGTCAGGCAATGATGTTGTAAAGAG

CCAGCCAACTGGTGCAAGTTTCCAATCAGACGTAGCTTCCATGGCTCTGGAGGCAACCTA

AGATCAATTGGACAAGTATGGCTTCTAAATCTCTTCCAGTATATAGCAAGGACAACAAAG

AGCACCAGAGAAGTGAAGAGAGGAAGGGAAGGACTTCGGATTATTTGGAGCATCATCTGT

GACACAAATGAAGAAGCTCTTGTCATTATAATCATGCATCTGTTTTTCTGGCAGAAGCAT

TGGTTTTAAAATGGGCACTTTTACATGCTTCCAGGCTTCCTTTACAATATCTGGATATTG

AAGATTTTTTGGCAATTTTTACGATTTTTTGGCTAATAGCTGACAGGGCAAGGCTGCTGA

TGTGTCAAGACTGTTGCGTTAATCATGGGTGTTAGTTTGTATTTAGCTGTTGTAATAGTC

ATGTGTATCTTTCAATTAGTTTGGAAGAACTAGCTTAATTACTAAGCAAAGCCCAAAGTA

TAAAGGTCTCTTGTTACTGTAATATTCATGAATTGGAAAAGAAATCTCATATACTATTCT

CATTTTCTCTCTCTGATTTCTCGATTCTTCTTCTTCTCATTTCTTCCTCAATCGTTAGCA

TGGTATCAGAGCAGGGTTCGATCCTGGGACCTGTGGGTTATGGGCCCACCAAAACCTAGC

TCAATCTACAGGCTTCTATAGTGGCTTGTACACTCTAATCCTGATGTGCTTCCTCTGTGT

TTAGCTCTGATTTCATGTTCTCTTTCTTTCCCCTTCTTCAATTTCACGTCAATTTCGTCA

ATTTCATGCTCAATTCCTTTCCTGTTTGTTGAATCAATTTCTTCGATTTCATGCTCAATT

TCTTTCTCAATTTCACGTCTTCTTCTTCGATTCCAAAACTCTCATCTTTCTTCTTTGATT

GTTAGACAAATTCATCTTCTAAGGTTTCTTTGACATTCTATTTTTCTGCATCAATGGCGG

CTTCATCTCTCAAAATTGAAGGATTGCTAGGAATGGTTTGTTGAGAGTGTTTGCCCCACA

TTGAAATGTTAAGGGACCTAGCCTAGGTTTATAAGGAGTTGGCTACTCCGCTTGTAAGAA

CCCAAAACAAAATATCTAAAAAAGAAAGAAAATATCTAAAAAGTAAGGAAAATATCTATT

AACAAAAAGACCAATTTGCCCTCGCATAATTTAATGGGGGAAAATTTGACTTTTTAATCG

AGAAAGAATTTGGGGATTCCGCTTATGCCATTGCGTAGAGCGCGGCGAAATGAGTTCGTA

GACACGGAGTAGACCCGAATCGGAGCTGTAACGAAGAAGTTATGGTCTAAAAATCACGAA

GGGCAAAATGATAATTTGGTCAAAAAGTCAGATTTTTATAGCGCTCTCTCTTCTCCCCCG

TCAGTTCTCTCTCTCTCTCCTCTCTGTCTCCTTCCGCGCGATTTGGGTCGTGCGACCCTT

CGCCTTCCAAGCGACGGCCCGGCCACCACAGGCCGAGCCAATCTCCGCCGTGGGTACCAT

CGGGTCCGCCTCCGTGTCGCCGTCGAAACCTGACCAACCTCCACCCCGGGGCCGCCCTGA

GATGGCCGGAAAATCGTATTTTCCTACGGAGGTTCGTCCGAAGCTACCCAAACTTCCAGC

TCGAAATTCTCCTTCGTTTCTCCACCAAATCGATCGAGTAAGGTATGGTTTCTCAGCTAT

TTTTCGTGCTCTAGCTGATGGGTATATGGGCTTCAATCGATTTTAGCTCTAAGGGGTTCG

ATTTTCGACTTGAAATTTGGCCGAAACTTCGGCCCCCCGAAACTACTGTTTCTGGTCACT

TTTTGGTGGTTGTCCAAGAACAAAAGTGACTCCAAATGGGGTTTTTCACCTAGGATAGGA

GTTTGGAGTCTTGGTTCCGAGATTTTTCGGCCACCCAAAATCGCTTTGGACACCCAAAGC

TGCCCGCGCGTGCTGGAGCGCGTGGGCGAGGGTAGTGATGTAATTCTGTGCAGTTTTGTG

ACCCTCGTGTCGTCACGAGCGCATAGGATTTCGCGGATCTCGATTCGGAGTCCGTTTGAA

CCCCAAACGGATTTTTCATATCGCGCGATCCGTGGGTGCAGTGTCGTTAAATTGTCGGAT

CGCGCTGAATCTCGGATATGTCGGTCTACACGACATCGGGATCGTGTAGGATTTGACGGA

TTTCGAATCGGAGTCCCGGATACTCCGGAATCGCGAACCCTGGGGCTAGGGTTTGGATTT

TAAGCGATAACGCGATTTTGGCCAATCCGACCGTCCGTTTTGGACCAAACTCGTAGAACA

TGGTTCCCTCTCTATAAGGAACCTTCAGGGAAACCCAGATTGGCCATCGGAGGTCGTGGA

CCCACAGGGTCCCGGGTCGGTCGATCGGGCAGTGTATCGCTTAGCTGAGCGTCGGACCCT

TTAAAACTGGTCCAAGTGTCTGAAAAGGCTAGTGTGGGTTCAGGAGTAACTTGGTTTTGA

ATGCACGTACTTCTAGGAGCCGGGGCAGGGTGTTTAATTTAATTCTTTTATTCAGCGGTT

TAGTGATTTACTATTCATGGATAATCAGGCATCAGAGGTCCAGCCGATCAGCAAGAGGAA

CCTTCAAAGGGTCAAATTAGCTCGGACCATCTGTGAGTGGACTTTCTTTCATGAATTATT

TTATATAAATGAGTTATATAGAGGTTTTCCATAAATAAGATTTTATAAGTGATTTATATT

GATTTTATATAAAAGAGTTTTTATAAACGAGTTTATTTGACTAAATTCCTTATTTGTTCC

TGAGTAAGCTTTATTGTGTTTTCGAGCATGATTAATACAGAAATATTTCTATAAGTTGTG

TGCTGCTTACTTTTACATGCTTAAGAGTTATAGAAAACAGATTTTGAGGCTTATGAAGAA

ATAGCAGCACAATTTGAGATTTCAGTTGTTATTCAGATTTTTCTAAAGGATTTTATTTTA

AAACCACCTCGTACCCATTAATTTTTGGTGATTACCCAGAGTTGGACTGATGTCTACGGA

CATCCAGTCCGATTTCAGTTATGTCAGTGCACTTGACTTTGCCTCACGAGTTTCGGGGAT

GCTCGGACTGTGAGTGCCAGGATTTGCGGCTCGGCAGACTTGGTGTCCCGAGACCTGCCA

GGATTGCGGCTCGACTGACTCTGTGTCCCCTAGACCTGCCAGGATTGCGGATCAGGCTGA

CTACAGTCCCCTGCATCCTGCCAGAGCGACTCGAGTTGACTTGGTGTCATCGAGGAATCA

GCCGGCGGGACAGGCTGATCATTGTCTCCTGATTTCGCCAGTTTGCGGCTCAGGTGGACT

GCGTAGCGCCCGAGACCTGCCAGCATAGACGGATCCACCATGGGGTCAATGGGGTCAGAT

GACCCCATGGCAGATCTGGAATTTAGTGGTAGGGGCTGGTTTTTGCCCTTGGCAGCCATG

AAAGTGACCCCATGGAGAAGAAGAAGAAGCTTCGGTGGTGGCAGCTGGAGCAAGAAGAAA

GAGAGAGAGAGGGAAGAGAGATGGAGCATACGGAGGAGAGAGAAGAAAGAAAAGAAGAAG

AGAGGAAGAAGGAAAAAGAATAAAAAAAAGAGAGGGGAAAAAGGAAGAAGGAAAAAGAAT

AAAGAAAGAGGGAGGCGGGAGGAGGGAGAGCGAGAAATAGGAAAAAAGAATAAAAAAGAG

AGGGAGTGATTAAAAAGAGAGAGAGGGGAAATATAATAATTTTTTTTTTCTTTGTTACCT

TTCAATTCCTTCCAAATATTTCAACAATATTATCAATAATTCCATAATTTTCTCTATTTC

AAATGTCGTATAATAACAAAAAGTTCTAAATATGATGAAGAAAATATAATGAAAAAAGAT

TGCTTATATAAAATAAAAATATAACAACAAGGGCTTATAATTTATCCCAAAAAAGAAGAA

AAAAAAAAAAAACCACTCCTGTGGGTTACCTCTAGTTTCCAGTTCATACAATCTATTGTT

CAAGCCTAAAAATCTACAAAAACTCACCAAATTTTCTTAATACATTTGTACTTAAGGTAA

ATTTAGCCCGCCCGATTTGCAATTCCTGGGTCCGTCCCTCCCTGCCACCAATATCTCATA

CCTTGACATCGCCGCCAAGGGTGCTAGCTGCCGTTCACCAACAGCTGTCTCCGTCGCCGC

CACTATTTTTGTGACCCCACGAGTGGCAATTCCTGCATCCGCCACTGCCTGCCAGGGAAT

TGACGGATATAACAGGAGTACAAATAGGTGGTATTTTCAAAGGATTTTGAGTTTTCTTTT

ATTCAAGGATAACTTTCAGTGATTTTATATCGGTTTCTTAGTATTTGCACATTTATATTT

CTATATCTTTATTTGGTTATACCAGTTCTTGATTCCTCCAGGCAGTGATATCTTTATATA

TCTGATCGATTATGTAAGTTTATTCATCAGCATACCGATTCATGCTTATAGAATCTTTTA

TATTGAGATATAGTTAAATTTTCGATATATATATATATATATCCCTAGTTATTTCAAAAC

TGGGGTATTATATTTTGGCTTGTTTTTAAGAATTTCACTTTTTGTCCACTCACAGTTTAA

AACTTGTTTTTCGCCTCCAGGTTGTAGAAGTGCACAGGATCCACCACCGGGCCACTCTTA

GCCTCCGCGCTTCAAAGTCAGGTAGAGTTTGTAGAAAAATCCCAGAAAACCCCGTGAACT

TTAGAAACTTGCTCTGATATCGAGTTGTTGTGGAAAAACTGAAACTGGCAAATATTGGTT

TATCCTATTCTGGCAGTTGGTGTGGATTAGTTGAATGTTTAACAGGTGAAAAGTTTTTGG

GATTAGTCAAAATACAGGGGAGACTCTGCCGAAATTTCGGCAGAAGTCTAAGGGAAGTTA

AAAAGAATTTGAGACAAGAAGGGTAAAAAGGTCATTTGTGCCCGACATTGCCAGGTGTCG

GACACGCACAAGACTTGGCTCGAATTTCAAAGTGGAAATTGGGTCGGGTCCTGTCACCGC

TTATTACCAATTGGTTTTGGGGTGGAGCCTCAACTTCCTTCATGGTATCAGAGCGGGTTA

GCACACGTGTGAAAACCGAACGGCCACACGTGCTCAACGTCACCCAATATGTGTTGTCCA

TGTGTTAAGCTTGAAAATTCGTCACACGTGTGGGGGCGTGTGAGAATGTGAAGGTAAAAA

GTCCCATATTGGAAAGTTGAGAAACCTAACAAGGGCTTATAAGGAGTTGAGCTACTCCCC

CCATTGCCAATAGGTTTTGGGATTGAACCTCAACTTCCTTCATGGTATCAGAGCAGGTTA

GCCCACGTGTGAAAGCCCAACGGCCACAAGTGCTCTACGTCACCCATGTGTTGTCCACAT

GTTAGGCTTGAAAATTCGCCACACGTGTGGGGGCGTGTGAGAATGTGAAGCTAAAAAGTC

CCACATTGGAAAGTTGAGAAACCTATCAAGGGCTTATAAGGAGTTGAGCTACTCCCCCCA

TTGCCAATTGCCAACTTCCTTCATGGTATCAAAGCAGGTTAGCCCACGTGTGAAAGCCCA

AAGGCCACACGTGCTCAACGTCACCCAATATGTGTTGTCCACGTGTTAGGCTTGAAAATT

CGCCACACGTGTGGGGGCGTGTGAGAATGTAAAGGTAAAAAGTCTCACATTGGAGAGTTA

AGAAACCTAGCAAGGGCTTACAAGGAGTTGGGTTACTCCCCCCAACGGCTCATGATCTGT

TTGGCCCAACGGCCACACGTGCTCCCACGTCACCCAATATGTGTTGTCCATGTGTTAGGC

AACCTCAACTTCCTTCATGGTTACTCTACATCTGAAAGATTATAATTTCTTGAAATGGCG

GTATCAGCTTGAATCTGTCCTGGAAGGTTATGATCTTTTCGGCCATTTCGATGGATCTAC

CATTGCTCCCCCAAAATTTGCTATTTTGGATGAAGAATGACTCGCCTCAGAGGTTACTGC

AGCGGATAAGGAGTGGCTTCGGGCTGATAAAGCGTTATTGAGCTTTCTGATTGCAACACT

CTCGGACGACGTGATTGAATATGTTATCGGCAGTAAAACGGCTCATGATGTTTGGTTGAG

CCACTCCGATCGCTATACCACCGTGTCTCGTACTCGGATCAATCATCTCAAAACTGAGTT

ATAGACGGCTAAAAAATGTGGTGATTCAATCGATAAATTTCTCCTGTGTCTATACATATC

AAGGATCAGTTGTCTGTTACTGGAGTTTCAATTTCGGATGATGATCACATGATTGCAGCT

CTCATTGGATTATCTTCCAAGTACGATATGATCAAGACAGTTCTCATTGCTCGAGATACT

TCCATTTCGTTCAAAGAGTTTGGCACTCAGCTTTTAGCTGCTGAAAAATCTGTGCAATCG

CGCCTTTCAGCTCTTCATACTCCTATGGTGGCCATGATTGGTCATACTTCTGGCTCTCTC

AATCCAGGCGCTGGTATCCTTCCCACTCCCTCAACTGTTCCTATATCTCCTTTTGGTCTT

CATGCCAATGCTCAGCCCTCTAGTTCTATGTCTATTTCTTTCGGCAGATGACGGTTTTCT

GGTAATCGGTCTTTTCCTCCTCGTGGAGGTTTTCAAGGCTCCAGACCGTCTCATGGTGTT

TTCCAAAGACTAGTGTTATGCCAGAATGCCAAATTTGCAACAAGTGTGGATATACTGCTG

TCAACTGCTACTATAGGAATGTTTCTCCTTAACACTCAGCCTCATCATCTGTTATTGAGT

GTCAAAATCTGTGGGAAACGTGGCCATGGTGCTTTGGATTGCTTTCATCGCTCAAACTAG

GTGTATCAAGATTCCTCTCCTCCTAAATCTCTCACTGCAATGACAACTCAAACCTCATTT

TCTCTTAAGACATTCTGGATAGCTGATAGTGGTGCCAATCATCACATGGTGCCCACTGTT

GATCAATTGGATTCTGTAACTCCATGTACTTTCTGCAGATCAAGTTATTATTGGCAATGG

GGCAGGTTTGCAGATTGCTCACATTGGCCACACCAATCTCTCTAGTGGCACTTCTAAACT

ACATCTGCACAATGTTTTTCATGTTCCACAACTTACTGCTAAACTTATGTCCGTTAATCA

GCTATGTCAGGATAATAATTGTTCTGTCATTTTTTATCAGTTTGGTTTCTGTATACAAGA

CAAAGCAACAAACCAAGTTCTTCTCATGGGCAGGAGCAATAAAGGCATATATCCTATACC

TAGTGCAGTTTCTCCAGTCAATTCTTCTTCTCTAGCTGATGGTTCTTTTTCTCCAGCTAC

TGCTTATGTGGGCCAACAAATCAAATCATCTATGTGGCATAATAGGTTAGGGCATCCTAC

TAATGAGGTAGTTCAGACTATGCTTAAAACCTCTCAATTACCAGTTCTTGTAGATGCTCA

TCAGCATATTTGCCCTTACTGTTTAAGTGGTAAAATGCACACCTTACCCTTTCCATCTAC

TCATGTTAAGTCTTTACTGCCATTTCAAAGAATCCTTAGTGATCTTTGGGGACCCTCTCC

GTGTAAATCCTATGATGGATACAGATAGGTAGTGACCTTTATTGATGAGTTTACTGGATA

TTCTTGGATATTTCCATTATTCAATAAATTTAAGACCTTCTCAAAGTTTCTTCAGTTTTC

TGCATTTATCAAGAATCAATTTTCTGCATCTATTAAGTGTCTTCAAACTGATGGGGGAGG

AGAGTTTATGAGCAAGCAGTTTACAGATTTCTTGTTGAATAATGATATTGAACATCAAAT

ATCATGTCCTTATTCTCCCCAGCAAAATGGTCTTGCTGAGAGAAAGAATAGACATTTGAT

TGAGACATCTATAACCCTGTTAACTGCAGCTTCTTTGGATGAGATGTTTTGGTTTCAAGT

TATGGCACATTCTCTCTATCTTACTAACAGAATGCCCAGTAGAATACTAGCAAATGAATC

GCCTTATTTCAAATTGTTTCACAAAATTCCAGAGATTAAGCGTCTAAGAATATTTGGTTC

GGTTGTGTATCCCAGTCTGAGGCCCTTAACTAAGCATAAGCTTTAGCCTCGATCTTCCTT

ACGTATGTTTTTAGGCTATATGCTGGGCTATACGGGTGTCATGTGTTATAATATGGAAAA

TCACAAGGTTCTTATTTCTAGGAATGTTTTCCATGATGAAAGTATATATCCTTGTGAAGG

TCATAAGGTGTCTCCTTCTCAGGACACTTTTTTAGTTTCCTGTATACCTATAAGTGTTCC

TTCAAGTGTAGTTTTCCCCACCTCTCATACAGATCCTACACCACATACTACCTTCTTTCT

CAACATCCACAGAGGAGTTTTCTTCTTCTCAAGCCTTTGAGACTTTTATACCTGCAGTTC

CTACTTTGCCAGCTTTATGTGAAGCTCAGTTGGAAGCTTTACTCCCTCATATACCATCAT

CTAGTTATTCTGAGGTTGTTCTCGACCCAAATAGTTCCTCCAGCTCTAATGATTCAAATC

ATGCTAATGATCATCCTATGGAAACTCGAGCAAAGTCTGGTATTAGCAAACACAAGGATT

TCTCAGATTATCATTGTTTTAGTTCGACTTTTCTACCCCCTTCCGAACTAGATGAACTTG

CCAGTTATAAGATTGCTTCTTACTCCTCAGAATGGACAAAAGCTATGCAAGAGGAGATTG

CAGCTTTACATATGCAAGGCACATGGGTTTTAGTTCCACCTCCTTTGCATAAAAATGTGG

TTGGTAGTAAATGGATCTACAAGATCAAACAAAATTCTGATGGCACCATTTCCATATATA

AAGCAAGGCTCGTAGCTCAGGGCACATGGTTTTGATTACTTCAAGACCTTTAGTCCAGTA

GTTAGGCACACTACTGTTAAGAATTTTTTTTTTTTTTTACATGGTGATCTTGATGAAGAG

GTCTACATGAAGCAACCACAGGGTTTTGAAGATCCTCAACATCCAAATTTTGTGTGTCGA

CAAAAGAAATCATTATATAGCTTGAAACAAGCTCCAAGGGCATGAAACTCAAGGTTTACT

AGATACTTACCTTCTCTAGGTTTCAAAGTCTCTCAATTTGATCCCAGTTTATTTGTCAAG

CATCATGGTTCAGATGTAGTCATCTTACTTCTTTATGTAGATGATATAATTTTAACAGGT

TCTAATACAAGTCTCATTCAAGAAATCATTGATGATCTTGGTTCTGTTTTTTAGTTAAAA

GATATGGGGCAACTCTCCTATTTCTTGGGGCTACAAATTTCGTATCAGCATAATGGGGCT

ATAAGAGCAGCTCCACTGATGGAGCCCAGCCCGAGGCAAGGGCAGAAAAAAAAAAATTAG

TCCCTCCAGCGTGCAGCCCAAGCCCGAGTTTGGTGTGGGATCCACCAACTCGGGCCAGCC

CGAAGGCCAAACCAGCCCCAGGTCCAAATCTCGGTTGCTGACGTCACGATGACCCCAGGT

CAATTCATTATTTCATTAAGGAAAGAGGCAAATAAATTCTAATATTTAATTAGTGAAGCG

TTTTCTTCTTTTCTCTCTCCCCTCTCTCTTTTCTTCCTCTCTCTTCACCAGCCCTTTTCG

TTTCTTCTCTCTCTGTTTCTAGTTTCATTATTTTCAGCTTCTCTCTTTTTGGAACTTCTA

CGAATTGGAACTGCATGCAATTTGACAAAAAATCATTCAATCACAAACAACCCAGAAAGA

GAACAAGGCAGAAAATTACAGAACTTGGGCAGCTCATCTTCAGGCCTTTTTCCATACCTC

TACTCCAAAACGAAATTTGTAAAATGAAAGTGTTGCCACCGAGTTTAGCAAACCAAGCAG

ACACATCCAAGTGTTGCCACCCACACCTCTAGCCACACCTGAAACCCCCCATAATCAAAC

TAATGTACACCAATAATTAACAGAAGAAGATTTGGCATTCCATGGACTTGGTATCGCAAG

ACCTGATTTTGACATGGCCCTTGCAATGGCAGATACAAATATGCCACAATATCTTGGGGC

TAAGACTCAAAATTCAAATTTCATTTAGCTGATGGCTAGAACCAAATACTTACCCAAGAA

ACCTCCTTTCAATCTCTACGGCGATGGCAGAGCCTAAAGCCACCCAAAATATATAGTTGT

CGCTGAAAGGCAATAACCCAATAATATATTTTGGTTCACCTCCTTCTATATCTCTCTGCA

ACTGCAAGCCCTACCTCAATTGTAATGCAAAGCGAGAGACCAGGAAAACTTACCTGAGTT

AGTTGTGAAGCTAGAATTTCATGTGTGTGGGGCCCTCTCACAAAATAGAAACAATAAAAA

ATTTGAACTCACAAGACACACCAAAACATCAAAAATCATATTGTGTATTCATTACAATCA

TCATTGTCCTTGACGAGTTTTCATATTTTGAAATCGTTGCATAACAACCTCGTCATCTAT

ACTATTGGAAATGTCTTTGTCAATATATGCAACGAAGCAATCATTTATACATAAATTGAA

AGATCAATTAAAATCAATAAAAAGAAAAAAAATCAAAATTTTATGAACTAAAAATAAATA

AATAAAGAAAAAATTAAAAAAGAGAACTACTAAAAAAACTTTAATTGAATAAATTAACCT

AATTAAAACTTGATATTGAATAAATTAACCTAATTAAAATTTGATAGTTTAATATGGAGT

TTGAGAGAAAAACTCACAGGCAGTGCATTGAGAAAGTCTGCTTTGCTGCTTGTGTATCTC

CAAGAAAGGAGAAAAATGGGTTAGGATTTTATCAATTTTTGTAAAAGGGTAAATATATAA

TAATAAGAGCCATAAACCTTTGGAAAAAGGAAAGGAAAGTGGAGCTTGGTGGAAAGGAGA

ACAGGATTTGGCTGCTGGCCCACGTGATAAAATAAACATATTAATTAATATAGATTATTC

AATAACAGCAATACATATTACATTTTAAAAGAAAGAAAATAAAGTTGAGTGGGGGCCTGT

GACTGCACTGGTCCGTACTTGCCTCCGCCCCCTGCCTGAGTATAGCTTGGAAGAAGAGCA

TGAGTCGATATTGAACGAAACGAGAAAGAGGCGTTGAAGGTTGTCGGTGTTGGAGGACTA

GCGTGTTTTATTTTGCGAACTCGCTAATTTGTGAAGCAAGTTTCAGCCTCATTAAATTGG

AAGAGCGAACGATGCATTTTTTTACTGTTAGGACTTTACAATCTCATTTAAGTTAGTCCT

CCCTCTTACCAAACATGGTTTTCAAGTGCTATCTAGTCCAGTCCAGTCTAGTAAGACCTA

CCAAACATACCCGTAACATTTACAAGGCTGGATTTAGCATATGTTGTCAATACAGTATGA

GTATATGACCACACCAACTGATGCTCATTTCTATCTTATTAAGAGGACCTTGAGGTATAT

ACAAGGGACATTACAATTTGGTATTCTCTATTCACCTGGTTCTGTTTCTATTAGCGCCTA

CAGCCACGCAGACTGGACATGAGATATCTCAACTCGCAGGTCTACTACAGGTTAGGCCAT

CTCCAGTCATAGGGCTAATTGTAGTACATGGCTAAAAATTTAGCCCTCCAAAATATTATT

TTCTAAAAGAATACTGCAGGGCTAAATGTTTCCATTTTCAGCATGCATAACTATATTTTA

GAGCCCTTCATATTTTATTTTTGTGAGAAAAACTATAGTACAATACTCATATATTCCACA

ACTATATATTGTTTAATTTAATTAAACTACGATTTAGAGACAATATTTTCTAATTTATAT

ATATATATATTTTAAACAAAATGAATTTAAAACATTTAAAAAGGTTTTAAATAAATAAAT

AACTTTTCAATCAATGATTCTGACTTCCAACGATCAGAAACCAATTATATTTATAATTTT

TTTAAACGAATTTAAAAACATTTAAAAAGGTTATTAAAAATAAAAATAAAAATCTAACGG

CTAGCTGACGTCATTAATGATGTCAATGCATCTTGAATATTGGTTGTTGGATCTGCAGTT

GCATGAGATATTTCATGCTCTTTCCCACGCATGATACTATCTCCTCTTGAAAAGCAAATG

CGCTAGGCCCATAAAAAAAACTTTGGCCTAGGCAAAAGCTGGGCTACATTTGGCCTAGTT

GAAGGGCTAAAAGGCCAAATGTGACCCTCCAAATTTGGCCAAAATTTTTTTTAGCCCATG

GCTGGAGATGGATTTTATATTATTTTATTGCTATATTTGGTCTAAGGCCCATCACTGGAG

ATGACCTTATGTAGTATTTATTGGTTCTAATCCAATATCTTGGCAATCACGGAAACAAGG

ATCTGTTTCTCATAGTTCTACAAAAGCAGAGTATCGGGCTCTTGCTAACACTTCTGCTGA

TGTTGCCTGGATAAGACAAATGCTTGTAGATCTTCATGAATTTCTTCCTGGGCCTCCAAT

GATTCATTGTGACAACATGTTTGCTTTAGCCCTCTGTTCTAATCCAGTCTACCACTTCCA

TATTAAGCACCTCATTATTGATTTCTATTTTGTGAGGGAATGGGTTCAACGCAAAGATAT

CTTAGTGCAATATATTCCAACAGATGAGCAGGTTGCAGATGTATTTACAAAGGGACTTTA

TGCTCCTGTGTTCTCAAAGCACTGTACCAATCTCAGACTAGGAAATCCAGACTAAGCTTG

ATGGGGAATGTTTGGCTAATAGCTGACATGGCAAGGCTGCTGATGTGTCAAGACTATTTC

GTTAATCATGGGTGTTAGTTTGTATTAGCTGTTGTAATAGTCACGTGTATCTTTCAGTTA

GTTTGAAAGAGCTAGCTTAATTACTAAGCAAAGCCCAAAGTATAAAGGTCTCTTGGTACT

GTAATATTCATGAATTGGAAAAGAAATCTCATATACTATTCTCATTTTCTCTCCCTGATT

TCTCGATTCTTCTTCTTCTTCTCAATTCTTCCTCAATCGGTAGCAGTCTACACTCTTGCC

AAGAACGGACATGTTTTTACAAATATTTGCATTTGGTTTTGTAACTTTCCTTTTCAAGTA

TTACATAGTTTTAAGTTTGACTTGTACAGTTGTAAGAGCAATTCCAGCGAGCAGCCTTGG

CCCAGACAGGAGGGAGCCCAAGCCCTAGGTTGACGTTCCAACGGGCTTCAACAGCCCTGG

TAGTTGGTAGGCTCCACAAGCTTGGGCAGGCTGGAAAGCAAGAACAGCCTAGGTTTGCTA

ACGTCAGCAACGAAAAAAAATCTTAAAAAATACTAAAAATTTCAGAATTTTTTTTAAATA

AATACTTAGTCATATTCTTCACTTCCCACACCAAAACTCTATACAAATGCCTCTCTTCAT

ATCTCACGTGAATATGGTTGCCCTTGAGTTGCCATGACAATGGGTGGAAACACTACAATC

TTTTTGCAAATGCTATCACTATTCACATAAATAGTAATAGCCCTTGCCCTTTGTCATGAC

AAATGGGTGGAAGTGCTCTAAGATCCCTATAGAATCTTCTATGTAATATTTGTGCTTTGG

TTTACCGCAAATCTCATAGTCTCATAAATAAGCCAAAGACTGTATTAAATTACGAACATG

CCACTATTAGAGGTGCAAAACCCTAATAAACTTGAATGAATTTAAATAACTTATGTGCCA

AGAAACCTGACTTTTGATCAAACAATTGTGCATTGAATGGGGAACTTATTCAATATAGAT

TCTTTTTTATACCTCGAGACAAATGAGGACTTTATTATATCTCTAATACAAAGCTGAGAG

GCAAAGAAAACAGAAAACAAGTAACAATGTGCCAGATCGATCAGATCTTATCAGAACTAG

GAATGCTACCACCTTGACTTCTGTCAGGGTCACTAAATTGACCTAGTCTCATTATTTTTG

CCAATGTACTGAGCCCAAGCCAATTGGCCCAAAATCTTTAATTACTTCCCATGTGCTGAA

AATAGGCCCAAAATCCGGTGAGTTCCAAGGCCCAAAATCCAGAATTTCATTTTCCAAACT

CAACCTCTAATCCAATTTATTAGACAACCTATCTTATGTATTCCTCAATAAAGCTCAAAT

TAACTTTGTATCAAACTAAAAAATCCAAAAAAATCTCATGAACATAAACTCTAAAACAAA

GTTCCACCAATAAAATCATCTCAAAACCTTATCTCAATCCTCAGCTAAGCTCCCAAGGAG

AACAAACAACCAATTCAACACTTGAAAACAAGGAAGAGGTTGTTTTGGTTATACCACTGC

CGAGAGAGGTACAGTGGCTGGATGGCCACGATATTCGGATATGTTGCAGAGAGCACAACT

CTGAGCAACTCTTATGAAGGAAGCCTTGTCATCTGAGCAAGGAAAATCGACATGCAAGAG

TGAATATCGGTACTCTAGAGCATAATGCAAGAAAAGATGACCAGTCTCACCTTGGACACG

ATTTCGGATGTTTATTACAGAAGTCCAATTACGCTGAGTTTTGGATATGTTAGAGTTCTC

GACCTAAGGAACAACTTTTGTGAAGGAAGATTTGTGAAACGAGCACTGGAAACTGACATG

CAAGGCCAAATCCTTGCCTACAGCAGAATACTAGGCAGAGATGAACAGCTTGCACTCACA

GTCAGTTTGAGCTGTACTGCTTCGATTAGCCTGGATTTTTTTATATGTTTTTGTAGACGA

GCAAAGGAACGACTTTGAAGAAGTACTTTTTCTGGTTTGAGCTACAGAGGGTGCAGTTCT

TAGCCTCTGAACAGGTCTAACGTGAAAATGGACAATTGGAGCTCTCAACTTTGAAGAGAA

ATGAAACAATGATAACCTGCCTGGCTTTTTTAGGGCATTCATCCAGTTTTGATCCCTGCT

CAAATTACTCATTTCAGTTTTCTTATTTGTAAACTGGAATTAGTACTTCTTTTTTTTTAT

AAACAATTAAAATTTTTAATTAATTTGTTAGACTTTAATAAATTTTTAATAATAATTTAA

CATAATTGACAAAATTTGTTGACAGAATGAAACACAAAGATCAAATCGGTCAAATTAAAA

ATATGAAAATTAAATTGACCAAATAACCATTTTGATATTTAAGCCTAAATTATAGACGAG

TATATTGTTCCCAACATACAACATTCGGTGTATGTACTTCTTCCAAACACCAATCGGAAT

CAACTATTAAGACATAGTTTCAATTGATTTTTGTATTAAATTAGTAATTGACGACATAAC

AAGAAAAAACTACTCCATTTATGATATAATTATAGGGTAGAGGGTTCATCCTTTTTTGCA

TACGTGTGTTAAGCCAGTTAGTTCACCCCCTTACACTCAAGTTCGAATCCCCCTCCTCCT

AAATTAGATTAATTTAAAGCAACTTACACTATGGTTGTATTGAAAAATTAATAATCAAAG

CTCACCCACTTTGTTTTTGTTTGACTACATTGCATACACCATTTAAAAAGTCCACAATAG

CTTTGCATGGCGCATGGCGCTTGGCTACTAAAAACCTCAACAACAAAAAACCAGGAAATG

TCCCAATCAATCCACGCACCGAAAGTGGATGAAATATTTCTAACATTTTCCTCCTCAAGT

CTCTTTCAACTGTTTCTTTTTCTTGTTTTTTAATTAGTTTCTGGCTCAACTTTGCGGCCA

GGAGCCATTTACTTCATTTTCTAGTGGAGAAAGAAGGCACAAAAATGAGCAAAATTCCAC

CAAACTTGCATCCTAATTTTTCTCTTCTTTTATTTGTTTGGTCACCCTTTTTCTTTTCTC

TTATTTTCTTTTATTTGTTTGGTAACCATTTTTCTTCTCCTTTATAGAGAATACCTGAGT

TAACAGAAAAAGGACCTGATATAAAGGAATGTAATGTATAGTCAGCTTCGTATACATAAA

TAATATCTAAACATCAACATCATATAATATGGATCTTTCAGTTTGAACGGAGTATACGAT

GACAGCACCAAAAACCTAGTAGAAAATGCCAATACAAGCAACAGGAAAAAAACTGGTTCC

AAGTGGCAATCACTTTGTTTTTCTTGGCATAAAAAATGGGTAATGATTTCCCCACTCCCA

TTTTATCCACTTACACTCCTTTTTTAGTTATAAAATGGAGTGAAAGTGGATAAAATGGGA

GTGGGGAAATCACCACTCTAAAAAATAGCCCGCTAGATAAATCTGACCGTTAACGCATTA

GAAGCTCCACATGCATCAATGGTCAGGAAACACCCGGACTCACGTATGACATGCCTCTAA

CATTCGCTAATTTCAGCAAAAGAGAAAAAGAAAAGAGCAACAGAATAGCTAATGTTTGAC

ACTCATTCCTCACACTATATCAGGGTACAAGAAAATTTACTCACACGAACAATGCAACTC

CTATAACAAGAGGCATAAACAATTTAACACGGCATCTGATAAAATTCGACAAGGATTGAG

AGAATCCATATCCAATATCTTTTTCTTTTTCACCATCCACATCCCACAAAATCTCAAATC

TCTCCACCATGATTTGGAATCCACCAAACCCCAGCTACCTTCTCATGTTAATGCTATCCT

AATAAGCCACTAAAACCAAAACCAATAGGAGAGAAATAGAATAAAATTAACATAACAGAA

TAAACGATAAAAGAACAATACGATGTGACAAAAAAAAATCAAAATATAAGTATTAGAATA

CCAATAGCAGTAAAATTGAATGTTACATTACGGAAGGTTTCATAATTTCTCCATCTTCCT

CTTTATCCCTTGGTAACTGGCACTAGCAGTTACCATATTGTAAAATTACATTAGAACACA

TCAGAAGCAACAACCTTTCCAATCCCCAAAAATGGACCAGAGGTCCAGGCCCATATTATG

AAGAAATCTTTAAGAAGATAATAGAATGACCACCCCACCCTAATTTGTACTGAAGTTGAT

AATGAATAAAAGGAACAGGAAACAGCCATAGGATTTTGAGTTCCATATTCATAGAACATG

TTGCTCATCATAGAAGAAAAAATGATGATCATATAAACCTAAAATTTGAAAATATTTGGA

AGCAAAATGACACCTAGACTGGGTTCCATCTTTTTGAGTATAACCCAGAGGTATGCTTAC

TCTTTCTAACTTCTGTAACAATGAATGGTGAAATGAATTTTCTTCAAGTGACTTTCTGCA

ATTGCTTTCATCCATGTTTCTGCTACCTTCAACACCACCCACCAACAAAATCTGGAAAAA

AAGCCATTATGTTATTTACCAAAACCTTCCTGTTAACTACCTTGGGACTAACTGAACATT

GTTTCAGTTCTGCTGAGCCACAAGATCCAGATGCATTTGGTCAATCCATGCTCCCAAAAC

AGCATGATCAACCGATTCCCTTTGGGAGTTTGCATTTGAACCCTCACTGGAAGATCCAGT

GGCTGTAACACCCGAGATTGGTGTTAGCTTTTCTTTGATTTCGGTAGGAGATTCTTTAAC

AAGTGACTGAACCCATGATAGATCTGGCTCCTCTCCATTGTGCAGAAGCTCAAAAGAAGA

TGATCTGCGGAGCTTACCCAATTCATCTGTAGTAACTGCCCAATCTGGTTTTCCATTGGA

GGATCCCCATTTTGACCAACAATTTACAGGGGAACCAACATTGGAAGCTGAGTTGGAGCC

GAGTTCCCGTGAACTAAGGCTACGAAACTGTTGTTGCTTCTCCCGTTGAGCTAGCATGGA

AACCCGAGAGCCCATCGGTGAGATTGGTTCCACATTGCGTGGAGACATCCTCCCAGAGGA

TGGGACCCCGTAAGAGGCCTGCAATAGAGGGTGATCAACACTCTTGGGGGAAAAGTTTGT

ATGGATTGGTGAAAGCATGCTCTGCTGCTGCTGAAATTGATTAAGAACAGCTGATTTGTG

AGTTGGAGAGAAAACACCTGACTGCAATGATTGATCAGAGTACCGGGGTGATAAGCTCTC

AGCAGAGAAGAGATCGTCAAGATTTGAGGGGGTTAGGGTTTTCAACCGGCCAGAACGGTT

AAGTGAATTAGAACTCATAGATGGTTGGGAGAGGCAAGAAAACTCATTTAGGAGCTGTTG

TTGCTGCATATCGAACTCTGGCATTATATCATAATCATTGGCTGGCATATCTCTTGCACA

GAGGGAAGATCTCAAGCGACTAGACTGAAGATTGCTGCCTGGGAGATGCAAAGCTGGGAC

ATTAGGTTGGGGCCAAGCTAGAGATGAGTGTGACATGCCATTGGCAGATGGGGACATGGG

TGGAGTAAATGGCGAAGGAGACATGACATTAACTGATGATGGGGAACCAGGCAAGAGGCT

CATTGCAGCAGCAAAATCCATGGCTGAAGCACCAGAAGTGCTCGAGCGAGGAGAGGGAAC

AGCAGAACCAGTAGAGACATATAATGGCCTGAGTTCCTCTGCAGTGTGGGCAAAAAAGCA

GACCCTCCTTGCACAACTTGTACCATCTTTGCAAAGTCGGGTTCGATACTGAGCTGGGTG

TAGCCAGCACTCAAAAACCCCATGAGCATATTCACACATATCTCCACGTCTACAAGCCCC

CTTCCGGAAATCAGGGCAAGGAACACAGCTATAATGAAACTTCCTAGGATCCCTTCTGCG

TGCATTTTCCCCTGGATGGACAAAAGGGCACTCAGTCCAATCATGGGAGTACGCACGTGA

ACAAGGCCTCACCTTGAATGAATACATCCGAAACTCATCAGTTGAATAAATGCTGTTCTT

GATGTCTGGGAGAGAAGGGTCAACAGGATATTCCTTCTTCTCTGACGCAGAAGAGACAGG

AAGATTGTAGAACTTCGACTTTGTTGGAGAACAAGTGAAATCTAAAGCAGATGGAGACCC

ATTTTCTGGGGAAGCTGAAAGAGGAGGAGAACTTGAATGTAGGGTTCTTGTTGAGACCGT

TAGAATCTGTTCACCAGCAGAACCATTAATCATAAGGAGTTCTTCTAGAGCTAATCTCAC

ATTCTGTAGCCTAGGAGGAACAACTATAACTTCAACAGGACGATGACCATTAGCATCAAC

CAAATTGGGATCAGCACCAGCCGCTAAAAGCAGCCTCACAACATCCACAGCATTCTCAGC

CCCACCTGAGGCAGCACAGTGAAGAGCAGTGCTCCTATCAAGCCCACAGGCGCGATTTAC

ATCAGCATCAGAGAGAGAAAGAATAAGCTTTATCACATCAATGCTGCCATATATAGCGGC

AACCATTAAAGGGGTTCTCTGCTCATTAACCATCTGCTTTGAGCCCTTCTGACGACAATA

CCATAAACCAATCTCATCAATGGCAGAAGGGTCCCGTTCAATCGATCTTTTGAAGCTCTC

AATGTCATTGTTTGCAGCAAGCTCAAGTAAGCTGGCAAAAGTATCTTCAGTTTCAACTGT

CAAGTGATTATTCATGGCTGTGTTGAGGTCTATTTTCGTCAACAGAGATATGAGATGCTT

CTGGAGAGGAAGTGGGTTTTAGCCGCTCTGATCCCTAGCACATTCTTTCATTTGCTGGGA

GGCCAGCTGAAAATAACAATCACATAATTAAACTATGAGTAACCATCAAAGTCATATTTA

CACATACATATATAAGCACTAAAGGTCCTCACCAAAACCATACTAGAATAATCCAAACAA

TAATGAGTTCCACATATGCCTCTTCAGAGACAACACCCACTTACAAATTTTGATTTAGAA

TAAGAGACGAACAAATAACATATTTTGGAAGAAGAAAAACAAGTTTAGTGGATTTATCAG

AAGTAAAAATGTCATAAGGGAACTAAATAATCTAAGAATTAGATTAGCCCATGCTAAGTT

TGGTTTTCGTCAAAAGCAAGAGCTAATGGAAAAAAGTGCGTGATAATGGTTCCCTCTCTT

AAGGACTTTCACTTTCCTACATACACAGTCTCCAAGAAAGCAAACAGAACCAAAAAAAAA

AAAAAAGAGTTGAATATGAGAGACATCATAGATTGTAAAATTGATGGCAATCAAGCGAGA

CACACATAATTACAAATAACAGAAAATGAAGAAGAAAATTCAAGAAATTAATCTCTGGTT

GGATACGGAGAAAATAATTGAAAACAAAAAAGAAATTAAGATTCATAAATCCGGGTTCTT

TTATCAGAAACAATAACATTCAACAACGCTTTGTATAATAACGTTTTGTAATTGAATAAT

ATTTTTGAGAGGAAATGAAAAGCCCACAAAAAACCTGAAGACAAATTGCATCCTTTGCAA

TTTCATTATCACCCCCACTTATTTCCCAGCAATTACACAGAGGTTAAGAAAACTTCAAAT

AGATCTGTGAGCACAAAAACCAAAGTCAAACTCAAAAAGACTGTACTATATCATTCCCAA

ATGAAAACAACATAACGAAAATAAAATCAAAATAAAATTCATAATCAGATCCTGAGGATC

AAATCAGCAACAATCGCAGTAATGATCTTCTTGAAATCCATTCAAAACTAATTTTCCCAA

AAAATGTAAACTTGAGAGAGAGAAAAAATAAATTTATGAGAACTTCTCATTTCCTTAAAC

TGAAAGCTTCAAATAACAAGTAATCGCAGTCGAACATCAAAAGCATACGTACACAACATC

CAGGGAGATAGAGAGAGAGTCTCGCCATTTCCAAGATCAAGAGCAGAAGACAGTACAGAA

AGAGAGCAAGCCCAAGTCTTAAGACAGTCTCAGAGTGAGTATATATACATATATGTCTGT

ATGTATTTGTGTATATAGGCGGTGTATAAGGTAAGCTTTGTGATCGAAGCGAATCTTACC

AAATCTTCCAGCTAAGAAAGCAAGGAAAGCACCATTGAGGCTCTGTGACAGTGACGCAGA

TCTAGAGCTGCTTCTGGTTACTTCTCAAGAAACTGTTTGGACTCCGAGAAAATTTCCTCC

TCTGCTTTCTGAGCACAAGAGAGAGAATGAAAGAAAATATTAAAGCGAGCTTTGTTTCTC

TTCTTCCATCTCTCTCATGGAAATAAAAATTTAAAAAGTATTTTTTTTTTAAATTAAAGA

ATTCTGTGTTCTTCTTTATTTCTTTTTTTCTTTTTTTGGGCTTTGATAAAATCGCCTATC

CAATACCAGCAAAAGAAAATTAAAGAAAGCCAAAGCGCACTGAGAGAGTCACTGTTTGAA

TGCAATTTCTTTAATTTCCACTTATATCGCGCCACGTCAGGCCACTCTGGACATGTCCAC

CATTTTCGGAAAAGATAGGGATATTCTCGGAAACTTGGGTTCTCACGAATCTCTACGTGG

CGGACCCTGATTAGATGATGTTACGTGGGTAAAATGGAATAAAAATACAGGAGCCGAAGC

TCTGTTGTAATAATTTAACCTTTGTCTTTCGGTCAAATGTCATAGAAATTCAACCTAAAT

TCACCCTACATGAAACTTAAATTGTCTCTCCAGAAAATCCCGTGCATCAAGTCATACGCA

AAAACCGTGCAGAACATGACATGGCCTTATCCAAATGGGTAGCTGTCCACTCAATAACCA

ATATATTTCTGACACGTAATCCAGGACGGTGGTTCTGGAAGCAGAGAATAGAGACTCTTA

ATGAATTCTTACACGAATTTTATAAGTCCTTGGAAACCGGTTTGGAAGGTGGAAAGGTTG

GAGGGGTTACCGGTTAGGTGAGTTTTAAGGGGGATTAAGGTGACCGGAGTAGCCGGTGAG

GACGCACGCGCGGGAGCGAAGTGGATTAAACTGGAATGAAATGAGAACTGGCGTCCGCCA

ATTAGGGAGGTTCGGAGCGCTGGTTGCGAGCCTACGGGAGCGGACTTAAGGCACGAGTCA

ATTTTGGTTAATTGTTTAGTCAAATCACAAGGGGGAAGGAAAAAGGCATGGAAGCACCTT

GGTGGCAAAAAAGCATGGAGCACCAACGACACACAAAACATAAAGAAACGAAAAGGCAAA

AACAAAATTAACTATTAATAAAATTATTCTTCACCCTTAAGTAAATTATTTACAAGTTAA

AATAAATTATAATCCTCTAAATTATGTTTATATGAATATGATATAAAGATTAAAAGGGTC

TACGTTTTCAATTTTCTTTCAAACTTACACGCATAAAAAGGAAGGTTTATTTTTTGATAT

ACCATCTTAAACTCATTTTGAATTTCACTTTGCCACCTAAAATTTACTCCATTTTTCACA

TTTATTTGTCTCCCCTCTGTTTTATTTCTCCAATAGTCTGGCTATTGTACCGACATAGAG

GGACAAGGGGCTGCGTGGAAATGCAAATTGAGTTGGGGGGTGGTGCTGCGTGGAAATTGG

GTGGGGAAGAAGGGGTGAGGGATGGGAGAAAGAGAGGAGAGAGGAAGAGGGCAATCATAT

TTTTATTTTATTTTAATTATTTAAAGTTTAAAATATTTTACTTAAATCCACATGTCGTCA

TTTTATTAGTGTGTAGGGGTCCACATATGTTCCACTCAAGTAATTTAACAAAAAATTGGA

TAGAAGTTAACTGCAAAAGGACTCACTAGTGTAGTAGTTTGGAGTATTTACTCCCTTAGG

CAAGGTCCTAAGTTCAAATCCTAGCATTCATGTAGTGTGTGTGAGTTTAGTATGCTATCG

CTCCTCTCAATAGAAAAATGTCCTCCCAAAAAAAAAAAAAGTTAACAGTTGGGACCAAAT

TGATGGACAAAAAGTAACCTAACCTAGACAATTAATGTCACAAAAAAACATAAAGTATGA

AATGTGATAATTTCGCAAATCTCATGGACCATTTGTGATAAAAAGCCAAAAAGAAAATTA

AAAACTTAATAAAATTTGTATGATAAAAAATTTTAATAAAATTTAAAACCGTGTGGCTTC

TTTTATAGAAAAAATGTTTAGTTATTAAATTCCCATCAAATAATACAAAGTATATTTATT

TTGATAAATATTAAAATTGCCATGAAGTGGTGTGGAGCAATTGCTCTCTAGGAAAGTTCA

TGAGTTCAAATCCTAGCATTTGTGTAGTGTGTATAAGTTTAGTTTATTATCGCATATCTC

AATAGAAAATGTTTTCAAAACAAAATTGTCATGAAGATAATCAAATAATTATGAGCCCAT

TTGACTTTGTGTGTGTGTGTTTTTGTTATCTATGTAAAAAATGATGGAAAGTGGATGATG

AGAAAGATAAGGAAGTCATGAAGGAGAAAAACGTGTATGTCAAGAGATAACTCTCTTTTG

CTGTTTTCAATCAGTTACGTTCCACTATGCGAGATAAAGATTTCACATGGTAGAGCGTGA

TTGGATAAGGACAACAAAATAATGTTATCTCCGTGACATGCAAGTTCTTCTTATAATGGA

GGAATGTATGAAACAAAAATTCAAAAAGAACAATAATAATTTTTTGGAGCAAAAAGATCC

TCACAACAACTACCCTTCCTTTAAAATAAGAACTAAGGGCCCGTTTGGTTCACGAATTGA

ACTTTTTGAGCCAAATGGTTGTTTGGTTCAAGGTTTAGGAGTTTGAGAATGGATTTGAAA

TTGCATTCTCATGTTTGGTAAATAAGGGCTCGTCTGGTAACGTTTTTTGAGAATATTTTC

AGAGAATGAGAACGAGAAAACAAATTTGCATTTTTAGGATTCGAGAATTTGTTTGGTAGA

TTAATTTGAAAACAAATTCAAGGAATAAAAACAGTGAAAACGTATATGGTATTACAATTT

GAGAATAATAATATATTATTTTTCAAGTGTAGTTTTGCTAATGTATGTTGAAAAAAAATT

AAATAAAACAATAATAAATTTTTAATGTAAAAAAAACCTAGAATTATCTTTAATATTAAT

TGAAAATGATAAATAAAAAAGTTGAATTTTTTTGGTTAATTGATACACTGAACAAGTATA

CTTCTTCCACCTTCTCTATTTTTTTTTTTAATAATCAAATCGTTTTTATTATTGCTGAAT

GTTTCACTAGTATTATGTTAACAAGATTAAAACTATAAATAAATAAATAAGCATATATAA

TTTGGGGTGTGACAATATCATAGTGTCCTTACTTAGCTTAAAATGAGGGCCTCCTTATAA

ATGACTTGAATTTAAGACATGTTTTAATATTGGGAAGAATATAATTTGAGGTTATAGTTG

TATGATAATGTCCCAATAATATCATGATAATCTCTCACCATGATGAAGCATCCCATGCCT

AAGCACACACCTCCTCATCATGATAAAGGCATGACTCGACTTTTACAAAACATGGAATTA

GGGATATTTGCTCCCCTTCCTTATCTTATAAATACCCTCCACCTACATTCAAACAAGGAT

AATAACTTCTCTTCTCCTAGAAACTCTGTCCATTTGCTTTCTCAAACACTTATTTGCTCA

ACCTCTGACTAAGACTTCAGATAGGGTTGGTTGGTTGGTGATACACTAGTACCTCTTTGG

TGACTTGTACTCCTACTTGCGCGGATCATTTAGGGCAGAAGAAGAATCCCATCTGAATCA

AAACTAAATTGACCACCTAATCCTAACCAGCCAACAAAGAAAAATATATACATAGTGAAA

ATTGAGTTCTTGGGTATAGATACACACCAAAACTTAAGAAAATTTGCGAGGACCACCTTG

GGCATAAGGGTGAACGTGTACCCCTAAATCTAAGGTGGCTTTTCGCATTGAAATTCTCAT

ATTATCAAATTATTAATGGGACAATAATTCATGATAAAAAAAATATTTATCTTATTAATA

ATTCATATCAACAATGCAACAATATAACATACAAGACTTTTAACAAAAGGAATTTTTTTT

TCTTTCTAATATTTGTTCGTAATTATTATTTTCATTAAAAGGGTTCCATGGAAACAGAAA

AACAAATATTCCTTTGAGCCGCCTCACTTGAGTAACAAAGTGAAAGAAATTGAGATAGGG

AAAATATTTGGGGTGTGATATTCGGACCGCAACGTTGCGTCCGAGTATGATCATGGTGCT

GGCGTGGTAGAGGGGATCGGAGTACAGAATTATTTGGATGTCGCATGTGGGGGCAGTATG

CGGGGATTCGCCTTTTTAAGTTCTTAGTACAAACTTATCGGCTTCGCGTGCTTTGGAGGA

AATCTTTCTCGTTGGATAAACAATTTTTTATTTTTTACTATATTTGTTCGGTCAACAAAA

ATTAAATACTAAAAATGAAAGTAAAAAAATGAGCCTATGAGCTAGACCTAGGACTTTATG

TACGTGTCTAAGTCCCTCACAAATTGGAGAAATTATAGAATACTACGGTAGTATAATTAC

GTGATACTATTGTACACGTGTTATAATTTAGATAGACGATATAATTTATTTTCCCTCTTT

TTAGGGAATAGTATTCATAAATATCCCCGTATATTATAACATGTGTATAAAATATACTAC

ATGACCGTACTACCGTAGTATTCTATAATGTCTCCACAAATTGAAACGTATATATTAAAA

AATAATTTACTTATACAACATTTTTCTTATGTCAAACATGGTGCAAACATTTTTCACGAA

AATGAATCTCAATGTTTTATTTTTCATAATTTTTCTCGAATTTATAAATTAAAATGAAAT

TTTTTCTCAACTTTAGATGAAGTAAAGAATATAAATGTCAAAATTTACCTTTATCTAAAA

CTAAATTTTCTCTTTTTCTCGAGACTTTATTTGGTTTATTTTTCTAGTACTATGTTTTAT

TGAAAAATGGAATCCTTTTTCCATTACCCTGTCTCACTTTTTTGTGTGTACAAGGTTTGG

ACCATTATGAAATAATAGTATGTCCCTACAAAACCACTGAAGATATAATACTCTGTTTTT

TTTAGGGGGTTGACCGGGGGATCTAAATAATGACACAATACTTTATATGTAATTAGTCCT

AATTAATATAACATTATGTGGAGATCATTGATTAGAACCATATTTATCTAATATCCTTCT

AATTTAGCTAAATCATGTTTGTAGTCTAGCCTAGTAGAAAACGGCATTGATTTACAGACC

AGTTGTCTCAAGTTCAAACTCCTATGACATCTTAACAGTGTGTTTGAGAAGCCCCCTCCC

CCTTATAGTTTCGACTATCAATTGTATTAAAAAAATCATGTTTGCAATCAATAAGTTAAG

GGTGTATGTCGGTTCAAAACTAATACAATTTAGTCAATCTTCATCCTTGGATTGTGATTT

TTTCAATTGTTTAAATCCAATCCAATCAGGTCCAAATGATATTGGATTGAATGATCGATA

TTAATAATGAAAAAAATACAATAATGAAATATTAAACATTTATAGATATCTTACAAACCC

CTAGCAAATCATCACGTAAAACCCCATTTCAATGAGAATATGAGAAATCCCGAAACCCTA

ATTTGAATTAATGGTTGTGATGATGTTGATTTTTTTAATGATAAAAATTACAAATTCGAT

AATGTTGAAATAAACCCATAAATGGGTCCAAATTGATCTTTAAAAAATCCATGGGCTTTC

CCCAATAAATGATTGAAATTTTGGGTTAATATTTAATTTCCTATTAAAATTTTCAAGTTT

TGTATTTACAATTTCTATCATTATTACTAACATCAAATCTTTTGCTTTTTGATACTCGAT

ATTTCGATTATAGCTAGTTTTTAATACCTCAGTTTCATGATATTGTTAGAGTTATATTAA

GTTTAGCCAAGTAGTCTGAAGCTGGTGTGCTAGCTGTCAGTTAGTTAAGTGAGTTTGTTA

ATTAGCTTAGAGCTTAAGTCATTGACAGCTCATGTATTCTGCAAAACCTAAAAACTCTAT

AAATATTGAGTCAGCTAATATTGTATGGGATCAATAGAGAGAATCAATAAAAAGAAATCC

ATAACCTTTTTAGTTTTCTTCTTCATTTCCTCTATTCATACGTGACTGGTTATCATGGTA

TCAGAGCCTGGTTGCCCACGTGTGAAAGCCCAACGGCCACACGTGCTCCACGTTACCCAA

AATATATTGTCCAGGTGTTAAGTTTGAAAATTCACCACACGTGCGAGGGCATGTGAGAAT

GTGAAGGTAAATAGTCCCACACTGGAAAGTTGAGAAACCTAGCAAGAGCTTATAAGGAGT

TGGGATTTTGTTCCCCTTATCTAAAATTGTTTCAGATACCACATGAGTTACAGTCCTTAC

GGGTTTTTGGGTCTGCTGTGTTTCCATATCTTAGACCTTATAATGGTAACAAGTTGCAGC

CTAGAACTTGTCAGTGCATCTTTCTTGGATATGCTAGCTCCACGTCACCCAAAATGTGTT

GTCCACGTGTTAGGCTTGAAAATCCGTCACACGTGTGGAGGCGTGTGAGAATGTGAAGGT

AAAGAGTCACACATTAGAAAGTTGTGAAACCTAGCAAGGGCTTGTAAGGAGTTGGGCTAC

TCTCCCCATTGCCAATTAGTTTTGGGATGGAACCTCAACTTCCTGCATGATTTATGTTCA

ATATGTGCTTACACAAGAACAAGTGGCTAATATGCTCACCAAGGGGCTTCACAGTCCAGT

CTTTTTGCAACACTGCTCCAATCTAAAGCTGGACTGGCCAGCTTAGCTTGAGGGGGGATG

TTAGAGTTATATTAAGTTTAGCCAAGTAGTTTGAAGCTGGTATGCCAGCTGTTAGTTAGT

TAAGTGAGTTTGTTAATTAGCTTAGAGCTTATTGTATTCTGCAAAACCTAAAAACTCTAT

AAATATTGAGTCATGTTATAAAACTAGAATAATTAGAGTATGAGAAGTACCACTGTAATA

TTGGGAGTGGAATTATATTTCTCTTTGTGGTGTTTACACTGACAAAATTTCTCCTTAGAT

AGACTTCACTCTTTCTCTTCTCTCTGACTCTCACTCTATTCTCTTCTCTCATTCGTGTGT

GTTTACAAGCAAATATAATGCCTATTTATAAGCAAAGCAAATGGCTTTTAAGGGAGACAT

AATGATTTCTCCCCAACATTATTATTTCATCTTTTGACTAATACCCTCTAGCTTTATCTA

TGTGGGCTTCACTTCTTTATAACAAGTCAGCTAATATTGTATGGGATCAATAGAGAGAAT

CAATAAAAAGAAATCCAGAACCTTTTTAGTTTTCTTCTTCGTTTCCTCTATTCATACTTG

ACTGGTTATTAGATACCTCCCAAATTAACTACTTTCTATTTGGATGAAGGAATTAAAAAC

TGCATAAAATTCTAAAATGACATAATTTAGATCTCCATCGTTTACATTTTTGGCAATTGA

TAATTTTAGTGTTTGCATTTATGTATGTTGAATTTTGTAAATGATAGAAATTACAAATTC

GATTGTGAAACGAATTCCCGCTCAATAGCAATTGTGAAATGACATATCTTTTGGAGGAAA

ATAAACTCCATAATCTTGATTCCCAATTTCCACTATTTTTTCCGCTTGTCATTTATTAAG

TTCCGTCCTTATGTTGCCAAACAATAGAAATATCAATTTCTTCTCTTTATTTCCAGACTT

TTAGCTAAATCCCGAATTATTTTCCTTCATCCAAATGGAGGAAAAAGATTTGAAACAAAG

TGAAGAAGTCCTATTAAAAAAAATACTTGTGATTTTATCTCTTCTTTTATCACTACTACT

ATTCTTTTTGGTAGCTAACTTGGAATGAAACTAAGCATATGCATGTGAGGTTTAAGAGGA

TTTTACATTTGGGAAATCTTGTGAGGTTAGGTACAACCTTTATCTTAGATCGAGTTTTAC

CCTTCTCCACCCTTGAATCCAGTTAAAAAAAAGACCCTAAGAATAAGGAGCTTCTAGAGC

ATGAATAAGAACCATAACCTCACAGGGTCTTAGGGTCCCCATCCAGATTTATATTACAGA

TTTCTCCCCCTAAATTATTGTAGTTCTTTCACTTTTCATCCTTAATTTTTTTTTTGTCCC

AAGCTAATCCCTAAGTTTTGAGACCATATTAATGAAGACAATTGTAAGTTACAAAAATTG

ACAAAAGTGGTCACGTTTAAGGGCTAAATTGACAGGATCCAATCCCAATTTCACTTTGGA

GTTTGAGCCGGATCCTGTGCGTGTCCGACACCTGGCGAATGTCGGGCACAAATGACCTTT

TTATCCTTCTTGTTTCAAATTCATTTAAAACTTCCCTTAGACTTCTCCCGAAAATTCGGC

AGAGTCTCCCCTGTAATTTGTCCAAACCCAAAAACTTCCACCTGTTAAACAAACAAATAA

ACTTCACACCAACTGCCATAATAAATCAAATAAACGTTTCTCAACTCTGGTTCTCAGTTC

CAACCAGATATCAGAGCAATTACTACAAATTTATAAAACTTCAAGTACTTTACAACAAAA

TCCTACGTTTGTGGGTGGCGCAGGAGCTAGGAATGGGCCGGTGGTGGATTCCTTCGTACT

TCTACAGCCTGGGGGCGCAAAACAATTTGAAAGTGTGAGTGGACAAAAATAACGTTCTCA

AAACAATTTATAACATAGTAACCCCCGTTGTAAAAATAAATAGGTAGGTAAACCTAAAGT

TCGTCTGTATTTAAATATAAAGCATTCAACTAAACATGTATATGTATACGTATCTATGCA

GATATGAAACATGCACGAATGTCGAGAAAACTCGTATAAAATGACTGAAAACAATAATTA

CATAAAAGCACTGAAATTATTTTAAAACTACCACTTAGGTGTACCCCTGTAAATCCGTCA

ACTCCCCTGGTAAGTCTCGGTGACACACAGTCTATCCGAGCCGCAAACTGGCAGGATACA

AGGGACTATAGTCTGCCTGATCCGCTGGTAGGTTTCGATGACACACAGTCAACTCGAGCC

GCTCTGGCAGGATCCAGGGGACCGTAGTCAGCCTGATCCGCAATCCTAGCAGGTCTCGGG

GACACAAAGTCAGCCGAGCCGCAAATCCTGGCAGGTCTCGGGACACCAAGTCAGCCGAGC

CGCAAATCCTGGCACTCACGGTCCGAGCGTCCCCAAACTCGTGAGGCAATGTCAAGTGCA

CTGACGAAACTGAAAATAGACTGGATGTCCGTAGATATCGGTCCAACTGAGGGTAATCAC

CGAAGTCGAGTACATGGTGGTTTAAAATAAAGTACTTAAGAAAGTCTGATAAATAACTGT

AATCTAAAATTATGCTGCTGTCTTATCTCGTACAAGCCTCAACTCTGTTTTTTACAGCTT

TTTAGCATGTGCAACTAAGCAGCATAAAATTATAGAAATGTTACTGCACAATCATGCTCG

GAAGACATGCAATAAAACTTATTCAAGATCAAATATGAAATTCATTCATAGAAACTCATG

TATAAAATCATTTATATAAACTGATTTATAGAAAATCATTTATGAAAGAAAAGTCCACTC

ACTGCTGGTCTGAGCTAGCTGGACCTTTTAAAGGCCCCTCTTGTGGCTCAGTCTGGCCTC

TGGTGCCTGATTAACCATGAAGCTTAAATTAATAAAACTGCTCAATAAAAGAATTAAATT

AAAAACCCTGGCCCCCGACTCCTAGAAATACATGCACTCATTTTAAAGTCAAGCCTATGC

CCACTTTAGCCTTTCAGACAATTAGAACGGTCTTAAAAGATCCGAAGTTTTACTAAGCGA

TAAACTGCCAGACCGACCGATCCGGAACTTCGTGGGGTCCACGATCTCCGATGACCAATC

TGGGATTCTCTAGAGGTTCCTCACAGAGAAAGACCCATGGCCTGCGAGTTTGGCCCGACT

TGGACGGTCGGATTAGCCAAAATCGCGTTATCGCTTAAAATCCAAACCCTAGCCCCCGGG

TTCGCGATTTCGGAGTATCCGGGACTCCGATTTGCAATCCGTTGGATCCTACACGATCCT

GAAATCATGTAGAACAACATATCCAAAGTTGAGTGCGATCCAATAGCTCGACAACACTGC

ACCCGGGAATCGCACAATATGAAAAATTCGTTCGGGCTTAAACGGACTCCGAATTGAGAT

CCGCGAAATCCTACGCGCTCGTCATAGCACGACGATCGTAAAACTACACAGAGTCACTTC

ACTACCCTCACCCACGCGCCGCCACAGGCGCCGGCAGTTTGGGTGTCCAAAGCGATAAAG

GCTCGCCGGAAAAACTCCAAACCAAGACTCCAAACTCCTATCCTAGGTAAAACACCCCAT

TTGAAGTCACTTTTGTTCTTGGACATACCCCAAAAAGTGGCCGAAAACGGCCGATCACGG

AGGCCGAAGTTCAGCCGATTGTCAATTCGAAAATCGAGTGTTCTACAGCTAAAATCGATC

AAAACCCATCCAACCATCAGTTAGAGCACGAAAAATTGGCGAGAATCCATATCTTACTCG

AACGATTTGGTTGAGAATTGAGGGAGAACGAGAGGTCTGAAATTCGAGTGAAAATCGGTC

AGAAAACTCGATTTTCCGGCCAGTTTCGGGCGGCCCAAGGGTTGCGACGGGTCGGGGAAG

GACGGCGGGGCCGAGGCGGTTCCAACGATACCCACGTCGACGATCGGGAGGGCCTGTGGT

GGTGGCTGCGGCGACGGGAAGGGGAGGGGGCTGCTGGCGCGACGGGAGGGAGGAGAGAGA

TAGAGGAGAGAGAACTGAGGGGAGAGAGAGAGAGAGAGAGAGGGAGAGAGAGAGAGGAAA

AGGGATAAAAATCTGACTTTTTCCAAATTACCGTTTTGCCCTTCACGGTATTTTGACCGT

ATTTTCTTCATCACAACTCCGATTCGAGTCTACTCCTTGTCTACGAACTCGTTTCGTCGT

GCTCTACACAACGGCGTAAGCAGAATTACCAAATTCCTTCTCAGTCAAAAAGTCAACTTT

TTCCCTACTAAATAATGCGAGGGCAAAATCGTCTTTCTTCTAGAATAAATTAATACTAAT

TTTAGGATATTTTGTTTTGGGTAGCTTAAAGGACCTACTAGTGTAGTGGTTTGGAGTAAT

TGCTCCCTCAAGCAGCAAGATCCTGAGTTTAGTATATGATTCCCCATCTCAATAGGAAAT

GTGCTCCCAAAAAAAAAAAAAAAAAAAAAAAAAACCTAAATGCAATGTATATTGGCTTAA

GTTTCGGGCGAAACCACCCCTCATCAACCTATCGGTTTGAACATTCTTTAGAAACAAAAA

CCGATCTGAGGATGGTGTAAACCGTTGACCCAATAAGGTGGAATGTAAAATTGGGTTGTC

TAGTGAGGATGGTGCAAACAGTTGACCCAATAAGGTGGAATGTAAAATTAGGTTGTCTAG

TAATTTTGTTGTTGTTGGTGTTATAATGGAAGGAATCAAGACCTAACATAATCCTAATTC

ATTTCGCCATTAGAACTCACTTGGGCTAACCTTAAGGACTTGGTTGTCTACCTTTTTGGT

ATTTTTAATTATAAAAATCCAATTCTAACTGTCTATTCTTAACTTCTTGAGTCTTAGTAT

TTAATGGATTCTCAATTTTGTCATGACATACTTCCAACTTTTCCTTATAAGCCCATACTA

AATCCCTAACTTTTCAATGTGGGACAAAAATTCTCAACACACTTGAGGTCTTCCAAATCA

TTCCTCCCCTAATATTGATGGAAATTTCAATTGAAAGTTTTATCATATATAACTAATAAC

AATTGTTGAGAAAACATGTAACAATGAAAACAATTTGGGAAAAGGGTTAGAAAATATTGG

CGAAAATAAATGAATTACTGCCAAGGCCCGACTGGTTGCATAAAATATAGTATGCATAAT

TACATTGACCAAATTAATAGTTTATCTTTCTTTTTTTGTTGAGAAATTCTAGGATTGCAT

TCATAATTCAGCTAAAAAAGCCACTAGTCACAAAGGACCAATACAAACTAGCCACAAATG

GCCAATACAAACTATTCACATATGGCCAATATAAACTCGTCACAAAGGGGCCATTATAGT

AAAGGAGCGGTCTAACATCAAAATTATGACAATCTGGTATGTCTCCACTAACAATCACAC

ATGAACAAGGAAACTCTGACATATGCATTCCAACTAAGATTGCAAACTCAGAATAATAAA

AAAGAGATAAAGCTTGAAAAAAACAAAGCCATAACAATACAAAAAAAATTCTCACAAAAG

AGAGGTGAAAAGCTCTCACAAAAGGAAATGTGAGATTTGATATCGAAATGCAGGTGGAGA

TCCGACGCCGAGACGTAACCGCCTGTGATAGTTGAAGGAAAATCATAACAAAACGAAGAC

AAATAGGGAAAAACTTTTGTCTCTGAAAATGAGAGAAGAAGAAAGGGAGGAAGAGAGGAG

AGAGGGAGAATGGAGAGGGGAGAGGGGAGGAAGAACATTGGCTTCCTCCCCCCTCAAAGT

GAAAACGACTCTAGGAGGATTTTTTTGGGAGAAAGGGGGCTAGGGTTGTTTTAGGGCTTA

TTTACATTAGTGCCCCCTGAAACTATGGTCTTATTTTATTTTCTCTGAAAGTTATAGTGG

CCCACTTTACCCTCTTAACCAATATTTTGCATCTTAATTTACCCCCCACCGTCAATTCAG

TCAAAAACTCTGATAAAAATGAGGAAGTGGCACAAATAGGGACCCACAAATTTGTTGAAT

TGGATACCACGTAGGTGCCATGTGAAAAACAAAGATTTTCTTTTAAAAAACTTAACTCAA

TTATTAATAATAATATTTTAAACAAAAAAACAATTCCAATCCCCTTCTCCAGACCATAGT

CTGAAATATCGATAATATCGAGGAAATATCGAGGATATTTCGGTTTTTTCGAATCACGGA

TATTTCGGAACATATCCATGTACATATCGTATAAATATCGACAATATCGACGGTAATATC

GGAAAATATCGATGTCGATAATTTCGCTCACATTTCAGCAATATTTTGTCAAAATATCGG

TGTAATATTGCTAAAATATCGAAAATATCGAAAATATCGATGTGAAGGAAAAAAAAAAGT

TTTTAAAAAAAAAGGAAAAAGGGGAGAAAAAAGCACAGAGGGGATATGAACCCCTCCCAT

TTTACTCCTCCAACACCTTAAACACCATATCACTTATGTTTTTGTGATAATATGCTAAAA

TATTTATATTTATATGGGTGGTATGTTAACAATTACATGCAAAACTATTTTGGGGATTTA

TCATTTGATGACTACTCTTTACAATACACTTATTCTACACATAGAGATGATGAAGATAGT

GAAAATTTTAAACCTCGTAGGAACTCTATGTGGTACTAAGTCACTCATGTATCTTACCAT

GCAATGTATAAAGTGTAAAATATTATAGTAAATCATTATATATAAATGATTACGGTGTAT

TTAATCTTTTTTCATTAATTATTACATATTTTTTACACTCATAGTGTTTCCCCGCTTGCT

GTATAATCAACTTAAAATTAGTTAAATCCATCATGCAATGCATTTCCTTCTAATTTTTTG

TGATAAACTCATAGATAATTGACTAAATAAACATTCTCCAAAGTTTCAATGAAAATTTCC

AAGTTTTTCTTACAATTTCCGTGGTTTTTATTCAATTTTTATCGATATCGATAATATCCC

GATATTTCCATCGAAATTTCCATATTTTTGGACTACCGATATTTCCGATATCATCGATAT

TTTAGACCTTGCTCCAGACCCAGTTTACGCATACCCAACCTCTATGTATCCCTAAATAAG

CCCCCATCATCCTTCCTTCTCCTTCTGCAACCAACAACCACCCCACCATCATACATAACC

ACCAAACCCACCACAAGCCATACCCACCGCAAAAAAGAAAAAAAAGAAAAAAAAGAACAG

CGACTACATTTGTGGCGTGAATGAGTTTGTGGGTTGCAGGGGCAAAAAGCTTGGTAAAGA

AGACAATGGCATGGCCTTGGTATTTAAGGGTTGGATATGGTATTGCGGTTAGATTCAATC

CGTGACAGACCTAGAATGGTGATTGGGTTGAGTCCTCGGTGAATCTGGAGTTTCGGGCTC

GTCCGATTTGAAGAGAACTAGAGAGGTTGTCTTGAGGTGGGGAGACGTTAGGATGGAGGC

CCTCTCCTCTCAGCCGAGCCTATTACACCAGTATCCTCCTCACCATCATCAAAATCACAA

CAATCATGGGCAATATTTTAAGGCAATTCCTTATTACAAAGAGACCACATTTTATAGATA

TGATTTTATATAAACTAGATAAAAATAAAAGGTTGGCTAGGTACGAAGGGTGAAGGAGGA

GATTTTTTAGTGGCGATGGTGGAGAAGACGGTGCTATTAGTTTTTTAAATTTCCTTTTTT

TTTTTTTTTTTTTTGTTTCTCTATATTTTTATGGTTATTTTTCTTTTAGTATTAATAATT

GAGTTATGTTATAAAATAACCTGAGATATGTGCGGATAGTGAGCTGTACGTAAAATAGAT

AAGACACAGCTTTTAACGAGGTTCGGCTATGCCTACGTCCTCGGAGAGCAGCAGCAGTAA

CCTTTCACTATAAAATGATATGGCTACAACTTTAGTGTTTACAACATATGTGGCTCACTG

AAGTTTCTCTTTATGAAAATTTCTCTCTGCTTTCTCTTCTCTCTTTTCTTTCTTTTTTTT

TTCTTCTTTTTCTCTATTTCTCTTTCCGTTTCTCTTCTTATTTATAGGCTGAGAAAATCA

CTATTCATCACTATTCATCACTATTCACCCGTGACAGACAAACTCTATTAAAGCCGCCAA

ATGAACAGTATTAAATAGTAGTGGGCTCCACACCAATACTTTTACAACAAGTTAAGTTTT

TTAAAAGAAAAGAATATTTCTCTTAATTTTTTTACACATGACACCTACGTGGTATCCAAT

TCAACAAACTTGTGGGTCTTATACATGTCATGTCAACATTTTTAACAGAGTTTTTAACTG

ACTACAGTGTAGGGTAAATTGAGATGCAAAACATCGATCAAGGGGGTAAAGTGGGCCACT

TCAACTTTCAAATGGTAAAGTGAGATTTGACCATAGTTTTATAGGGTATTACAATAAATA

ATTTTTTTTAATAATAAATTTATCAGATACACTATTTCATTACATTTTATTATCCAATTG

GTAATTAAGATGTCTCTATGTGCTTCCTCGGTTATGAGATGTGGACGTGAAAGCGACTTT

CACTTGCATTTGTGATTTGTTATAGATAAGAATATGGTCCCGAAATTTACTTTGGAGATG

TGGGTTTTGTAGGGCTAAGATCGCTCCCTTTTAATTTGTCTCTAAATCGGCCAAATCCAT

CAACAAAGAGACCAAACAAAGATGCCCACGCAAAAGAAAAGGACCTGTTAATTTGGTGGG

TAGGCCAAATGCCAAAAATACTCTTAATAAACCCCTCAATTTTTTAGTAGGAATATGACG

TGGAAATTTGGCCGGCTCTATGCTCTATATGTTGGCACCCTATCATGGGGACCAAAGTGG

GTTCTCCATTCCATCATGGATATGACACTTATTTAATCGTCGTTGCATAGACGGTCGAGA

TTCAATTGATGTATTATTCTTTTTTTTTTTTTAATTTTGATTTAAAAAAAAAAAAGGTTT

TGGGTTGGTATATGGAGCAAATTACCACTTTCTAACTAATACAAAATTAGGCCAATGGAG

TTTAGTTTGGTGAAAATGGGCATTAACTTGCAGATCAGTGGTCTTAGATTCGAACACTTT

GATAGTGTGTGTATGGAAAAAAAAAACTCATTCCTTTCCTAACTTTGACAAACTAAAAAC

CAGAAGAAAATAAATAAATACAAATGTGGGTCTCTGCCTGCCCAAAAATGCCAACAGAAA

AAAGAAAAAAGAAAAAAAAAAGAACAGATATACATGGGTCTTGCAGCAGTGTATGGGCCT

GGGCCCAAGCAGAAGCCTGTACCAGATATGACCCATCTAATGTGAATTCATTTGATCAAT

TTTTGTACAGCAGTAAATTCATATCACTAATATGATTAGGTGTTGATGATTCTAGAATGT

GTGTTATTTTCAATTGGTTTTATTTCTCTGGTACTTGTAAATATAATTTATTTAGTAAAA

AAATATAGAAGTTAGCAATGCAAAACTGAACAATTTCAAGACAGAGAAGTTAGCAATGCA

AGTTCTGAAGAAGTGACAGGTTTAGTAAGGTGAAGATTATCAAAATTTAAATGACCGTCT

ATAACTAGCCATGTGTAAGTATGATGTTGGCCAAAGAAAAAGAAAAAGAATGAGTATTAT

GGGTGATTGGTTTAATGGAGAAAAAAATTACTTAATTACGTGACGAGAGAAATAGAAAGA

AAGGATATATGAAACCGTTTCAATTCTCGGTTTAATGAGTTGACTGAGTCAGCTTGTGAT

TTGGCAGAGTTGGTTGGATTGTTGGTTAGTTTGGGAGAAATTGTTAAGACAGTTGGGGTA

CTTAAAAGACAGAATTGTATGAGTAAAAATGCAATGTATATTTTCAGTACACAAGCTTTC

CAGTGAGAGAAGAGACTCAACTAACAAATACTCACACCATTACACACACATATTACCATT

AAGATGCTCCCTGAATGTAGTGACTACTAACAATACATCTCGTCCCTCATCTTCTTGAAG

CTCTGCTGATATTTTCTTCACCATGGCATGCATCGCATACCACACATGTCAAACCAGTCA

AACTGCGGTTTTTTGGATCAGTTGTGGGCTTGGGGTGCGAAGAGAGGGTGATGTGGTCAC

TGTGAGACATTGGAGTAGACTAAATCCTTTTCCCAATGACCGTGCTATTAGAGATTTCTT

AAAAAAATATGGGGTTGAATTTTTGCTAATTGAAGTTTTCTTATTGTAATCGACTTGGGT

CATTTGCTTGTTCTCCCCAATTTCTCCATATGATGAGAAATTATAGTTTTTTTGTTTTTT

TTTGTTTTTTTTCTCATAACCAATCAATATCTAGTTATATGATTAGATTGAACAAGATTA

TCAATTATAGAGCTATGTGTATGAGTTGAACAAGAAACGTCCTCCAAACCAGTCAGAACT

AATAAATTACAAAATTGATATAGAGTATGTAATTCAAGGTTCAAAGCGCCAAAACTAATG

AAAAATCCATCCACATTCAAAACCAGCCATTGTGTTCCAATTGTGACCTCATTCTTCTTC

CTCTTTTCTGTAAAGACTGAATTTTTTTTTTTCCTCCACATGAATTCCTTGTGTTGTACA

TTGCATGAACCACCCTCGTGTAGGTCTTGAAACCCTATCGCTAGCACTACTTTATATAAT

TTAATTGAAAGATTATTGCATCTATAAGCACAATAGAATTCTAGCGTACAATATATATAT

ATTTTTTCCAATAATCATTGTGCAATAAAAATTTGGTTTTTCTTTTTGCAATTGATAAAT

AAGCAAATTTTTAAAGAAATGTTTCGCAATTATTTAAGTAGGCCTCAGAATTATTAATAG

TGAACTACCTACTTTATTTATTTATTGTAAATTGTTACGGTGATACATTTTTCACTCACG

TCCGCAGTTTCTATCTAGCAGCTAAGGAATATATACCCTAATAAAGAAGAAGACATTGGT

TTGAATTATTGAAGATGTACAACTTTGACATCACGGGCTTAGCAGTTGAACAACTCAAAG

CCCTCAGTCTCGCGAAGGGGCCCATTTTGTTATGGTATCTGATGGTCCATTCAAAGCTAT

GGATAGAGTTGAAAATTTGAATGAAATTTTTTCAAGTATTTTCTCAGAATATAAAGTTCA

GTGGGTCCATTGCAATGGAGAATCACAGAGTGGAGGAAAGCTATTTTTATTGCACTGCAT

GCAGCTGTTGCCTTTTTGTCATTCTTGTAGCCTTTTGAGAACAAAGAAAAGCAAGAAGCA

AAAAATGAAGAGAATCTCTCTCTCTTTTTTTAAAAAAATATTTTGGTTATGGCCTTGGAG

ATTATCTTCTTGTTCTTTTAAACTGATCGATTTCAGATGTGGGTGGGTGAGTCTGTGACT

TCTGTGTGAGTTGTCTATTATGTTAGTCATGTAAAGCTTGACTTCATGGAAGGACAGCTG

GGCCTCCTCCTCCAATGTTTTTGTCGTGGTTTTATGATTTTCGTCATAATTAATAGGATT

TCGGATCAACTTCTCTGCTCTCGGTTCTTGGATCCTATGCTTTTTCTACACTTTGCTTGC

TGTGTTTTTTAATCAATTGCATCATTACATAGGAGATATTTCACCTTCCTCATATCCCAA

CTTTTAACCTTTTTTTTTATTTTCTACGTCTTGAATCATTTCTTACGTTAGTTGATTAGG

ATAGTGAGTCAGTCTCTTACATCCGAGTTCGAATCTCTTTTCTTATAAATTAGATTAATT

TATAGTAATTTAGACTATCGTTTGTTATTAAAAAAATGAAATACATTAAATCAGTGTTTT

TTCCTTGTTAATTAGGTAATCGTTGTAGGGTTTGGGTGACAGAATGAGAATATGAGAGGC

ATTAAAGTGGGATGAAGAAGTGGGGTGTACAACGATGGGATGAGATACATTTTGGGGGGA

AACTTTATATTTCATTTTTTAATCTTTTCTAAAAATAGAAAAGACGCAAATTGATTAAAT

TTAAATATTCCAAAAAAAAAAATATATGTTCATAAAATTAACTAAACAATGCAACTTGGT

TGAAAAAGGAAAGCGCGGTAACATAGCAATCTGTGAAATTAAAAGATATCACGTTTTCTC

AATTCATTCCTTCTTAGTTCTCCCCCGTTCAGCAAGTCATAAGAAAATTAAAAGCTTTCT

TATGGTTCTTCTTTAAAAAAAAAAAAAAAATCTTATAGTTATACTCATTTCGCTAGGTTT

TCGTATCAGGGTTTTTGGACACACAATTTCAAAAGTGTAAAGCAATTTGACATATCGGTT

GGATCTACTCTAAAGCAATAGTCATTCTGCTAAAAAGGTATGTTCTTTAATTTCTTCATC

AATTTTCGTTGGGTTTTGAGTTTGTTATTGAAGTCGATGAATAATGATTTTGTGTTTTTG

TTTTTGTAATATTGTGTCATTTGATCACTTAGGCGAGATGAGGAGGGTACATAATTTACT

TTTGTTCATAAGTGGATTATGCGTTGTCTTCATTTTTGATGTAACCATTTTCTTTTTGTG

CTTTTCCTTGTTGACGTTTGCTGGCTGCACTCATCATGAATATGAAAGGACAAATTATAA

TCATTTGTAGATTTGATTAGAAATTTTCCTCTTCCTAAGAGACTATGCCTTTTTCTACTG

TGCATTTCTGATATCATTACAAATTTAAGATTTTGGGGTTAATGAATCATGATGATGGAG

ATATCTGTTCATGAATACGAGAAATTCTTCAATTCGGTAGCCAGCCACCTGGACTGTGCG

ACCATGCTCACAAAAGATTAGAGCTCCCATGTATTTTGGTCATACCCAGCCTCACGGTTG

GTCCATATAATGTGAGTGTGTTGCCGGGCCCGTATACGACAGCCGCACGTAAGAATCTCT

CCCATAAAGACATATGAAAATTAATAAAATAAAAAATGATTCATTGGAATGCAAAATATT

AGAAGTGTGCAGAATATTTTAAAGAAAAGATAGGCTGTAACTTTTTACAATGGCTAAAAT

AGTTGGATTCACGTGTGGAATATACTGGGTGCTTTTCCCTCATTTCTTTTTATCTCCCAT

AGCAATTAAAAAAAAAAAAAAAGAAAAAAGAAAAAAGAAGAAGAGAGAATTGGGTGCTTT

TCTTTTCTATAAAATATTATGTTGACCTAAGGAAATAACCGAGCCTGGTCTACCCAATAC

AAACATGACATGTGGCTATATCCATCTACGTAGGGCCGGGCCTTATAGTAAATCTCGAAG

AGCCAACCACGAGGGGCGCTTTAGTCATACAAATGACAAATCATCTGCTTGTGGTTCAAC

CACTAAACTCCTAGGATTTAACTAAAATACTAGCAATCCATTGACCTTATGACTCGAACA

AAGGCGAGTCGACTACTAGTGACCGTCAGTCATCACGATTCCCTTGCGTGCCCGTTGCAA

AAATTTTCAACTGATACGTTAGCTCGAGAACTTAATCGAGAATGAATACTTATCCTATGC

TTAGTCATGTGAGAGCCACGTCAATTATAAGACCCGTCTTAAAGAAGGAGTACATGCCCT

TTCAGAGCCGTCGCCTTAGACACCTCTGATTCCACGTATAAGGTGGCTATTCGCTCCTAG

GTGTGATATGACATGAGTTAGAGGCTACTATGTGCATCACCCTTATAAACATCATAAGGT

ACTCATAACTACTAAGTGACAGCATCCCATTGGTCCTCAGTTCATGCACAACGAGCAATC

AAGATTGACGTAATGTGACAGGAGTTAGAGGCTACTCTGTGCATTGCTCCTATAAATTGT

GGAAGCGATTTTCGTCCTACGAGAATATTCGTCTAAGATTCCGTCTTCCCCTTCGTCGTC

TCTTTCTCCCTACAAAATAAATCGGAATAAGAGGAGCATACCCGGGGTATGTTGGCCAAA

GACCTTCTGATGCCTAAGTTAGTTCAAGTGTTTGTAGGAAAACAATAGCTAACGGAAGGG

GTATGGAATTGTATGTATAGTGTGAATCAGGGCAGCCGAAGACGTGTGTGGTGGCTGAAG

CCTTGTGTGAGAGAGAGAAAGAGTATGACAGCTAGGGTTTGTGAAAGATAATTTATGTAG

AGTTTCAGAGTGGAATTTAGACGTACCTCAACCCTTGTGCGTAGCCATCTATTTATAGGG

GTCTCGAAGGCTAAGGTTTCAGAGGAATAATTCCGTTTATGGAAAATAATTATTCTTCCA

TGATTTGGCGAGATTGGCTTAAATCAAATCCCTAATTAAGGTAGGAATAAAATCAATCAC

AATTAAATCAAGTAACTCCTAATTAAATTGAGTAACTCTCAATTAGGTCAAATAATCTCC

AAATAGAAGGAAATATTTGACCTAGAGTTTTGCTAGAATTTGGTTCCCACATAAACAACA

TAAGATATTCCTAACGGCTAAGTAACTGCATCTAATTGGTCCTCAATTTAGACACAACGA

GTGGTCAGAACTGACGTAATCGACTTAATAAATCGACAGCTCTAGAAATATCCCGTGTAT

CTCTATAAATAGAAGGTAAAAGGGCACCGGCTCCTTAATAGAAAGATAGCTCAATTCATA

TGCAATACACTTGTAAACTTATTAGCCAATATCAACCAAAGATTACTTTCCCAATCCTTC

TAGTGCTCTTCCTCTCCGTTGTCACTCGAGCCGTCTTAACCTTCGCATCTCACTAATAGT

AGGTGGTCAAAGTCCTTACGTTGACCACTGAATTTTCACTGGTTAACATTTTCTTTATTA

TTTTTTGGGAATATTCTTTATTTGAATGTAGCCAAGTAAAGCATGTGTAACTTTTTACTC

TTTTTTTTTGGCCATGCGTAAATGTTTGTTTACTTTTTTAAAAAAGAAAAAGAAACAATT

AATCCTGAAAACCCTTTTAGTATCCAAAATCTTTGCTTTCACTCACTGCCATACAATTCT

CCTCGTCTTTCTTTACCAATATGGTTACTCTGCCTCTTCTCCGGCGAATATTTGTCACCA

TCTCCGGCTACATCAACGCCCCACCTTCCAATTTCTCACTTTTCTTCAGTAGAATGCTTT

CAGTTTAAGCAGCTTCATCCCAATCCTTCTCCTTTTCAACCCATTGCCTACTCTCAGTCT

GCCCCACTTCTTCTCTCTTATCCACACAGCTTTTGGCCCTTGGTTTTCATTTCTTCAACT

GTTCAAGAAGAACCCATTGTCTGTTTTCAGTGTAAAAGTTTCTGTCTTTGGGTCCTCTAG

GAAAAGTAGCCCATGAAAAGCTTTCCAGGAAAAGAAGTAAAATCCTTCTTCAAGCAAAAT

TTTGAATTTTTTTAGGAGGCAGTCATCCTGGAATGATTCTACTTGGAGATAAGCTGGTTA

GTAAATGTTTAAAGTTTCATTTTTTTCTTTTCCAAATCTCACTCAGTCATAGTATAAGTG

AGAAACAAACCCATTTCATATTTGTTTTAGTTGCTGCCAATCTATGGTTTTAATCCATAG

AAGAAGCTTGTTCCAGAAATGGGGTTTTCTCAATTTCTTGTATTTTTTGGGTGCAGATTT

CTGAATTTCTTGTATAGTTTGTGGTTTTTTAGATTTCCTACATTTATCATATTTTTAATA

TTCAGAGATGATGTATCTGCAGCATCATTGTCCAGAGTCTTTAACCTTTTAAGCCCTGAA

AGTGATTGCCTTGCCTAATTTGGGTCCCATTTATCCAGGAATCAATTCAACGCCCACTTT

ATAATCCCCTAAAATTCCAATTTTTTACTCCTTTACAGCTCATTTTTTGTCACTTCGTTC

TCCTGCCTCAATCACCTAGTCAGTCTCTGTTTGATTCTACAAATATTGACTGCTCGCATG

TCAATCTTCTTATGGCCCTTAGAAATAATAATGGGATGTTTCTGAGATGGTCATGGTGGC

AATCCACACCGGTTTGTTGAATTTCTGGGATTTAGACTGTTTTTTCAGGTACTGAATTCT

GAATATTCTGTTCTTTCTGGAATTGTAGGAAATTCATTTTGGTTGTTGTTTCTTTGAGCT

TATGATAGAAAGTTTTGAACTTCAATCTTCATTTGGCCTAGAAACATTGAAATCTATCCA

ATGCCTACGATTTTCTTTTTCTTTCTCTGTCTCCGAGTTGTCTTTATTCCTGCCATTTTA

ACTTTGCAGTGGGCTTAGTCTTGAAAAGGTTGGAACCAGCCAACCTTTTTTTTTTTCTTC

ACATGCTTCTATATTTCTACTCTTAAATTTCCTAGTGATTCAGTACTTAATGGCAGGCAT

TGTTAGGACTAAAATTCCGTTACTTTAGCAATTGTATCTTACCTTATTTGTATTACACTC

CACCTTTCGTGGGTCGACCCCAATTATAGCCGAATATAGTTTATTGTAATCTCCATATAT

ACTTGTGTCCTTTACAGCAAATTACTCAAAGGAAAATGTTCATATATCTTTTCCCACTTT

GATTCAAAGGCAAAGTACTCATTTATTGTTTTTTGGTAGGATCTATAAGCATATTCAGAT

ATGCCTGAACAGGACAGACATCTCCTCAAATTGTGTTTCTTGCTATGTATCTTTGCTGGG

TTCCTGCTACATTCTCTTGGCGCTTCAGAGATTCCCTTGGATTCAAAACTTTCTATAGTT

GATAAAGACATGTGGGTCTCTCCAAATGGTGATTTTGCATTTGGATTTTTTAACAGTCCA

GATGAGCCAAACTATAGTGTTGGGATCCGTTCCAATTCAAAGTCTATTCCGCTTGATAAA

CAAATTGTGGTGTGGATTGCTGGAGCTGATCTTATTCTTGGTAACAATTCTTATGTCCAA

CTGACCCAGGATGGTGAACTAATTTTATTCGATTCCTTGAAGGGAGTGATATGGAGCAGT

AAAACAAGGCAGTTGTCTGTTGTTTCAGCTGCTCTGAATGACAATGGAAATCTTGTCCTA

TTGAATAAAGAGAAACATATTGTTTGGCAAAGTTTTGATACACCTTCTGACACACTTCTT

CCTGGACAGAACTTCTCTATGTTTCAAACACTCCGGGCTGCAAGCAAGAGTTCTGTGTCC

AGTTACTACACTCTTTTCATGAATGCTTCTGGTCAGTTGCAACTGCGATGGGAAAGTCAT

GTCATCTATTGGACAAGTGGAAGCCCTTCTAGTTCGAACCTCAGTGCTTTCCTCACCTCT

GATGGAGCCCTACAACTCCGCGACCAGAACTTGAAACCTGTTTGGTCACTGTTTGGAGAA

GATCACAATGACTCTGTTAGTTACAGGTTTCTTAGGCTAGATGTTGATGGTAATCTCCGG

TTATACTCATGGGTAGAGCCTTCAAAGTCATGGAGACCAGTCTGGCAGGCTGTTGAGAAC

CAGTGCAATGTCTTTGCAACCTGTGGCCAACATGGCATCTGTGTCTTTACTGAATCTGGG

TCCCCTGATTGCGAATGCCCGTTTAAGCATACAAATGAATCCATTTCCAGATGTTTGATT

CCAAATCATCCGTGTGACTCTGGTCCTGACATGCTTAAATATATGCATACTTTCCTGTAT

GGAATGTATCCACCAACTGATGATTTAGTTGCCAAAGTTAGTTTACAGGAATGTAAGAGT

TTGTGCCTGAATGACCCATCTTGTACAGCTGCAACCTTCTCAAATGATGGAACTGCACGG

TGCTTAATGAAGAGAACGCAGTATGTTACTGGCTATTCAGACCCTTCACTAAGTTCAGTA

TCTTTTGTGAAGATGTGTGCATATCCATTAGCTGTAAATCCCAATCATGTGACGACCTCC

CCTTCTCCACTCGAGCAGTCTCATAAGTTTTGTTTCCCTTGTGTAATCGGAGTAGCCTCA

GGAATGTTCGTTGTCTTTGTTTTAGTTCAATTGGCACTTGGTTTCTGGTTCTTCAGAAGA

AGAAATTTGGATAGAAAGAAAGCCGCTTTTGCTTATACCAGCCCCAACTCAAATGGTTTG

ATTGTGTTATCCTTCTCGGAACTTGAGGAGCTTACAGAGAACTTTAAGCATCAGATTGGG

CCAAAGATGTTCAAAGGTGTTCTTCCAAATAAAAAGCCAGTTGCAATCAAAGATCTGAAC

ATAACCATAGAAGAAAGAAAATACCGGAGTGCAGTTTCAAAGATAGGAAGCATTCATCAC

AAAAACCTTGTGAAACTGCAGGGCTACTGTTGTGAGTTAGATCACAGATTTCTAGTCTAT

GAATATGCCAAGAATGGTTCTGTGGAGAAATATATAGAAGATCTTAAATTGTGTAAGAAG

CTGACTTGGGGAAAGAGATTTGATATATGTTTAAGCGTGGCAAGGGCTATTTGTTATCTA

CACACAAGCTGTAGGGAATTTATGAGCCATGGAAACTTGAAATGCGAGAATGTGGTATTG

GAGGAAAACTTAGAGGCCAAGGTGACTGAATTTGGACTCGGGAAAGTAGTCAGCGAGGCA

TCGTGCTCTTCTGCGGAGAGGGATGTAGAGGATTTTGGCAAGATGGTGTTAGTATTGGTA

AGTGGGTGCAGAGGAGTTGGGGACCTTTGTGAGTGGGCATACAAAGAGTGGATGGAAGGG

CGTCCAGAGAATGTAGTAGATAAAAGAATAAGTGGTGGGTTTAATCTGCAAGAGCTGGAA

CGTTCTTTAAGAATTGCATTTTGGTGTCTCCAAATTGATGAACGCCGAAGACCTTCAATG

AGAGAGGTGGTTAAGGTGTTGGAGGGCACATTGAGTGTTGATCCACCTCCGCCCCCATTT

GGTTGTAATGGGCCACTTGAGGAAGAAGAGGAGCCATAGAATCCAGCTATATCGAAAAAA

TCGTTATTTTTTTTTTTTTCGAAATTCCTTCAAGAGTTTCAAGTTGTGTATAGTAGGACT

AACAACAGAATGACAATTTGATCTTGTGTTTTCCTTTTTCTTTGAGAGATAGGCCTAACA

AAAGATTTCCTCCCTATCTGGGTTTCCACCATAATGAAATGTCATTTTGTCATATAATGT

TTGAATTTCATGTTTAATTTTGAAATATGATTTACATTGTAGTCGTATATGCTGTCTTCC

AAGGACAAAGGAGCGAAATTGGGCTTTTCTCAGGTATGAATTATCATTTGGTTTCCACAA

TTACACGGCCTATGGCTATGGGGTTTTTGTATTCTGGTTATCACAGGTGGTGTTTTTCTT

GTCAATGAAGGTTATTATTGTAGTTGGCTTGCATTTTTAAGCCCAACATTTTCTGTATTT

TTTTTCCTTTATTATTTTACTTGTTCAGTATCGAGATGCAGAGAGGCTTCTGATTCAGTT

CAGCTGGTTTTTATCGAAGCTAGTGGGAAAATGAATTAATTAAAGATATACCGATAAAAT

TGTCTGAGGGAACCTTGCCATTGTTAAATCTTCCAGTAATCAAATGCTTCCTTCCCCATA

TGGACTCGGAGCGAAATTGCTTGGAACAAGCATGGTAAACTCAATGATATTGATGAATTC

AGAGTCGGTTTGACATTGATGTGCTGTGAAAATAATCACTGTCAGTATAAATTTAATATA

GTGTACACCATTTATCTTCAAGTCGTGCTAAAGATTCAAGTCTTCTAGATCTGTGTTTAA

TAAATAATGTATGAGTTCATGTTCCACACTATCTTTTTATACTTGAAAATCAAACAAGAA

TAAAAGATTCCATTTACGAGAGAAAATAAAAAAGTTCATATAAAAGTAAACAAATCGTTT

TGTCATCGTATGATTATTGAGAGTTACATGTGGGACTCTGCAAGATAATAACGGGGTCGT

TTGATTAAAAGAAAAACTAATTAATCTTGACTTAGACTGATTAATTAGGTTGAGCTGCGG

AATATTTCGCTACCAAGCTAGTTGGTTTTGCAAACACTTTTAAGTAACATCTTGCCAAAG

CTTCCATTACCAGCTAACAGGTTGGCTACTGATCTAGTCTCTGCAGTGGCTGATCCCATG

ACCACCCAAACCTCCTCGGCTTTGCCTCCTCCTTCGACCCCTCCACTGAAATATGAGGTG

TTCCTGAGTTTTAGAGGTCTGGACACTCGTAAAGGTTTTACTGACTATTTGTACAACACC

TTAATGCAGAAAGGAATCCACATTTTCAGAGATGATGAACAACTCGACAGTGGAGAACCC

ATTTCGACACTCTTGAAAGCAATTGAAGAATCGCAAATTTCAGTCGTCATTCTTTCAAAA

AACTATGCAACCTCCACATGGTGTCTGGATGAACTTGCCACCATGGTTGAACTTGCAGCA

AACACCAAGTCCAGGCTGATTTTACCTGTCTTCTACGACGTGACGCCATCCGAAGTACGA

GAACAAACCGGAGAACATTTTAAAGAGGTGTTTGCTCAACATGATAAAGATTTCAAAGGC

GAGCCAGGAAAGGTGACAAGGTGGAAGGAATCTCTCACTGAAATAGCCAACCTCTCAGGA

TTTGATGTAAGAGGTTTGAGGTAAGGCTGGTGCTTTTATTACGAATTTTCCTTTTTGGGG

GTAAAAATCTCTCAAAAGCTATAGAAGAAAACGTGAGAGTTACAAAACAAACCTCCTGAA

TAAACAGATAGATCAAATCCAAAGAAACTGAAGTGTGTGTTTTTTTTGCTAGAGAACTGA

ATGTGTTGTTGTAAGCTGTTAGTGTGTATACCTTACATAAATTCGATATGTGCTAGAAGT

ACATAAAAATCTGCGACACTCTATGGAAAAAGTCATAGAGATTCCCTTTAAACTTACTGT

TACAAGTTAATGCTCATTTGTGAGAAGGTTCTACACAATCTCATGAAGAAAATATCCTAG

AAGGATATTGAGAATTCTAAATGGAAAATCGGTGAATAAATCCCATGTCCTAGACAAGTC

GTACTAATGTATTGTACTAGAATAATATAGTATTCTAGAGTAGTGTATCCAATTGTGGCT

GTTGTTAAACAGTAGGAGGCGTGGAACAAGTCAGCAACAATGCCTAAAAATAGGTATGCA

AGCAACAGTGAAAGGTGGATTAGCCAAAAGAACTCCCATTGTTACCAAGGATGCCAAGAG

CTAACCCACCAATCTATTAGGTTGCCAATAGCCACATAGAGGCCAATTAGCCTAATTCTA

TCAAGCAAAGAGTTGAGTAGCCAATAGCTACATCAGTAAAGCCAATCAACTAGTCCTAAA

TCAACCCACCAATTAGCCAAGACCCACACAACTAGAAGGCCAAGCCATGTACTATTCTCC

TCCAGTCTAATAAAACACTTTGTTTCTAATTGTCCTACTATAAAATTATGAAATTATGTA

GAAGGAAGAGGTTCCACCCCAAAACCAATTGGCGATGGGGGGAGTTGAACAATACATATA

TTGGGTGATGTAGAGCATGTGTGGCCATTTAGGGCTCGTCAATGGGTCTGGCCCAAATTC

AATGGGCTTGGGCCGGGCTGGGCCGAGTTTTAAAGAGGAGAGAGAAAATATCTGGCCGGG

CTAGGTTTTTTTAGAAAATTCAAAGCCCAAGCCCGACCCATGAGACGGGCTTGAGAAAGC

CCATTGGGGCTGGGCCGGGCCTAAACGGGCCTCACCTCATTAAAAAAAATCATAAACTTA

ATCATAAAGGCATTTTAGCCCAAATTTGAGATTAAACTCACTTAATTCCAATATTTTCAC

TTCAAACCAAATTCATAAAACACTCAATAAAGTCACACAACTTATAAGATTTTCCACAAA

AATAATAAAACCAAGTATTATTTTTTAGGTTATTACATAATTAGATCGTAATACGTTTTA

AGAAATAATTTTAAAAAATAATAAGTATACAAAAATATTTTTTTAAAGGATGGTATGAAT

AAATATGCAAATATTTGGTGATGTGTTCAAGAAAAGCATGATTATATTTGGTGGTACGAT

TATGTTTCGTGATATGATTATATTTGGTGATGTGATATGTTGTTCATCTTATATATATAA

TTGTTGGATTAATAATTGACACAAATTAATGTGCTAATCATCCAATTATAAACTAGGAAT

GAGTAAAGAAAAGTAAATGAAATGAAACAAATTATATATGTGATTTAAGTGATATAATCC

TAAACCTAAACTCTTATTTATACATAATTATATATGTATGCGGGCCTAACGGGCCGGGCT

TTTGTGGGGAGGCCCGGCTGGGCTTCTGTGGGGAGGCCGGACCGGGCTTTTGTGGGCAGG

TCGGGCCAGGCTTTTGTGGGCAGGCCGGGCCGAGCTTTCTTGGGCTAGGCCTTATGGGCC

TGGTCGGGCGGGCCCCCATTGGCCTATGACTCCGCGGGCCAAATGATGAGGCCTTGGGCT

TCACAAGTGGGCAAATGTACAGCTCTGATACAGTGAAGAAATACATTCCCTTGATTGTAT

TCGTTGTTGAGTGTGCAGTATATATAGCATACATGAGTCGTTAGCTAAACAAAAGCCAAC

ATGATCTAGGAGTCTACAAAGTGATATACAATATGAAATAAATTGTGAAGATTATCGAAA

GTAGTTAGAGAGGCAACATACTTGGACTCTTGAAGAATGCTAACAGTATATCACCTCAAG

AAAAACTTTAAAATAGTCCATATAAATCTAATCGAATAGCCTTTCTCAAAGTACATGAAG

AGAACGGTGCATATTGAGAGGTAGGAAACCTACTGTAGTATGACTAGAAAACGATAAGTT

CACATTTTTTCCTTCTTTAATTATGATGTTTACGCACCCAAATACTTAACATAATGCTCT

AACCTTTTTGCAGATATGCGAGAGAAGTGATCACAAAGATAGTTGAACGTATTTTTGCTG

AATTGAATAACACATATCTCACATTCTCAAATGATTTAAAGGACTTTATTGGTATGGATC

GTGTAAATGAAATTAAAGCCAAAATGAGTTAGTGTATAGGGTCTGTTGGAGCAGGATTTT

TTTTCAAGAACACAGGTTCTTGATTGAAGTCCGCATGCTTCCTTGGTTGAAAGAGAACTC

CGAATTACGTCCCCGAGGTCGAACGGTGGAAATGGAGTGAGTACCTGTAAAAGTCACTCC

GACACTCAAGTCAGTGTTTGTGCAGCATACTCTTAGTGTTCAATTGAATTTCTCGTACCT

TTGCTGGAGGTTTAGCCAGGTTTTTATAGCGCTTCGACTTCTTGGGTTTAGCCCAACTTC

CTTAGCTTTGTGCATCCCGTCCTCCACTTCATGGTAATGGGATGAGCGTGTCTCTCTCGC

TCCACAATTTTCTAGTGGGAGGGAAGTTCGATCATCCCACGTATTCTAGTTGTGGCATAT

GGGTTCACGTATTCTAGTTGTGGCATATGGGTTAAGTTATGATACGTGAGATTTGGTCCC

CACAGTGCCCCCCTTTTTTCCTTTCTACACACTTCCCAGGTGGAGAGGGACAAAATAATC

TAAGTGGGCCTTTGGAACAAGATTTCTTCGGATTGAGCGCATGTCATCATGGTGGGCTCA

TGGCTGAGCCCAAACAAACCATATGTTTTCCTTCTTGGGCTTGCTGGTCAAACCCAGAAA

TATGATTCCTATTGGCCTTTCACCAACCCATGCACGTTGCTTTCACACCTTGCGTGTGAA

ATATCCAAACATGAATTTTCATGCACGTTGCTAGCTTTTCAAGTTCCAACTGTGGACTGT

TATCCTTATGTGGACTATCCAAGTGCAGACAACCAATCCCGTGTACAAGTGGACCCTTTT

CTTTATTTTGGATTTCTGCACCTCTGCCGATCCGGTGGGCCCATGCGCATTTTCCTCTTT

GCTAACCGACCCCAGTTGGAGTGCGCTTGGCTTTAAGCTTTTGCCGATCCGGTGGGCCCC

ATGATCCTTGTTTTCTCTTTCCTAGCCAATTCCAAATGGGTTGGCGTGAGCCTGGTTCTA

AGCTTTGTCGATCCGGTAGGTCCCATGACTCTTGGTTTCTCTTTGATAGCCGATCCTAAC

CCCAGTTAGGGTGTGCTTGGGTTTGAGCTTTTGCTAATTTGGTGGGTCCCATGTCTCTTG

ATTTCTCTTTGCTAGCCGATCCCAAGCTTTGAGCTTTTACCAATCCAGTGAGTCCCATGC

CTCTTGGTTTCTCTTTGCTCTAAGATGTTCACGATTTTTTTTTCTTCTTTCTTTGCGCTT

GAGCTATCCATGTGGGCCCCAATATCCGTTCGCTGTCCATCCGGAGCCGTGCGCCTTTGG

TCTCTCAACCGATTCGAGGTGTTCACAATTTTTTTTTTCTTTCTTTGAGCTTGAGCTATC

CATGCGGGCCCAATACCCCTTAGTTGTCCATCCGGAGCCGTGCGCCTTTGGTCTCTCAAT

CGATCCAAGATGTTCACGACTTTTTTTTGTTGCTAGCCGATCCGAACCCTAGTTGGGGTG

CGCTTGGGTTTGAGCTTTTGCCAATCCGATGGGTCCTATGCCTATTAGTTTCTTTTTGCT

AGCCGATCCCAAGCTTTGAGCTTTTACCAATCTAGTGGGTCCCATGCCTCTTGGTTTCTC

TTTGCTCCAAGGTGTTCACGACCTTTCTTTTTCCTTTTCTTTCTTTCTTTCTTTCTTTGC

GCTTGAGCTATCCATGCGGGCCCCAATACCCCTTAGCTGTCCATCTGGAGCCGTGCACCT

TTGGTCTCTCAATCGATCTAAGGTGTTCATGACTTTTTTTTTTTGTTTTTGTTTTTGTTT

TTGTTTTTTTTTTGTTTCTTTGCACTTGAGCGTCCGTGCTGGCCCCAATACCCCTTAGCT

GTCCATTCGGAGCCCTGCACATTTGGTCTCTCAACTGATCCAAGGTGTTCATGACTTGTT

TTTTTTTCCTTCTTCTTTTTGTGTGTGTAACTTCTAGACTCCATATGCCTTCAGTTTCTC

CTTTTCATTTTCACCGACTTCCAAGTGGTCCACGATCTAGTCTTTGCGTGAAGGGGTGAG

CAAGCCTAGCTCCGTAGGTGTACTTACTGCATTGCACCACTTATTACTTACATCCATTGC

TCTTCTTTGTTTTTTGTCGAAGTGCGTGCATCATTTTGTCAGAGAAGCACATGCGTTGGG

TAGAAATAATTTCGACTGGTCATATTAGACCAGGCTTTCTTGCCAGAGCCTAAGGAAAAT

ATTTGAGTTGGCCACATCGAACCTTATCTTTGCTTTTGAAAAACTTCTTTCCGTGACTTA

TGTGAATTATTTTCCCAATCTACGCCCCTGCTATAAAAGGTTGGTCGATAGAGATACATC

CATTCGTAAAATCGTTTATACCTTGCTTTTGTTCTTCATATATAAGCTGAAGGGAGAGGA

AAGTGCTATGTGCGTTTGAGAACTCCGCCGAAGGGCTTTTGTTCTTGCCACCACCTAGTA

AGTACTATTTTCATCTTTGCTATTTTCATGTCGAGCCATGAGATTTGGCCTAAGTCCGGT

GGTGTCCCACTGAGCTACGAGATACTTAAGGCTTGCACTAGAGGATGAGTTCTTGACAAC

GTGGGGATCTATCCTTTTCCTTTAGGGTTGAATATGTACACTGGTGCCGATATCCCTCTG

GGTTCTTTAATTCTCTCGCCAGCCCACTTAGAGCACGTTAGGTTTCCACTTCATCCCCTC

TTTCACATGCTCATGTTCTTCTTAGGTTTTCACCCCATGCAACTAAACCGACTTCTTACC

TTCTCATATCTGGGTTTCTAGTCATCGGTTTGAAGTGGGGTATGTCTCTTAGTTTTAAGG

ATTTCATGTACCTCTACAACTTGACTTGTGTTAGGGGGAAAGTCACTATTTCTTTTTTAT

GTCTGGGCTTGGTAAAAAGGTATTTACTTGTAAGCCCACTTCTACCAAGTACTAGAAAAA

TCAACCTCTCTTAATCACTGGGGAGTGGGCGACACTTGAGATGTCTAACATATTTATGCA

TTTGAAATATAGCTTATTGTATTTGTACGGGTCTAACTAACCTCTTCTTTTGGCAGTACT

TCCTTCGAGCAAAAGGTATCTCCTCCCTAATGACCGGATTGCTTGGTTGGAGGAGAACTT

GGCTGGTCAGGTGTGGGATATCACATGCTTCGTTGACTCTGCGACTCTTATTCATCTTTC

CTCCCCTCGTCGTACGACGATTTACCGACTTTTCACTTTAGCTGATTCAAACCTGTGCGA

GATAGACTTGTTCAAGGACATTTCTGTCGACATTGAGCTTTGTTGTACCGAGGATTCAGC

TTCTCTTATCCAGGTCAGCTTTCAAGAAAGGGAGGCTGAGTTGGCTCAGCACAGCAGCTC

CGAGGACTGTGTATTCGGGTCTGTGTCTGATGAGTCAGATATGTCAGCGACGCGCTCATT

GTTGGTCCGAGGCAGCAGGGAAAGGAGATCCAAAAAAGAGCTCGAGCCCTCCGTGTCTGC

GAAGAAGTCCCGTCATGTTTCACATGTCGTTTTGGGTTTGGGTTCACCCAATGTATTTGC

TTCGAGTGCTCCGTCCCAACCTTAGCAAGTCCCTTGTGCCCCTACTCATGCTAGCTCACT

GGGTGAGAATGACTTCACGACCATGACTGACGAACAGATGTGGGGTTACCAATCACAGTG

CTTGAAAGCGGTACGTTTGGGATTTGTGCTGGTGTCATCCATTTACCATGTCCTATAATA

GTTGCATGTGTAATGCCCACTTTTTATTTCTTGCAGATGGTTGGAATTCGCAGCGCGCAA

AAATAGCGAGGTTTCAGGCTATGACCTGTGAAATATACGAAAAGACGGCAACCCTCGAAC

TGAGCGGGAAGCGCATCAAGGAATGTGAGACTGAGCTCGAACAACAGCGGGGCGAACTGC

GAAACCTGAGAAGTGGCCAAAGCACTATACTGATAGAGGTTGACTGCCTTGAGCAGGAGC

TGGTGGATGCTCCACGCAATAGGGATGAAGAGTATCAAGCCAACACCAGGCATATCAGGG

TCGAGGTGGCTAGGCAATTCAGATACGGTTGGGTTCAAGGTCAGCAGCTCCCTGAGCATC

AGTTTGTGGCGGGCCCAGATGACTATGAAGATGAGTTGGATTTACGGGCTTCACCTCACC

TGCTGGATTCTCCACTTATGCTGGGCCTTCCTCATAGACCATTCCTGACCCACTTGATGA

CGCGCCCTAGGTTGCTTTATTTCATTCATTGCTTGTTGTATTTTCTTTTCGGCTTTGTTT

TGGTTTTGATATACTCAACTATCTTGGGCCCGTTCCCAGAATTCTCGACTCACTTTATCA

GACTTTATGAGTTATCCACTTGGTTTGAATTTTAACCCTAACGTAAGTACCTGAGGCTTA

ACGTAATGGTTTTTCAAACCTGGCCAATGTCTTCCAGGGATGAATCAGCATTTTCAAATT

CAAAAAACAAGGGATTGGATATCCTATCATCTCCGCATTACGACCTTATCAGGCGTCCCT

TAGTGAGCTACGTCTCTGGGTAAGCTTCTATATAAAGGGTAATTTGGTTTTTAGGTGAAC

ATACCTATCCCACTCATGGCTCACAGACGCAAGCTAAAACTCCATTTCCGCATTTGCGCA

AATGTTCTAAAGAAAGGAGATTCTCCGAGAAGGAGGACTTTATTTCTGTCAAGGAGGGAG

TCCCCACAGCGAGATTCTAAGAGCCCTAACGGAATCATTGAGATATGCTCACAGGATTCT

TCTGCCGACTCTCCGACGAATGCTTTAGATTGCTCGATGGTCCTGAACTTGCTCCATGGG

TCCAGGTCTCGGTCTAGTGATGCCAGAGGTGGAGGAAAGGCTATTATGAGAAGGCACACC

CGGAGCGCAGGTCATCCTTGCTTTGTTGGAAGGACGTGGGATGAAGCCGGTCTGTCTTGC

GTTCCTCGCTACTCTTGTGACTTCGATCACAGGATGTTGTTGGCGGAACGTGACTTACTC

AAGGAGGATCTAGCATATGTCAATGCCCGTGTGACTAGCCGCCAGAGTGATCTTGCTGAT

GCGGACCGCGCCGTTTCAACCTCTCATTCTGATGGCATACGTGACGGATTTAAACAGGGT

ATCCACCACTTCCGTGTCAAGGCCAAAATGGTTTACCCTTACGTGGATTAGGACCGTCTC

TCCTTGCCATCAGACTAGCCTATGTCACTTATGGGTTCGCACCTGTAGAGTCAGTAGGTT

TGATGTACTTTCCACCTTTGAATAATTCGGAGGCCTTCCTCCTGTGAATATCTCATGTTT

TTCCCTTTTATGGCCATAAGAGCCTAATGTTATTGTTATGTTTGATCATGCGGTCTTCGA

CCTTATCACATGGATATCATGTTACTTGATCTCTAACCATGTCATGGTCTCCGATCATGT

CGTAATCTTTGATCACTTCTTTATGCGCGATCTCTAATCGCTTCAAATAGTCTTCTTCAC

AATATACTATGACCAGTAATGCGGTATAAGTAATTCCCAAAATCCGCTAGAAGAAAGAAT

ATAAACAAGCAAAATTTTTTTGATTATACATAATTACATAACATAATTGCCAAGAAAAAT

TTGTTTATTTTTAGAGCTTGATGTTGATAAATCCGCAGATCTAAATAGATTCTTAAAGTA

GACAGACAAAAGGGGTTAGAGACCACTCAATAAATGAAATACCTAAAAGGTTTCTTTTGA

GTTATTTGAGAGTTATTCAACTTGAGTTATGAGGGTGCAAATACAAATAATATATATATA

TTTTTTCGGTTGGGGGAGAATAAGAAAAAATACTTAAAATCAATAATTTAATTAATTAAT

TAATTTTATGCATATGTTTGATGTTATAATTCTATAGTATAGACATAATTTATAATTTTC

TTGAGCCGTACAAGGAGAAAACTTCTTATACGTTTCTTTGGGGGGGGGGGGGGGGTGGTA

TTGTTCATCTATCCCAATGAGCCATTTATCGAATCGTTGCAATTGATGTTCGATCCCAAT

AAAAACCCTAATGAACCTAAAAAAAGAATAAAGGCCCAGCAAAAATTACTTGTTTTTCGA

GACCCTCTATAAGTTCTATCTATATACGTTCTGATCGCCAACTCATACTATAACTAGACC

TAGTTGCATTTTATTGGAATTGGGAGAATAACAAAAATAAATTTATTTTACTTTTTTTGT

TTCCGAAGTAACTCTCATCAACCAGCACTATTATGATGTGAACGTTTAGGGGTAATTCAT

CGAATTTCTTAGATCCGCATGGCAATCGGCAGTGAGGTGAGGCGAATGACGAAGAGCTAG

TGAGTGGGTAAGACAAGGTGTGGCGCAGTCGAAAATGTTTGATTCATCTATTATTATAGA

CGATACTAACAAAAATAGAGTAACATAAGTAAGAAATCAATTCATCATTTGTGACATTTA

ACCATGTCGCACTTTTGACTTAACAGATTCATCGGTCAACTCTGGAGTTGACTAAACTAA

TATATGCATATAACTAATACATGTATATATATATATAAAATATTTAAATTTAAATTATAA

ATAAAGTAATATGTTCCAGGGGTTGGCACCTCGATGTCGGCTCTTCGCCACCTGGGGCTG

TAGTATGTTCCAAGGGTTAGGTTGTTTGCCCATTAAAGCGGTACGTGAGCTGGGTTCAGA

ACGTCGTGTGACAGTAACGATACTCGCCACGATCGTTATGTGTATGATACTAATTCTCAT

TATATTTGGAATAATCACATTAATAGTTGCATTGACAGTTGTCATCGTTCTCAAATTTGT

ATTGATAGTTATATTTTAAGCAATAGTGACAATTACAGTGACAACTACATTTATAGTTAC

ATTTGTAGCGAAAGCGTAAATAGTAGTAAAAGCGAGAGTTTCAGTATAAAAACTAGCATA

GATGGTAGTGATTTTACTATAAGTTCTAAAAATTTAAATGTAACTAAAAAATACAGGCAT

TTGTGGATTCAATGCGAATATTGTTATGAATTAAATTATAAAAAAAATTTAAAATTTAAA

ATGAATATTTATGAATAGTGTGGATGTCATTTGAAATGAATATAAAGAACCTTTTTCATA

AAAATCTCACATACTAATAAATTTATATTCCAACACAGAATGAAAAGGACAATATACAGG

ATGGGAGAAGTTGTGATACTTGGCTCAATCCATGATCCAAAAACGCAGGATCAGGATATT

CAGGATATAATAATATATAAAAATATAAAAAAAGAATATACTAAACTAATAAAAAAAAAT

ATATTATAAGAATACGCCAATCCTAAGGATCCATAGGATTAATTGTGGATCCAACCCAAC

AATAAAAAATTAGTTGTTTTGTCTTATATTTTTGTATTTAAGATCTTGTATATCTATATA

AGTATGTATCTATCAAAAATCTATACATCTACACAATAGGATTTTTTATTGTATTTGGCT

CAATCCTTTTACTTAAAGATTTGGCCGAGTTTAATTTCAATTCAATTAAGAGAACTAACA

GAATAAGGACGAATCTTTTTTTTTTTTTTTTTTTTCAAGACTTACTTGGATCATAACACG

GATATCTATTTAGTAGAATATGGTATAACATGTGGCTTCCTTCGGGCATAAGCTCTTATG

TATGTATAAACATGATATTATGGGTAAATGAATTATAACTATTTTCAAATAGATCGGGAT

CGGGGAGAATTTCTGAAATGTAAAGTCAATATATCTCTATGTATTCGGAAATCATGTGAA

AGGGATGTTACTATTTTAGTACTTTTAGTCTTTTGATCAAGCACATTCTGTGCCCATGGA

TTAGTATATGCATTTTCTTATTTACAATGTCTTCGACCACATGGATTCGGGTGATCCTAA

TTTAAACAATCATGGCCTTCGACCAAACATTTCGAATGTGAAGAAAACATGTGTTGCATT

AAGCGTTCATCGAACGATGGACAATTTAGGGGAAATTCGAAAATGAAACCAACAAGCAAC

AAAGAAATAAAAACTATAAAATCAAATGAAATACTTCCTCAGATGAGTCGCATTCCAATG

GCGTGGTATGTATTGACCGCTCTCGATATGCCTCAGCATATATGCCCATTTTCTATAAGC

TTCCATTACCTCGTACAGTACTTCCTATGTTCGTTCAAATTTTCATGCGTTTAGGTCCTT

GGTATTCTTCATGACCTTCCTGATTACCCACTCGCCTACTTTGAATTTTCTCAATCGTAC

TCGTTTATTGAAGTAATGGGCTGCCTGGTTCTGGTAGTTTGCAAGTTGTAATGCTGCAGC

TTCCCACTGTTCTTGAAGCAGGTCAAGGCTGTGAGCTAGCTGCTGCTCATTGTCATTGCT

CCCTACCACCAATGTCCTGACTGTGGGTAGTTCAATTTATGTGGGGATGACGGCTTCAAT

TACATAAGCCAAGAAAAAAGGGCTTTCGCCGGTTGGTTTTCGTTTGGTAGTACGGTATGC

CCATAACAAGTTTGGCAGTTCTTCTGACCATTTGCCATTTTCTATAAGCTTCAAGCAATC

AAAGATCATACGATTAGTTTTCTTAGCTTGCTCATTTCCTTGAGGGTAACTTCGGGATGA

GAACTGTTGAATGATCCCTTTTTCCTTGCAGTAACTTCTGAATTTACGGTTATCGAACTA

GGTTCCATTGTCTGTCACGATGGCCCTCGGCATCCCAAAGCGACATATTATATTGCGCTA

TACGAATCTCACCACATCTGTTTGAGTCACCTTCTTTTAAGCCTTGGCTTCCACCCACTT

GGTGAAGTAGTCAGTGGCCAAACTTCTTGGCCCCCACTGCAGTGGGTAATGGTCCCACGA

TGTCCATCCCCAACTTCTTGCCCCCAACTGCAACGGGTAATGGTTCTACAACAGGGTTAA

GTTTCTTGGCTGGTTGTCTGATAAGAGGAGCATGACTTTGATATTTATCACATTTTCGTA

AAAATTGTTTTGCATCTCGGGCAATGTAAAGCCAAAAATATTCTTGGGTTAGAGCTTTAT

GGGCTAACCTCCTCTCACACGTGTGGTTTCCACATGTCCCATGATGTATTTCTTTTAGCA

TCCATTCGACTTCACGTGAATCAAGGCATATGAGATATGACCCTGAAAAAGACTTATGAA

AAAGCTTAGCATCTATAATTGCATACATGACGGCCTTTATTCGTAATTTTCTTGCATCAA

TAGGGTTATCGGGTTGGATTTCCTTCGTGAGATAGTTCATGATTGAAGTCATCCATGTTT

CTTCTTCGGAATCAAGCTGCATAATTCCAGGATCAATTTGTATACTGGGATTCGATAAGA

GTTCCACCGGGGCGATCCTTAGGTGTCACATCCTGGGATCGGCTCGGCCGTAGCACGATA

TTGTCCGCTTTAGGCCCCCCTCTCTACCCGCACGGTTTTGTTTCTGGGAGCTCACGACCA

ACTTTCCAGTGGGTCACCTATCCTGGGATTGCTCTGGCCCCCAACTCGCTTAACTTCGGA

GTTCCTACGACTCCAAAGCCAGTGAGCTCCCAAAAGGCCTCGTACTAGATGGATGCGGAT

GTGCACACATAAGGCACATCACCCCCTCTCCGTTGGTTGATGTGGGATCTTACAATCCAC

CCCCCTCAAGGTCCCGACGTCCTCGTCGGCTCACTGGCACCACATGGCAGAGTGGCTCTG

ATACCAAATTGTTACATCCCGGGATCGGCTCCGCCGTAGCACGATATTGTCCGCTTTGGG

CCCCCTCTCTACCCGCACGATTTTGTTTCTGGGAGCTCACGACCAACTTCCCAGTGGGTC

ACCCATCCTGGGATTGCTCTGGCCCCCAACTCGCTTAACTTCGGAGTTCCTACGACTTCG

AAGCCAGTGAGCTCCCAAAAGGCCTCGTGCTAAATGGATGCGGATGTGCACATATAAGGC

ACATCACCCCCTCTCCGTTGGTTGATGTGGGATCTTACATTACGTCTTCCATCGACATTG

ATGAGGTGTGGGCCAAGCGATCTGCTTGGCCGTTCATTTCTCTCGGTATTCGCTCAAGCT

CTATTTTTTTGAACCTGTTGGCTAAATCCTTTACCAGCCGTTGATATGCCCCTATTCGTT

CATCCTTAATCGTGTATTCCTCAGTTGTCTGGCCGACTATGAGGCGAGGGTCGCTGGGGA

CAACTAAGTGTCTACCTTCAACGTCCTGGCTAATTGGAGTCCTAACACTAGGGCTTCATA

TTCCGCCACGTTATTTGAGGCGGGGAAGTCTAATTGGACGAAATACTCCAGGTCCACTCA

TTCGGGCGACGAGAGGACGATTTCCACTTCAGCTCCTGCCTGATTGGGTGATAATTTGGT

TTCGCTGAGGTGCGAACCTCCTCTGCTTCTACTGCGACGGGGGATGGTTTGGGTTAACGT

TCAGCCGATGACACGCAATGTCTGGGTCAATCTCCGGCATATCTATATGTGACCACGCGA

ATACGTCCTTATTCTCTCTTAATAACTTCGTTAGTCGTTCTACCTCATCTTCAGGTAGGA

GGGAGCCTACCAACACTTTATTATCAAGATAAGAAGGATCCAGAACTTCCTCATTGAGTG

GCTCAATCGTCGATTTCTCTGAGTTATCCTCCTCCTTTTGCTGTGGTGGGTCCTCCATTA

ATTGCGATAAATCCTCGCCCTGCTGCGAAATCGCAGCTTAAGGCTAGTTGCTATGCTGTA

GCATCTTCTGGCCTCCACCTGATCCCCCATGATATCCACTGTGGTTCTCCTATCTTCAGA

TAAGCACTTCATCACTTAGCAACGTGTCGATACTTCACCATCCATTCGATGAATCCAATT

CCTTCCCATTATAGCGTTATACGCGCTCGGCGCATCCATAATTACGAAAGTAAAGAATAA

GGTCTTACCTGAGGCAATAACTGGCATAGTCACGTCTCCGATCGGTTGCGCTTTTGTCCT

TCGAATGCTTAGTCGATGTTGGGTTTGGTTGAATCTCTTTTTCATCCAACTTCATTATTT

TGAAGGTGCTTCAGAATAACTCGTCTGCACTACTTCCCTCTGTCAATCATTACTTGTTTC

ACTATCGAATTGGGAACGTGGAGGGTCACCACTAATGCGTCGTTGTGAGGGAACTCAATT

CCTTCCATGTCTTTCTGCGTGAACAAGATGACTCTAAACTTTTCTAAGGCTTTGGTGGAA

TCGAGCGCAGATGCGATGCCGTTTATGCGTTGAGCCTTTTCAGCTCGTCTTCTTTCAAAC

CGGGATTAGGCCTATTTTCCTTCCACGGGATTCGGTAGACCATGGATGGTGTTGATCTCT

AGCAATTCTTCATTTACGATATTTTCGGTCATTGTTTTCGACCCACCTTCTTCATTTGCC

TTGTTCTCGAGTTCAGCGCGGTACTGTGGTAACATTTCCTTTCTCAACATTGCCTCTACC

TGCTACCTGAGACTTTGGCACTCATCGGTAGTGTGCCTAAAGTCGTTATGATGGGCGCAC

ATCTTGTTTCTGTCTCGCTTCTTTGGGGATTCGCCGATCGCAGGTGGTATTCGAAATATA

CCTTTACTTTTATTCTAGTGAAAAAGTTTGGCCAAACTCACCGTGAGTCTAGTGAAGGGT

CGGCTTTGCTTAGATCCATTGCTAGAGTCTTGTCTCGAGGAGTATCGCTGCGTCTCACCT

TTTGATCTGGTGGAGGCTTTGGGATTACAATGGTGGAAGTGGCGCGTTTTGATTGGGTGA

GTTCCTTTTCTTCCAGTCTGATGATTCCATCCGCTTGGCCATCACTTCCCTCATATCCAC

TGGTAGAGTCTTTACTAAAGATATGTGCATTTTTGTCCCGGGTATGACCCCCTCATGAAA

CACTAGCAAAGCAGTATGAGAATCACACTCCTCAAGAGTAGACATTTCCTCCGTAAAACG

CCTCAAATAGTTTTTAAGGCTGTCTCCGACACTTTGCTTCGTGCTGAATAGAATCGTGAC

GTCCTTTCTCCTCTTTTGATTGCAAATGTATTGAGATATAAACATTTCACAAAGTTGGGT

GAAATCACCAATAGACCTTGGTTTCAACTGTCTGAACCAAATCAGGGCCAATCCTGACAA

ACTTGAGGGGAACAACTTACACAGCAGGGCCTCGTCATCTCTTTTGAGAACCATTTGTTG

TTGGAAGTGATAAATATGTTCAATAAGATCAGTGGTGCCCTCGAACAGCTTAAATTTAGG

TTGGGTGAACTTAATTGGCTTTTTGGCTTTCAGGATGTCCTCTGTGAATGACGACTTGCA

AATTCCTTTCGCCACCTCTAAAGCTAGTTCCGCGGGCGTTTGTGGCTTGTACCCTCGAAC

AATCTTCTTGATTTGATTGCGCAAATCAATATCCTTTGCCTTTTTCGCCATTTTCTATAG

GTCAAGATCTATGTCTTGATCTCCTGCAGTGACCTTGTCACGGACGCTCAAGTGCATGCT

GCTAAAGGAGTCTCCCTCAGAGACCTCATGTCTCTGCTTTCTCTTGCCCTCTTTTGAACC

CTTACGGCAAGCAGTGTTCTGCGCCTTCTCCAGAGCTTTCTCTGCTACCTTTCTTGTTTT

GAGCAATTCTTTCATGATTTTCCTCAACTGGATAACAGCAGGGCTTTGTTCCCCATCCTA

GGCGTTAGACATTGCGCTTCTAGGTTGAGGAGTGGACATACCATTCCCTCGGATGGAGGT

GTTTAGAGTTCCATGTGCCGGTTTAGTCATATTTATCATTAATTTTGCAGGATTCCCACA

GACGGCGCCAATTGTTGGAGCAGGATTTCTTTCCAGAAGAAATACCTCCACAGGTTCTTG

ACTGAAGTCTGCACGCTTCTTTGGTTGAAAGAGAACTCCGAATTACGTCCTCGAGGTCGA

ACGGTGGAAATGGGGTGAGTACCTTCAAAAGTCACTCTGACGCTCAAGTCAGTGTTTTTG

CAGCGTACTTTTAGTGTTCAATTGAATTTCTCGTACCTTTGTTGGAGGGTTAGCCGGGTT

TTTATAGTGCTTTGAGTTCTTGGGTTTAGCCCCACTTCCCTAGCTTTGCGCATCTCGTCC

TCCACTTCATGGTAATGGGATCAGATGAGCGTGTCTCCCTCGCTCCGCACTTTTCTAGTG

GGAGGGAAGTTCGATCATCCCACGTCTTCTAGTTGTGGCATATGGGTTAGGCCATGATAC

GTGAGATTTGGTTCCCACATGGTCGGAGGAAGTTTGTGTAGGAGGGATTTGTGGGATGCG

AGGGATTGGTAAGACTACCATTGGAAAAGGCTATATCTCATGGAATTCGAAATCAATTCG

AAGCTTTTAGTTTCATTTCTAAGGTTGGAGAGATATCTAGAAAGGAAAGTTTATTTCATA

TCCAAGAACAACTTTGTGATCACTTGTTGAATAAAAAAGTAACTACAAAGAATGCTGATG

ATGTGATATGTAAGAGATTGTGTGGCAAACGAGTGCTTATAATTCTTGACAACGTGGATG

AATTAGAACAAGTAGAAGCTGTAGCTGGAAAGGATGATGCAGAGTTATCTAATCGGTTTG

GTAAAGGAAGTAGAATCATCATAACCACAACAGATGACAGATTGTTGGTAAATTATAAGC

CAATGATATGTAGGATTGATAACTTACTCAACAAGAAGCTCTTCTTCTCTTCTGCTGGAA

AGCCTTCAAAAAAGACCATCCAATGGATGGTGATGAGAAGTTGTCTTATGAATTTGTAGA

TTACATTGATGGTCTTCCTTTGGCTCTAGAAGTTCTCGGTACCTCCTTAAGGGACAGAAG

TGTAGAATATTGGTCGAGTAAATTGGCTCCTTTTTAAAAGATAATAACTACTCCAGTGAG

AAGAAAATTATTGATGTTCTTAAAGCAAGTTTTGATGGATTAGAGAATCAAGAACAACAT

GAAATGTTTTTGGACACGGCATGATGCATGATTTACTACAGCAAATGGGCAGAGAAATTG

TTCGTGGAGAACCCAAAAAGGAAGGAGAATGCAGCAGATTGTGGCTTCATACAGACGCCC

TTCCTGTACTGAAGAAAAATAAGGTAAGAATTTGGTAGAACCATATAGTACTCCCTAAAA

AAATGCATTTTGACTTTACCAATGCTAATATTTAATGGATTTTGAGAAACAAATTTTCTT

TTGAATGCCTATGTTTCATATTCACTTAGATTAAAAAGTTGTTTAGTTATTATTTTAAAG

GTAGATGTCTACTTCATTTGAATTTCAAATTTTTGGTTTGCTGAAACGAGCTTTCAAAGT

TGCTAATACTCTATGGCATGAATACTTTTCTATTATGTGTTGGGGACTCCTTATTTAGTT

AGGATTTCGATTACTTATCCGTTTGTTTAGTCCTATTGTAATTGAGATTTATTTCCTATT

TGATCTCTTTTAAGGTTGACACATCTTTCTACTTCTTTATACATAACTTTATATTGGGAA

AGTAAACATATCTGAAAATTATCATATACTTTGTTTGGCATGGTTGCATTTAACCTTCTT

ACCTTGATATATTCCCATGAGGATATGGCCTATATCTTGGCTCCGACGAGTGCTACTCTT

TAGGGGGAGGGAGATCAAAGAGAAGTTGTGTTTGCGTGAATTAGGTCAAGTTGATATAGA

AGAATTCTCCTTTAACGTTGCTGTTTATAACTTTGCCTTTGTCCTTGCCTTTGCTCTTAC

TTTGCTTTGCCTTGCCAGTGCTTTGTGTTGGGTTACTATGATATTATATGGGGTAATGCT

TATCATTTTAGGATTGGTTTATTTATGACATGTGGTTAATGTTAAAAATAGTCGTAGGGT

ATTTTCTGTTGGCGTGAGCCATAATTCAGGGATCTCGTGATCCTTGATTTTCTGGTGTAA

TTCACGTTACCTAATTAGAATAATTACCTAGCTAGGATTCCTTGTAATTTTTTAATTACT

TTTTCTTGTAGGAAAAGTCAACTTGTACTTGTATATATATATATATATATATATATATAT

ATATATCCTCCTCTTGGAGAAATATAATATCAACCACATTTATTCAATCCTGTGTTTAGT

TTGAGTTGGTATCAGAGTCGGTTAACTCTCAAACAGAAATACAAACCCTACCCATCAACG

TCCTCTAGCGCCAAAAATAGAAAAAAAGAAAAACATAGCTGCCACAAAACAGTAGCTAAG

CCCTTATCGTCGCAAACCTAGTGCGGCAAAAGACTCCTACATCACCAACCTATCCAAACT

TGTTCTCTCTCCTCCATGGCTTTCAAAGATACTCCTGACCACCACACCGCAGCTGCAAAA

TCTATGCTTCCTGTGCTTCAAAAACCCACAAAAATCTTGACATGGTGCAACCAATTGTTA

CAGTTCGCAATGATACTTCTGCTGCCCCATTCACCATACGGTTGAATGGAAAAAACTATA

ACACTTGGTTGAAGATGATGTTGCTTAATGTGTCAGGCCAGGGAAAAAGAGGTTATTTAG

CAGGGAAGGTGGCTTAAGTGGAGGAAGATGCTCCGGGCTTCGATTCGTGATGTATTGAGG

ACTTCGTTGTTAAGGGATGGTTGATAAAAACTGTGGAACCTGATTTGGTAGAATTGTTCT

TAGATCTATCAATAGCTAAAGATGTTTGGGTGAGTACAACCTAGATGTATTATGACGTGT

CTGATGAGTTGCAGATTTATGAATTACGGTGTAAAGCAATCGTATTACTCAAGGAGGTCA

CTTTAATGCATCATACTCTGCTGAGCACAAGAGCATTTGGTTGGAACTAGACTGTCGTCG

TCCAATCAACATGAAGTGCCCTGATGATGTGAAGATTCGACAAGCTGAGATGCACAAAGA

CGATATTTTTGATTTTCTTGCACGTCTAGATGATAAGTTTGATAAAATTAGATGAGATTT

GTTAAGGTTGAGGCCATTTCCAAAATTGGAGGAGTTGTTTACTTTTGTTCGTAAAGAGGC

TGAACGCCGGGAGACGATGCTTAAGAATGATGGAAAGACTGAATCCTCTACTGTAATGGT

TTCTAAAACCCATACTGCTAGTTTTTCTTTTCCCCGTCCAACCCCTGAGTAAAAAGAAAA

CATGCATCGCACTTACTGCAATGGGAATTGTCATACAGAGGCCACATATTTTCACAAGAT

GGGGTTTCCAGATTGGAGAACGAGATGTTTTTTCTGTCGCCACTTTTATGTTATCATGGA

TGTTATGTTTGTGGAGAACAAGATGTTTTTTCTATTAGACGTGCCCAATCATGGGGGAGA

ATACAGTAAAAGAACCACATAATTGATTTGAGATAGAAATGCGGCGGAAAGAAGCACAAC

ATAGTGTTCTGGAACCCCAGCTGCTCAGCTCAGCAGAGTTGAGCCAAGTAGCTGCTAGAA

GTAGGCTGCCGACCAGTCCAACCGAGCCGAAAGAGACATCAACGAGCTTGAACTGCACAT

CAGTGACCTTCTCTCTCGAACATACCTATTCATACTCTACTGAATATCCCTGAGGTAAAT

TCTTCAGAACCCAGTGCTTCCCAAAATATTGTTGAAATTGTGAAAAAACTACGTGCACTA

GCAAACAAGGGAATACTACAGGCAAATTCTCACCAGAAGGCATGGTGAAATATGCCATTG

CTAATTATGTCTCCACACACAGATTGTCTCCTAAAGCTAAAGCATTTGTCAGCAGATGGA

ATGAATTAAAATTCCTAATAGATTGGAAGAAGCTTTGCAAGACCCAAAAGTGGGTGGAAG

CAATGAATGTGGAGATGGAGGCCTTACAGAGGAATGGAACATGGGAGATAGTTCTGCAAC

TTGAAGGAAAGAAATCTGTTGGATATCGATGGGTGTACACAGTTAAGCACAAAGCTGATG

ACACAATAGACATATATAAAGCAAGACTTGTGCCCAAAGGGTACACATAGACGTATGGAA

ATGATTATCAGGATATGTTTGCACCGGTGACAAAAATTAATACGGTGCGAGTTCTATTGT

CCTTAGCAGCTAATTTGGATTGGCCTCTCAAGAAATCGATGTGAAGAATGCCTTTATGCA

TGGAGATCTAGAAGAATAAGTGTACATGGACATGCCTACTGGCTATGGGTTGTCTAATAA

CAGTGGGAAGGTGTGTAGGCTTTAAAAGGCTCTTCATGGATTGAAACAGTCTCCATGAGT

ACGGGTTGGGCGGTTCATTAAAGCAATGAAGAAGTATGAATATCATCAAGGTAACTCAGA

TCATACCCTATTTATCAAACATAAGGATGGTAAGGTTACTCTGCTGATAATCTATGTTGA

CGATATGACAGTTACTAGTGATGATGTTGTTGAAATGGGTAAATTATAGAAGTACTTTGA

GTTTGAGATGAAGGACTTGGGGGCTTTGAAGTATTTTATAGGAATCGAAGTAACTCGTTC

TACTACTAGTATTTGTATCTCTTAAAGGAATTATGTTCCAAAACCTGTTGATAGAAACTG

GTATGTTAGGGTATGCACCAGCTGAAACTCCCATTGTGCAAAATCATCACCCAACAATCT

ATCCTGATCAAGTGCCAACCAACAAGTAGTGGGAAGACTAATTTATCTTTCACACACTAG

ACCAGATATCAGATATGCAGTTAGTGTTGTTAGCGATTTCATGCATTCTCCTAATGAGGA

TCACTTAGCGGCAGTTGTGAGGATTTTGAAGAAAGCTCCAGGCCGAGGCTTGATATTCAG

AAAACTGGGACATTTGGATGTTAAAGGTTATATAGATGCAGATTGGGCAGACAATATTGT

CACATCCCGGGATCGGCTCTGCCGTAGCACAATATTGTCCGCTTTAGGCCCCCCCTCTCT

GCCCGCACGGTTTTGTTTCTAGGAGCTCACGAGCAACTTTCCAGTGGGTCACCCATCCTG

GGATTGCTCTAGCCCCCAACTCGCTTAACTTCAGAGTTCCTACGACTCCGAAGCCAATGA

GCTCCCAAAAGGCCTCGTGCTAGATGGATGCGGGTGTGCACATATAAGACACATCACCCC

ATCTCCGTTGGTTGATGTGGGATCTTACAATCCACCACCTTAAGGGCCCGACGTCCTCGT

CGGCACACTCGCACCACACAGCAGAGTGGCTCTGATACCAAATTGTCACATCCCAGGATC

GGCTCCGCCGTAGCACGATATTGTCCGCTTTGGGCCCCCTTCTCTACCTGCACGGTTTTG

TTTCTAGAAGCTCACAAGCAACTTCCCAGTGGGTCACCCATCCTGGGATTGCTCTAGCCC

CCTACTCGCTTAACTTCGGAGTTCTTACGACTCCGAAGTCAGTGAGCTCCCAAAAAGCCT

CGTGCTAGATGGATGCGGGTGTGCATATATAAGGCACATCACTCTCTCTCCGTTGGTTGA

TGTGGGATCTTACAAATATATAATAGATAGATGTTCTACATAGGTTACTTCACTTTCATT

AGGGGTCATTTGGTTACATGGCGAAGCAAGAAGCAGAATGTGGTGGCTCGATCTTCAGCT

GAGGCAGAATACAGGGGCATAGCACAGAGAGATTGTGAGCTTCTTTGGCTAATGATACTC

TTGTATGAAATTGGTTTTCCACTAAAGAAAGTCATGGAGCTTTACTGTGACAACTAAGCT

GCGAGAGAGATAGCAAATAATATAGTTCAACATGACATAACTAAGCATGTTGAAGTGGAT

ATGCATTATATTAAGGAAAAGCTGGTAGATAAGCTAACCGATATACCATTTGTAAAGTCA

GAAGAACAGCTAGCAGATGTCTTAACGCACACGATGTCAGCAAAGATTTTTCATGACTCA

CTTGACAAGTTGGGCATAGGAAATATCTACGCACCAACTTGATGGGGAGTGTTGGCGTGA

GCCATAATTCAGGGATCCTGTGATCCCTGATTTCCTGGTGTAATTCACGTTACCTAATTA

AAATAATTATCTAGTTAGGATTCCTTGTAATTAGCTAATTACTTTTCCTTGTAGGATTGT

ACTTGTATATATATATATATATATATATATATATATATATCCTAATCTTGGAGAAATATA

ATATCAATCACAATTATTCAATCATGTGTTTAGTTCGAGTTTTTCTTTATAGCTTGGATT

GTACTTGTTTTGCTGGTATCAAATCACAATTCCTATTTCATCTTATCTTCTTCCTCTCAT

TCTCCATTTATGATCCGCATACCTATCACTTTGCCTTGCTTTGACCTTGACTTGACCCTA

CTTTTTAGGGTTTGCTCTAGTGTTTACATTGCGATCTTTGCTTTGGTTTATTTGTTTTTT

AACTTGTGCATCCCTACATCACTAGTGCTAATGGTGTCCCTTCTCCCGTCACCGGTATAA

TTAGGAGATACCTTCCTAATTCTTTAATGTATCTAATTCCTTGTATAACACATGTAACTC

TTATATATATATATATATATATATATATAGATATCCTTATGGAGAAAAATAAATATAACC

TAATACATTCAAACCATATTCATATTTTAGCACAACGAAGTGGCGCCCAGAGATGGGCAG

TGGGTGGCAGGTAAGAGATGGGCGGTGACGGAGGTGGTGGAGTGACTAACCAGCGAGGGG

ATCGAATGGAGGTAGGGGAGGTGGCGGTTGGATGGAGGAGGAGGTAGCGGTGGCTTTGGC

TAGCTGGAGGTGGATTGCTAGACATGTAGCATAAGGTGATTGACTTCAAATAAATTTGTT

AAAATATAAAAAAGAGTGACATTGTGTGTATTTTTGAAATTTTTATATTACATTATTGTA

AAATTACACCTTACTAAGTCATTGACCTCAAGTAAATTAGGTAAAATTAGTTTCCTATCT

ATCACAATAGAAAACTCCCTATTGATTTTAAACCAAAGCTATATTAAAAAAGATAAATGA

AAGATAAGGGCGTGAGGTATTTTCGATAGTGCGAAAATCACCTCTCTGTCCTAAACCCAA

TCCGGTGCTCTTAATTTTCAGTATCATAACAACCCAAGTTGTGTTCTTGGACAAAAAGAT

AAAAGGGTTCTAGATGTCGAAACCATGAACTAGAAATACTATATGATCGTTTAACGAATC

AGGAATTCATTTTCACAAATGCTTGGAGAAAGAAACAATTATAGACATACCAACAGAGTG

TGGAATTGGAGGGAAATTGCTTTAAAATATAAGCATTTTCAAATCTCTCTCTAATTAATA

GTGGCAATGATTATGCCTTCCATTGTTGACTGAGTAAAGGCATGTGAACCTGGAAGTCCA

TGAAAGTAGTCAACGAAATTTCATCGTTCACACTTCACACGATCAGTTTTCATATGTTTT

TTGTTTGCTTTCTGGTTCCACACCATTTTTTGTACACCAGAAATCAAACAAGTTGGCATA

AAATAAGAGTAACAGATCGTATTTACACGAGAAAATCAAAGGAGCTCACTTTAAAAAAAA

ATTTAATTAGTTGTCATCATATGATAATTCAGAGTTGGAGGCTGGACTAGGGTATATAAC

TAAAATAAGATGGCTGACTGCTTCAAAGTTCCAAATCCTTTTGACTAATACTAATCTTGG

ATTCTAATTAGACTAATTAATCAGGTTGAGCTGTGAGTATTTATTGTCTTATATTGCACT

CTGTCAGCTCAGCTACCAAGCTAGTTGGTTCTGCAAACACTTGTAAGTACCTTGCAAAAG

CTTCCATTACCAGATAACAGCGGGTTGGTTACTGATTTAGTCACTCCAATGGCCGATCCC

ATGACCACCCAAACCTCCTCAGCATTGCCTCCTCCTCCGACCCGTCCACTGGAATATGAG

GTGTTCCTCAGTTTTCGAGGTTTTGACACTCGAAAAGGTTTTACTGACCATTTGTACAAG

GCTTTGATTCGGAACGGAATCCACACTTTCAGGGATGATGAACAACTGAAGAGTGGAAAA

CCCATTTCAAGGGAACTCTTCAAAGCAATTGAAGAATCAAAAATTTCAGTCATCATTCTT

TCAACAAACTATGCGACCTCCACATGGTGTCTGGATGAACTTGCGAAAATGGTTGAACTG

GCAGCAAACAATGAGTCCAGATTGATTTTGCCCGTGTTCTACAACGTGACGCCATCCGAA

GTACGAGAGCAAACTGGAGATCATTTTAAAAAGGCATTTGCTCAACATGATAAAGATTTT

GAAGGTGCGCCAGGAAAGGTGGCAATGTGGAAGAACTCTCTCACTGCAATAGCCGAACTC

GAAGCCGAAGGATTTGATCTGACAAATTTTAGGTAAGGCTGGGTCTTTTAATATGTATGA

TCTGACAGATTTTAGGTAAGGCTGGAAAAATCTATCAAGTATCATGGTCTAAGCTTTTTT

CCTAATAATATTTTTATTGGTTTTACTTGTTAGATCTAGGAATAGCATGGAACAACAAAA

ACTTAAAAACTTAAAAGTTCCTTAAAACGAAATAGTACCCAAACGAGCCTTAGCTAGCTT

TCAATAGAAATCCATTTAGTATTAAAGTAGCAAGGCGTCAATGGAAGGCTTGAAGCTCAA

TTATATTGAAGAATTCAAGGAGAGATTCACTAATAAAGGACCCTATGCACTACATTAGTA

ATCAGAGTTGTGTCTAGGGAGTGTTTGATCGTGATATCAAACTAGCTAGATTGATCAAAT

ATTGTATAAAGTCTACAAGAGTATCACTCAAAGAAAAACTCTATAATAGGCCACATAAAT

CAAAACCGAATACCCCATTTTATTGAAGTACAGGAGAAGTCCACATGACATTAGAAGGGT

GCATGTTGAGAGGCCGGAAATCAAACTACAAGTATAGTATGACTAGAAAACACGACAAGT

TTTACATTTTTTTCGTCTTTAATTATGAGGTATACTGACCCAAATACTCAACAGAATGCT

CTAACTTTTTGCAGATACGAGACAGATATGATTGAAAAGATAGTTGAACGTATTTTTGGT

GTTTTGATTAAAACATTCTCAAATGATGATTTAAAGGACTTTGTTGGAATGGATCGTGTA

AATGAAATTAAATCCAAAATGAGTTTGTGTATGGGTTCAGAAGAAGTTCGTGTAATAGGG

ATTTGTGGGATGCCAGGGATTGGTAAGAGTACTGTTGCAAAAGCTCTTTCTCAAAGAATT

CGCAGTCAATTTGATGCTATCAGTTTCATTTCTAAAGTTGGAGAGATATCTAAAAAAGAA

GGCTTATTTCATATCAAAGAACAACTTTGTGATCACTTGTTGGATAAGAAAGTAACCACA

AAGGATGTCGATGATGTGATATGTAAGAGATTGCGTGACAAACGGGTGCTTATAATTCTT

GACAATGTGGATGAATTAGAACAAATAGAAGCTGTAGCTGGAAGTGATGGTGCAGGCTTA

TCCAATCGGTTTGGTAAGGGAAGTAGAATCATCGTAACCACAACAGATGAAAGGTTGTTG

ATAGATTATAACCCAGAAATATACACAATTGATAAACTTACTCCGGATCAAGCTCTTCTT

CTCTTCTGTCGGAAAGCCCTCAAAACAGACCATCCCACGGATGCTTTTAAGAAGTTGTCT

AACGAATTTGTAGATTACATTGATGGTCATCCATTGGCTCTTGAAGTTTTCGGTCACTCA

TTATGGAAAAGAGAAGAAGATTATTGGTCTACCAAATTGAAGTCTTTAAAAGATAAAGGC

TACTCCGGTGAGAAGAAAATTATTGGTGTTCTTAAAGCAAGTTTTGATGGATTAGAGAAT

CAAGAACAGCAGGACATGTTTCTGGACACTGCATGTTTCTTCAAAGGCGAGGATGTATGT

CGTCTAGAAAAGATATTTGAAAGTTGTGGCTACTATCCAGGCATCAACATAACCATCCTT

TGTGAGAAATCTTTAGTAAGCATTGTAGGGGGAAGATTGTGGATGCATGATTTACTACAG

AAAATGGGCAGAGGACTTGTTCTAGGAGAATCCAAAAAGGAAGGAGAACGCAGCAGATTG

TGGCATCATACAGATGCCCTTCCTGTACTGAAGAAAAACAAGGTGAGAAGTTTAGAAGAA

TCATACAGCACTCCCTAATAAATGCATTTTGACTTCATCATCACTAATATTTAATGAATT

TTGAAAAACAAATGTGCTTTCAAATGCCTATGTTGCACATTTACTTAGATTAAAATGTTT

TTTATTTAATTTCAAGATAACATGTCCACTGTATTTGAATTTTGAATTTTTGGTTTTTTG

TTGCTAATAGTCTATGGTATAAATATTTTTCTACTATGGCTATTAGGGGACAGATGCTGT

TCAAGGTATTTTCCTAAGCTCGCCTCAGCCAGACAAAGTACACTTGAAGAAAGATCCATT

CTCAAACATGGACAATCTAAGATTGTTGAAGATTTACAACGTGGAATTTTCTGGATGCCT

TGAATATCTTTCAGATGAGTTAAGCTTATTGGAATGGCACAAATGTCCTTTAAAATCTCT

GCCTTCAAGTTTCGAACCCAACAAACTTGTTGAACTGAACTTGACTCAAAGCGAAATTGA

GGAATTATGGGAGGACATTGAGAAAGTAAGATTATTTATTCTGTCCATTTCTTAGGTTGA

ATTTGTTTATCAAGTTAACTCAACCTTAGCCTATGGTATGAATTTGTTTATCAATTATAT

ACTACTTTTGTTTTTATTATTTTATTTTTATGAATTTCTTTACAGCCCTTGGAAAAGCTG

GCGGTATTAAACCTTAGTGATTGTCAAAAGTTGATCAAGACCCCTGACTTCGAGAAAGTG

CCAAACCTTGAGCAGCTAATCCTTAAAGGTTGTACAAGCTTGTCTGCGGTTCCCGACGAT

ATCAACTTGAGATCTCTCACAAATTTCATTCTTTCGGGATGTTCCAAACTCAAAAAGCTT

CCAGAGATTGGGGAGGACATGAAACAATTAAGGAAGCTTCATTTAGATGGGACAGCAATA

GAAGAGCTACCAACATCAATCAAGCATTTGACTGGCCTTACTCTGCTCAATCTTAGAGAC

TGCAAGAACCTTTTGAGTCTGCCAGACGTCATTTGTACTAGTTTGACATCACTTCAAATA

CTCAACGCCTCAGGTTGTTCCAATCTTAATGAGTTACCAGAAAATTTAGGGAGTTTAGAA

TGTCTACAGGAGCTCTATGCAAGCAGGACAGCTATACAAGAGCTACCAACATCAATCAAG

CATTTGACTGACCTTACTCTGCTCAATCTCAGAGAATGCAAGAACCTTTTGACCCTGCCA

GACGTCATTTGTACTAATTTGACATCACTTCAAATTCTCGACCTCTCAGGGTGTTCGAAT

CTTAATGAGTTACCAGAAAACTTGGGGAGTTTAGAATGTTTACAGGAGCTTTATGCTAGC

AGGACTGCTATAAGCCAAGTACCTGAAAGCATCTCTCAGCTGTCTCAGCTTGAAGAACTT

GTTTTGGATGGTTGTAGCATGCTTCAATCATTGCCACGACTTCCATTTAGTATAAGAGCT

GTAAGCGTACAGAATTGTCCTCTCCTGCAGGGAGCACATTCAAATAAAATAACAGTGTGG

CCTTCAGCTGCAGGATTTAGTTTTCTAAGTCGCCAAGGAAATAACGACATAGCTCAGGGG

TTTTGGTTACCTGATAAACATCTATTGTGGCCATTCTATCAAACATTCTTTGAGGTCATC

TCTCTCTCTCTCTCTCTCTCTCTCTCTCTCTCTCTCTCTCTCTCTCTCAAATTAACTTTT

TTTTATTTAAATTATCTTTCTTTTACTTCTATTCATAGGGTGCCATTCAGCGTGGTGAAA

TGTTTGAATATGGTTATCGATCAAATGAAATTCCAGCATGGTTGAGCCGTCGGAGTACTG

AATCCACCATAACAATCCCGCTACCTCATGATTTAGATGGTAAGAATAAATGGATAAAAC

TTGCTCTATGCTTTGTTTGTGAAGCACAGAAGGATGATAGTTTGGAAGATGAACCAGAAT

TTGTTGAGGAATTGGGATTCAAATTGAATCGCAATCACCGTATTGAACTATGTACAACTG

AAGATCCTCATGAACGTCTCCTTGAGCTTGATTACCGTGATTGCAATTGCGCCGGACCAT

TTATACATTGGTGCTTTATACCACAGAGTGACCTTGCAGAAAGTTCAAATAAACGCTTGA

TCCAAGCTACAATTACACCTGATAGCCCAGGGACAAAGGTTACAGGGTGTGGGGCGAGTC

TCATATACTTGGAAGATGTGCCCAAATTCGTCCGGAAATTGAATAAACACTATAGTTATT

GCTATCACGGCTATCAGATTGAACAAGAAGAAGATGGTATGAGAAGTATTCCGTCCACAA

GCGGGGTTCAAACTGAACAAGAAGAATTACAGGAACAAGAAACTACTACTTCAAACCGGA

TTGCAGGTCAATTGAGAAGAAACGTGGAGTCATTGCTTGAAAAACTATTTGAGGTACTCT

CTCTCTCTCTCTCTCTCTCTCTCTCACAAACACACAAAAAGAATGATGCCTGAATTACTG

ATATCATGGGTTACCATACATATCTTTGTCCAGGGATTACAGCAGGGCCTTCCGAATCTC

TATGATTATGGTTTTATTTTTTGTCTACGAGAAAGATTACTGTGGTTTAGTGAACAAAGC

AGTACACCTGCATGCACAGTAAACTTATGTCTTCCTCCAAATCTGCACAATGATGAGAAA

TGGGCAGGACTTTCTCTCTACGTTGTCTATGCTCTGCCTCCAGGTCTTCCACTTTTCCGC

ACTTTTTATGAGTGTCAGTTGTATACACCTGTTGAGGCTGTGGGCCATGAGCAACTGATG

CATCGCCTAATGTTATTATCCCCCTGGGATGACAATGTGGGGTCACATCGACTCCTCATT

ATTCATATACCACGAGTACGCTTTCCCGAACGTCTGAATCGCTGTCATTTCATTCAGGCT

TTATTTGGATGTAGAACTCCAGGTGTGGAGGTTGAAATGTGTGGGATGCGTCTAGTATAC

AACCAAGATTTGAAAGGGTTAATCCAAACAATTACCCGTTGCACCAATGATCGGCCTGCT

TACTACGGAACTGGTGATTTTACCGATACCAAGAAATATAAGGGAATTAGTTTGGGAGCT

ACAAGTTTGCTAACGAATCTGCTTGCAGCAGCCAAATCCAGCGAGCACAGCTCTGTCAGC

GAAGTTTGTCCTCCTTGCATGCCAATAGATTTCCGTCCAGAAACTTATTCTGCTGAGGAC

TCCCAACATGAAAGAAAAACAACACCAATTCGTCCACCACAGGACTTTGGCAGTTCCTTT

GATCCTACCAATAACATGAGCTCAGATCAGAATCAGCTCCTGGTAAAGATTTAATTTGAG

CCTTTGCTCTGTCTGTCTCTGTCTCTGTCTGTCTGTCTGTCTGTCTGTCTGTCTGTCTGT

CTGTCTGTCTGTCTCTCTCTCTCTCTGGATCCTGATATCTCTCTTATATTTTGCAGGACT

TTGAACGTGACTTGAAATATAATTCTTGTTTCCCTCCAAATGAGATTGTAGAGTGGTTCA

GGCATCAAAGCAGTGGCCCCTCTGTAAAAATCCCTCTACCATCAAATCTATGTGAAGACA

CCAACTGGATCGGACTAGCTTTATGTGCATACTTTTCAGTCCTTGACCACTCAACTACTG

ACCTTGACAATTTGAATCCTGAAATTTCTCACAACCTTACATGTCTATTGGAAACTGATG

AAAGTTGTCTGGAATCTCTCCATGGCTATTGCACTAACAGTCAAGAATTCGAGTGGTTGT

ATCGTATGGGAGGATTCATTTGGCTGTCCTATATACCACGATGCTGGTTTTCAGATCCGT

TGAAAGAACGAGGCCACCTGGAGGCTTCAATTGGAAGCGATCGTGGAAGCTTGGGTGTGC

ATAGGTGCGGGCTTCGTCTTATATATCTGGAAGATGAGGAAGGGCTTAAGGAGACCATAA

TGCACTGCATGACCTCCTTGTCTGATATTAATCAAGGAAAAGATAAGCAATACCAGAACT

GCGAGGTAGGATCATCTAGTATAACTGGCAGCAACGTTGTACATCCCCCTCTTGAAAGAT

CAGAGAAGCCCAATGATAAGAAATGGGTATGACTCTCTCTCTCTCTCTCTCTCTCTCTCT

CTCTCTCTCTCTCTCTCTCTCTCTCTCTATTTATATATCTGACACCTTGTCTCTCCCCTA

CAGAACTTTGGTTGTCACTTGATGTATAATTTTTGTTTCCCTTCAAGCATCACTCTGGAG

TGGTTTGGGGATCAAAGCAGTGGCTCCTCGATAAGAGTCCCACTACCACCACATTTATAT

AGTGCCACTAATTGGATAGGATTGGCTTTATGCGCATCCTTTTCAATCATGGAGAACCCA

ACGGCCGACCTAGACAATTTGAATCCAGAAATTTCTCACCACCTAATATGTCACTTGGAG

TCAGACAGAGGTACAATAGAACCTCTCCATGACTATTGTACCACCAACGAAGAATTCCAA

TGGTTGCCTTTTGGAGGATTCATTTGGGTATCTTATATACCACGAGTGTGGTTTTCAGAT

CAGTTGAATGAATGTGATGTCCTGGAGGCTTCATTTGCAAGTGATCACGAAGCATTTATT

GTGCATGAGTGTGGGCTACGTCTTGTATATCAGCATGACGAGGAAGAGATTAAGCAGACC

ATATTGCACTATATGACGTCGTTGTCAGATAAGAAAGTAAAAAATAAGCAATGCCCCACG

GGAGAAGCGGTATCATCTAGTAGGCCTAGCAGCTATATTGTGAAACCTCATCTCAAAAGA

TTAGGAAGGCCTAGTTGGGTATGACTTGCTCCCTCTCTCTCTATCTCTCTCTCTTCCCTC

CCCCCCCCCCCCCCCCCCCCCAAAAAAAAAAAAAAAAAAAAAAACACACACACACACACG

TATAACACCTTGACACCTTGTCTCTCTTTTACAGGACTTTGATCGTCACTCCATATATAA

CTCATTTACACACACACACACACACACACACACAGAGATATATATAAAATACATTGATAC

CTTGTCTCTCTTTTACAGGACTTTGATCGTCACTCCATATATAACTCATGTTTCCCTTCA

AGCATAACTCTAGAGTGGTTTGACCGTCAAAGCAATGACTCCTCGGCAACAATCTTGCTA

CCACATAATCTAAACTTAGACAGCAATTGGATAGGATTAGCTGTATGTGCATACTTTTCA

GTCCTGGAGCATCCAACTGTCGACATTGACAATTTGGATATTCCAGCAATTTCTCACCAC

CTTATATGTAATTTGGAATCAGATAGAGACAGTCTAGAATCTTTGCATGACTACTGCACC

ACAAACGAAGAATTCCTATGGTTGCATTTCGGAGGATTCGTTTGGGTATCCTATATACCA

CGAGCATGGTTTTCAGATCAGTTGAATGAGTGTGGTGTACTAGAGGCTTCAATTGCAAGC

GATCATGAAGCCTTTAGTGTGCAAAAGTGCGGGCTCCGTCTTGTCTATCAGCACGACGAG

GAAGAGTTTAAGCAGACCATATCTCGTTCTCAGATAAGAAAGGGAAAAATAAGCAACACC

CTCACCGCCCGTTGAACAGGATCAATGACACTAAGGGTAACTAAATACTGGAATAACTTC

TCCTCATGTATTTGGTTAGCAGATTTTGCTACCTTGGGAACTCCTTCTCAATATATTGAT

GAAGTCAAGAACTCATCTAGTTCTTAGTCGAAGTCCATTACGAAAGTCATAATCTTTTAC

TGGTTGAATGAATTACAGAGTGGTTAGCTTTTTTTCTATTAAAAAAAGTGGGTTGATTAA

GTATTTGATCATTATATGCGTTTCCACAGCTTAATTGGGTAATTTAGGTTAGTTACTAAA

TGCTGTTCTTCTTTTGAAGCAAAAACAGGGCAAAATAAATACGAATTCATACAAATCAAA

ATGCATATGACAGTGGGAACCAAAAGATATTCAATAAATAAAAAGATCAAATTTTGACAT

ACCGGATGTCGGCGAGCTGAAGATTGAGGACGCCCATGATTATTTCTTCAACCCTCGAAA

TTTGTCTGCTACAGAAGCTATGGCTTCCATTGAAGACCTCTATCAAGTATCAGGACAAAC

GGAGCCAGAGAAGGAGGGTCCTTCAATCCATTGGCAAAGATTGCTGCACGTGACCTCAGG

ATTTTATACATTCAAATTATCTCTTTCAGCTGAGTTAACTGGAAATTGGGCAACCCGGGA

AACATTGTTCCTCTGAATTCTTCTTTTCTGTTTGGGAAAAAATTGAAGTAATGAACAACA

ACTAGACCTGGCAACTTGTTTTAACTCGTCAAATTGGACTAATTAGTCAAGTTAAGTTTT

GGTGTTCAGTGTTCAGGTTTTATTTTCGCTCTTGTTTTTTCAAAACTGAAAACCGAAAAC

ATATTTGACAATAGATTTCGTTTTCAGTTTTTAAAAAAGTTATCAGTTTTTAAAATCACC

CTTCTTTTTTTAAACAACTTGAAAACGTTTTGATTTTTGATTTATTATATTATTATTATA

AATTATAAATTATTATATTTCTTTTTGCGTTTCTTTGTCATTCACCAAATTTACATCACT

ATACATGACAGGACCCGACCCAATTTCTGCTTTGGAATCCGAGCCGAATCCTGTGCGTGT

CCGACACCTGGCGAATGTCGGGCACGAATGACCTTTTTACCCTTTCCTTTTTCAATTTCT

TTTTAAAATTTTCCTCAGACTTCTGCCGAAAATTCTGCAGAGTCTCCCCTGTATTTTGAC

CAAACCCAAAATCTTCAACCTGTCAAACAGTCTCAAACAATACACCAACTGCCAGACTAA

TTCAACAACCAGATCTCAACTTCAGTTTCTTATGTTCAACAAGATATCAGAGCCTTTCTA

TACAAATTTACAAACTAAGAGAAGTTACAACGAGATCCTACGCTTCGGTGGTGGGGCGGG

AGCTTAGATGATGGCTCGGGTGGTTTCCTTCGTTCTTCTACGGCCTGGGGGCGAAAAACA

AGTTAAAATTGTGAGTGGACAAAAATAATGTTCATAAAAACATTTGAGAACATAATAACC

CCCGTTTGAAAAACATAGGGATATAAGAACTACTATTTGATCATAATCAAACTCGAGACA

TTCAATTAAACATAGATAATCATAACTATATAAATGTTCACATACTAAACAAAAATATAT

CAGTATAACACATGTTTGAGTATCGAATATACTGATAAAAATGAGTGAAACAATAACTGC

ATTAAAAAGTTTTAAAAAGAATCCTTTAGAACTGCCACCTAATTGTACCCCTGTGATAAC

CGTCAATTCCCTGGCAGGTCTCGGGCGTCACACAGGCTACCCGAGCTGCAAACTGGCGAA

ATCAGGGGACTATGATCAGCCTGATCCGCCGGCAGGTCCTCGATGACACCAAGTCAGCTC

GAGTCGCTCAGGCAGGATATAGGGGACCGTAGTCAGCCTGATCCGCTATCCTGGCAGGTC

TTGGGGACACAGAGTCAGCCGAGCCGCAATCCTGGCAGGTCTCGGGACACCAAGTCTGCC

GAGCCGCAAATCCTGGCACTCACGGTCCGAGCGTCCCCGTAACTCGTGAGGCAAAGTCAA

GTGCACTGACTGAACTATAATCAGACTGGATGTCCGTAGACATCGGTCCGACTCTGGGTA

ATCACCAAAAGTAATGGGTACACGTTGGTTTTCTTTGAAAAATAAATTTAGAAAAACCTG

ATAATTCTCTGTAATCTGATAAATCTGATAAATCAGAGTTCAACTGTAAATTTTGTATCA

TAAACCTCAAATCTGCTGCTCAACTGAGTAATATAAAAGTAAAGACATCTTACGCTACGA

TTCATGGTTGGAACACTTGCTATAATACTTATTCAAGATCAACTACGAGAGTATATTCAG

ATAACCTTATTTATATAAAATCAATATAAAATCACTTTAGAATTCTTATTTATAAGAAAT

TTTCTATATAAATTATTTATATAAAATCATTCATGAAAGAAAGTCCACTCACAGCTGGTC

CGAGTTAGCTGGACCCTTCGAAGGTCCCTCCTGTGGTTTAGCTGGACTCCTGGTGCCTCA

CTCGAAAGATTTGGTTGAGAAACGAGAGAGAAAATCAAACTGGAAGTTTGATCACAATAG

TCGGTGAAAATGGCCGATTCCGGCCAATATCCGGCGAGCTGGGGTCGGCGACTGGTCACG

GGAGGAAGGCGGCGACGAGGCGAGTCGAACGGCACCAACGGTGGAGGTCGCCGTGCCCTG

TGGCGGCGGCGCGACGAAGCTGAAGGGGGAGGGGCGGCTGACTCGCGACGGGAGAGGGAG

AGAGAGAGAGGAGAGAGAAATGAGGAGAGAGAAGAGAGAAGAAAGAGATAAAATCTGACT

TTTTCCAAAGTAGCGTTTTGCCCTTCGCGATATTTTGATCGTAATTTCTTCGTTAGAACT

CCGATTTGGGTCTACTCCGTGTCTACGGACTCGTTTCGCCGTGCACTACGCAACGGCGCA

AGAGGAATTCTCAAATTCTTTTCTGATCAAAAAGTCAAATTTTTCCCCATTAAAAAATGC

GAGGGCAGAATCGTCTTTTGGCTAAAATAAAAAAATCTTAATTTTTAGATATTTTGGTTT

GGGTTCTTACAATACATGCATTGCACTTCAGTTAACAAAACTCGATATTTGGAACCTCTC

GACTTGGCCCGACTGCTGGACTCTTGTATTCAGCTGCAGACCCAATTCAAGCCTCAGACC

TCTAAATCCAAAATTCTCCACAAATTCATCATAACTCCAAAAAAAAAATTGATCGAAAAT

GCTATAAACTCTTTGTAACATGCACTTTCATTCTATGATCTCAAATTTCCCCAGTGACAT

GAGTTAGCCGAAAAGTTGAAACGATCTAAAGAGAATCACTCTTGAAAATAACTTTTTTGT

AATCCCTTTCTATTGCCATAGTTTCCCCGAATGGGTGTTAACCGACATGACTCTCCTAAG

AGCACCCATCAAACTCAACCAATTCTGGAATTATGAGTGAAAAACACAGACAACTCATAG

AGTTTTTGGACTTTATTGACCAAAAAAAGACCAAGAATAAAAATATAAAACGCTAATAAA

CGGAAGAATAAACATCTCTAATCTTCCTTCGAGACATGACATAGAGAGTGCCACCTAGTC

ACTTCATACCCCTAACACACCAATCAACAATAGATCTACCATGATAACATCCCCCTACGC

GCACACATCCAAAGCCAGTCCAGATTCGTGGCACGAACAATAGATCTGTCAAGAAAGACC

ATATCGACATAGATCCGCCAAAAATTGAAGAACACTGAGAATTGGGGGGATACCCCCCAA

ATCTTAATCTCTCTCTCTTTGTGGGCTATGGTTTTTTTTTGTCTAAAAAAAATACAACTT

TTGAATGTGTTGGGATATAGCGAAACTGAAAAAGATAGTTCCTCGTCATATTCCTCCAAT

GAAATCCTTCCCATATGGAATTGGAGGGAAATTGCTCTCATATGTACGCATTTTCATATC

TCTCTTTAATAAATATATGGTATGGGCATGTGGACCTGCCTCTTGAGAAGTGGCACGAGT

CCATCGTTCACACTATAATTTTTTATCTTTCTTTGGTCTCAGGTTCCACGCTATCTTTTT

ACACCAGAAAATCAAACAAGTTGGCATAAAATAAGAGTGAGAGATCCCCCTTAAAATAAG

ATGACTGAAGCCTCCTGCTCTTTTGGCTGTTTGTCTATAGTATTCCCAATCTTTTGATTA

AAAGGAAGCTAATCTTGACTCAGACTAATTAATCTCTGATTATTTAGTGTCCTGTTCGTT

CAAGCATTATATTGCACTCAGCTACCAAGCTAGTTTTGGTTCTGCAAATAATTTTAAGTA

GCCAAAGCTTCCATTGCCAGGTAGCAGGTTGGTTACTGATTTAGTTTTTCTCCAATGCCC

GATCCCATGACCACCCAAACCTCCTTAGCTTTGCCTCCTTTCTCGACCCCTCCACCGAAA

TATGAGGTGTTCCTCAGTTTTAGAGGTTTCGACACTCGAAAAGGTTTTACTGACAATTTG

TACAAGGCCTTGATTCACTACGGAATCCACACTTTCATGGATGCTGAACAGCTCGAAAGT

GGAGAACCCGTTTCAACGGAACTCTTCAAAGCAACTGAAGAATCGCAAATATCAGTCATC

ATTCTTTCAACAAACTATGCAACCTCCACATGGTGTCTGAATGAACTTAACACTATGGTT

GAACTTGCGGAAAACGACGAGTCCAGACTGATTTTGCCTGTGTTCTATGGCGTGACGCCA

TCCGAAGCACGAAAACAGATTGGAGTTCATTTTGAAGAGGGGTTTGCTCAACATAAAAAA

GATTTCGGAGGAGAGCCAGGAGAGGTGGCAAGGTGGAAGAAATCTCTCACTGCAATCGCC

AACCTCTCGGGATATGATATAAGAAATTACAGGTAAGGCTGCTGCTTTTTATTTTGTATT

TTCTTTTTTGGGGTAAACATTTCAGTTATATCTTTTTCATAGTAGATGTTGGTCAAAACT

ATCTCCTTTGATCAAATACGGATCCTCGGATCACTTTCCACAGTCATATTCCAAGAAAAT

TAAAAAACAAAAGCAACAGAAGAAACAGATAGATCGAAAACATGCTGTATTGTTTTTGGA

TACATGCTAAGTGTTTGTAGAATTATGAAATGGCGAAGAACTACATCCGTATAAACTGAA

TTTATCATGTTTCATAAATTCGCTATCTGCTAGATGTCTATAAAAACCTGCCAACCTTTA

TGTCAAAAGTCCAAAACATTCTATCACTCAAGTAAAAACTATGTAGTAGTCCATACAAAT

CTAATCGTAAACACCTTTTGCTTAAAGCACAGGTGAAGTCCGCATAACATTAGAAGGGTT

CAGGTTGAGAGGCCGGAAAACTAGAAAACTTGATAAGTTTTACATGTTTTCGTCTTTAGT

TATTACTTGGATTGACCAAACACTTGACATAAAGCTGTAACCTTTTTGCAGAAACGAGAC

AATGGTGATTGAAAAGATAGTTGAACGTATTTTTGGTGTTTTGATTAACACATTCTCAAA

TGATTTAAAGGACTTTGTTGGTATGGATCGTGTAAACAAAATTAAATCCAACATGAGTCG

TATAGGGACTGAGGAAGTTCGTGTAATAGGGATTTGTGGGATGCCAGGGATTGGTAAGAG

TACCATTGCAAAAGCTCTTTCTCAAAGAATTCGCAATCAATTCGACGCTTTCAGTTTCAT

TTCTAAGGTTGGAGAGATATCTAGAAAAAAAAGTTTATTTCATATCAAAGAACAACTTTG

TGATCACTTGTTGAATAAGCAAGTGACTACAAAGAATGTTGATGATGTGATATGTAAGAG

ATTGTGCAACAAACGGGTGCTTATAGTTCTCGACAACGTGGAGGAATTTGAACAAATAGA

TGCTGTAGCTGGAAAAGATGGTGCAGAATTATCTAGTCGGTTTGGTAAGGGTAGTAAAAT

CATCATAACCACAGCATATGAAAGGTTGTTGATAAATTATAATCCAAAAATATACACAAT

AGAGAAACTTACTCAAGATGAATCTCTTCTTCTCTTCTGCCGGAAAGCATTCAAAAAAGA

CCATCCTATGGATGGTTATCAGAAGTTGTGTTATGAATTTCTGGATTACGCTGATGGTCT

TCCTCTGGCTCTTGAAGTTTTCGGTAACTCCTTACTGAACAGAAGTGTAGAAGATTGGTC

TAGTAGATTGGCTTCTTTAAAAGATGATAACTACTCTGGTAAGAACAAAATTTTTAATTA

TCTTAAAGCAAGTTTTGATGGATTAGAGAATCAAGAACAGCGGGAAATATTTTTGGACAT

TGCGTGTTTCTTCAAAGGCGAGGATGCATGCCGTGTGGAAAAGATATTTGAAAGTTGTGG

CTACTATCCAGGCATCAATATAAACATCCTCTGTGAAAAATATTTAGTGAGTATCGTAGG

AGGAAAATTGTGGATGCACAATTTACTACAACAAATGGGCAGAGAAGTTGTTCGTGGAGA

ATCCAAAAGGGAAGGAGAACGCAGCAGATTGTGGCTTCATACAGATGCCATTAATGTACT

GAAGGGAAATAAGGTGAGAAGTTTAGTAGAATCATACACTACTCTCTAATATTTAATGAA

ATTGAGAAGCCAGTTTGCTTTCCAATGCCTATGTTTCAATTTACATTTTTCTATTATGGA

TATTAGGGGACAGATGATGTTCAAGGTATTTTCCTAAGCTTGCCTCATCCAGACAAAGTA

CACTTGAAGAAAGACCCATTCTCAAACATGGACAATCTAAGATTGTTGAAGATTTACAAT

GTGGAATTTTCTGGATGCCTTGAATATCTTTCAGATGAGTTGAGCTTCTTGGAATGGCAC

AAATATCCTTTAAAATCTCTGCCTTCAAGTTTTGAACCCGATAAACTTGTTGAACTGAAC

TTGTCTGAAAGCGAAATAGAGCAATTATGGGAGGAAATGGAGAGGGTAAGATTACTTTGT

CCATTTCATAAGTTGAATTTGTTTATCAAGTTAAGTCTTTCAGAGAAAGCTTTTTATGTT

AAATTCTTTGCAGCCTTTGGAAAAGTTGTTGATACTCAACCTAAGTGATTGTCAAAAGTT

GATCAAGATCCCTGACTTCGACAAAGTCCCGAACCTTGAGCAGCTAATCCTTAAAGGTTG

TACAAGCTTGTTTGAGGTTCCTGACATTATCAACTTAAGATCTCTCACAAATTTCATTCT

TTCGGGATGTTCCAAACTCGAAAAGCTTCCAGAGATTGGTGAAGACATGAAACAATTAAG

GAAACTTCATTTAGATGGGACAGCAATAGAAGAGCTACCAACATCAATCAAGCATTTGAG

TGGCCTTACTCTGCTCAATCTCAGAGACTGCAAGAATCTTCTTAGCCTCCCAGACGTCCT

TTGTGCTAGTTTGAGATCACTTCAAGTTCTCAACCTGTCAGGGTGTTCGAATCTCGACAA

GCTGCCAGAGAACTTCGAGAGTTTAGAATGTTTACAGGAGCTTGATGCAAGCAGGACAGC

TATACAAAAGCTACCAACATCAATCAAGCATTTGACCAGCCTTACCCTCTCTAAGCTGAA

AGACTGCAAGAACCTTTTGTGTCTTCCAGACGTCATTTGCAATTTGACATCACTTCAAAT

TCTCAACGTCTCAGGGTGTTCAAATCTTAACGAGTTACCCGAGAACTTGGGGAGTTTAGA

ATGTTTACAGGTGCTTGATGCTAGCGGCACTGCTACAAGCCAAACCTGAAAGCATCTCTC

AACTTTGTTAGCTTGAAGAACTTGCATTGAATGATTGTAACAAGCTTCAATCATTGCCAC

GGCTTCCATTTAGTATAAGAGCCGTAAGTGTACATAATTGTCCTCTGCTGCCGGAAGCAC

ATAATCTCCAAAGAAATTGTAAAGTGAAAAATGGAGAATATTTTCTCTGTTGAATGAATA

AATCATACAGGACAATTCAAGGAATATAAAAGATACACGAGGAGCTATTCTAAGTAACTA

ACTAATCTAGGCTAGAAATCTGGTATGACCCACACAACGTGGTGTGCACTACAAGTAAAT

ACATAAATACAGAAGTTAAGGGATTTCTTTCCCAACACTCCCCCTCAAGTTGGAGAATGA

ATGTTGCGAAGTCCCAACTTACTGCTCATTAACTGGAAGTTATTTCTTCCCAATGCCTTC

GTAAAAATATCAGCTAGTTGAGCACGTGAAGGAACATGTGAAGTAAAAATGAAGCCAGCT

AGTAATTTTTCACGAACTATGTGACAATCAATCTCAATATGCTTTGTTTGTCATGAAACA

CAGGGTTTGCGGCAATGTATAATGCAGCCTGATTGTCACAGTGTAATGGCGCAGGCTCAT

CTTGTGACACCTGCAAATCTTGTAGAATATAATGCAACCAAGTTAATTCCAAACAAGTTG

TAGCCATTGCGCGAGCTGCCTCTGCGGATGATAATGACATTAGTTTGTTTCTTGGATTTC

CATGAAGCTAGAGAGTCTCCCAGAAAAATATAGTATCCTGTGACAGACTGCCTGGTGGTA

GGGCATCCTACCCAATTAGAGTCACAGAAAGCTTTCAACTTAAGATTACTGGAAGAAGGG

AAGAGCAAACCTTGGCTAGGTGTGCCTTTAACAAATCTCAAAATTCGGAGTGCAGCATCT

AAGTGTGGTTTTCTTGGCTGATGCATAAACTGGCTCAAAGTCTACACTGAATATACCAAG

TCTGGCCTGGTTACTGTAAGATAGAATAATTTCCCAACTAGTCTCCTGTATCTAGTAGGA

TCATTAAGCAAATCACCTTATGTAGGTGAGAGTTTCAAATTCTGCTCCATAGGAAAGGTT

TCAAATCGTGCTCCCAATATGCCTGCATCTTGCAATATATCCGAGGCAAATTTCCGTTGA

GACATAAAGATGCCTTTTTGAGAACGTGACACTTCAATGCCAAAAAAGTATTTCAGATTA

CCAAGATCCTTGATACGGAATTGACGCAAGAGAGAATATTTGAGAAGTTGGATTTCTTGT

GCATCATTCCCTGTACCAAGTATGTCATCGACATAAATCAATATGCCAACAAATGAAGTA

CCTTTTACTTTGGTGAAAAGTGAATAATCTGCCTTGGATTGTAGAAAACCTACGTTCTGA

ATAACAGTGTGGAAAATTTAGAGAACCAATTTCGAGAAGCTTGTTCGAGGCCATACAGTG

ACTTGTTAAGTCGACATATCAAGTTCTCCCCTTATCGGCAAAGACCTGGAGGAGGAACCA

TATAAACATCTTTCGTCAAGATCGCCATGAAGGAAGGCATTTTGGACATCCAACTGATGG

AGAAACCAATTACGAGAGCGGCAATGGCTAGTAAGCAGCGGAGAGTAGTGAGTTTGGCAG

TGGGAGAAAAGGTTTCAAGATAATCCACACCTTCAATTTGAGTATAACCTTTGGCAACGA

GACGCATTTTGTAGCATTCAATGGTCCCATCAGAGTTATATTTAATACGATAAACCCATT

TGCAACCGATGGGTTTGTAACAAGTAGGAAGGGGCACAAGGTGTAATAACCCAAACCAAA

ATATCTAAAAATAAAGAAAATATCTAAAAAGTAAGGAAAATATCTTCTAGCGAAAAGACT

ATTTTGCCCTCGCATTATTTAATAGGGGGAAAATTGACTTTTTTATCAAGAAAGAATTTG

GGAATGCCGCTTGCGCCATTGCGTAGAGCGCGGCGAAACGAGTTCGTAGACACGGAGTAG

ACCCAAATCGGAGCCATAACGAAGAAGATATGGTCTAAAAACTGCGAAGGGCAAAACGAT

AAATTGGCCAAAAAGTCAGACTTTTTATCTCTCTCTCTCTCTCTCTTCTCCCCCGTCACT

TTCTCTCTCTTCCCTCTCTCTCTCCCTCGCGTCGCACCCAGCCGTGCGCCCGTTCGACTT

CCAGGCGACGGCCCGGCCACCACAGGCCGAGCCGATCTCCGCCGTGGGTACCATCGGGTC

CGCCTCCGTCTCGCCGTCAAGACCTGACCGACCTCCACCCCGGGGCCGCCCTGAATTGGC

CGAAAAATCATGTTTTCCGACGGAGGTTCCTTCGAAGCCACCCGAACTTCCAGCTCAAAA

TTCTCCTTCGTTTCTCCACCAAATCGATCGAGTAAGGCACCAGGAGCCCGACAGGATTGC

AAAAGAGACCTTCGAAGGGTCAAACTAGCTCGGACCATCTGTGAGTGGACTTTCTTTCAT

GAAATATTTTATATAAATGAGTTATATAGAGGTTTTCCATAAATAAGATTTTATAAGTGA

TTTTACATTGATTTTATATAAAAGAGTTTTTATAAACGAGTTTATTTGACTAAATTCCTT

ATTTGATCTTGAGTAAGCTTTATTGTGTTTCCGAGCATGATTAATACAGAAATAGTTCTA

TAAGTTGTGTGCTGCTTACTTTTACATGCTTAAGAGTTATAGAAAACAGATTTTGAGGCT

TATGACAGAAGAAATAGCAGCACAACTTGAGATTTCAGTTATTATTCAGATTTTTTCTAA

GGAATTTATTTTAAAACTACCTCGTACCCATTTATTTTTTGGTGATTACCAAGAGTTGGA

CCGATGTCTACGGACATCCAGTCCGATTTCAGTTTATGTCAGTGCACTTGACTTTGCCTC

ACGAGTTTCAGGGACGCTCGGACCGTGAGTGCCAGGATTTGCGGCTCGGCAGACTTGGTG

TCCCGAGACCTGCCAGGATTGCGGCTCGGCTGACTCTGTGTCCCCGAGACCTGCTAGGAT

TGCGAATCAGGTGACTACGGTCCCCTGCATACTGCCAGAGCGACTCGAGCTGACTTGGTG

TCATGGAGGAATCTGCCAGCGGGACAGGCTGATCATAGTCCCCTGATTTCGCCAGTTTGC

GGCTCGGGTAGCCTGCGTGACGCCCGAGACTTGCCAGGGAATTGACGGATAAGACAGGGG

TACAATTAGGTGGTAATTTTTAAAGAATTTTAAGCTCTTTTATACAGTTATGACTTTCAG

CTATTTTATACTAGCTTCTCTGATTTTTCAGGCATTGATACATTTATATAGTTTTGTTCA

GTTATACAAGGTTTGAGTACAGTACTTTTATACAAGTTTGGTTTCAGTGTTTTATACAAG

CTTTGATTTCAGTGCTTGTGAATAAAATTTATTGAGCTTGAATATGATTTACATAGAATG

CTTGAGTTTAGTATGCTTTAAATGAAGAGTACGTATTCAGCTTATTTAACAATTTTTTTA

TAGTGGGGGTTATTATGATTGTTAAACTTTATTTCAAAAATTCTCATGTTTGGTCCACTC

ACATCTTCAAACTGTTTTCACCCCCAGGCCGTAGAAGTACGCGGGATCCACCACCGGGCC

AATCATAGCCTCCGCGCCAAGGTAGAGTTTTGTAGAAAATCCTGGAAACCTTAAAAACTT

TAGAGTATGCTCTGATATCTAGTATTAGTGGAAAAATGGAGTTGAGATCTGTTACTTGCG

ATATTTTGGCTGTTGGGATAGATGTACTGATTGTTAACAGGTGGAAAATTTTGGGATTGG

TCAAAAGACAGGGGAGACTCTGCCGAAATTTCGGCAGAAGTCTAAGGGAATTTTAAAGAA

AATTTGAAGCAAGAAGGGCAAAAAGGTCTTTTGTGCCTGACATTCGCCAGGTGTCGGACA

CGCACCGGACTTGGCTGGAATTCCAAAGCGGAAATTGGGTCGGGTCCTGTTAATTTGGTA

TCAAAGCATAGGTTTTACTTCCTGTAGACTTCGCACTCTGTGCATCCTCGTTCTCTTCTC

AAGTTGGGCCCAAATGGTGGTGGTATCCGTAGAAAAAATTAGATAGTTATCCCGACGTAA

GGCGATGAAACTTCCAAGTCAGACAGGAACTGCACCGGTATCTGAACCGGCAGAGTAATC

TTTTGTAAGAGGTTTGTAAGGAGCCGTACCTACTGTACCTAGTAGGCAGGGAGATCCTAA

CACTCAGTAGACCCTGGAGATGTTAGCTCAGGCTATAATCAGCCAGAGATCCATCCGCGA

GATAAGGGGATCACGTAAACAGGAGTAAGAGCTATAAATTGGAATAATAATGATATCTAG

TAGTAAAAGAGGATTGAGTTGAAGGAATATAGCAGGTCATAGAGATCCTGGTTGTTCCAC

AGGAGAATAAAGTTTTGGGTAGGATTTGATTAAGAAGTGATGTTAAAATCCTCTGACTCG

TACGGAGTAAGCATTGTGGGAGCAGAATATGATAGTCAATCTTATTTATAAGTCTACAGA

AGGTGTGAATACAGGAGTTTTTGCAGCAGAATAGAGGATAATGGTTAAACCTGAATACGA

GAAAAAGGTTTCGCGTACGGTTTTTATTAATAGAGATTGGAAAGTAAGAGGTAGTACCCA

GTTACGATCAGGTTAAAAGCCTCCATTCAAGGTATAGTGATCAGTTAGTAGTTAGTCAAT

TTTGAGGCCGTGATGATGTCTGCCCCAGCCAAATAATGGGCAGACCATGGGGTGCACTTT

GTGCACAGTATTTGGAAATAGATAACCTTAACCAGGAAGAGTTTAAGAGAGATACTACAG

TGGTAATCACAGTTTCCAATCATTGAACGGCCGCATGCTTGGAGGCCATCGAACTGGAGT

TAGTTCTGGGGCCGGTGTAGCCAAAAACATAGAGAGACAGGTGTTGATAGGCTATAACAG

GAGAAGTTGCTCTAATGTGCCAGATGTGGTAGGTACCGTTCTGGGCCCTGCTAATGGAAT

ACCAGTCTCGATAGTTTAGAATTAGAAATCCAGGTTAGGTTTCAATTGGACTCAGGTAAA

AGAAGCTTTTGGGCCTTGATTTCAGTTTAATACCGCAATACACTCTAGACAGACGACCAG

TTTAAGAAAATGACTCAAATTGTGGGAAGGTTATTGGGACATAATGTACTACAGTTTCAG

AACGATTTGAGTAAAAAGATGTTGGTTTGAGAGTATGCCTTAAGTAACAGTTGTTAGATT

TCCTTGGATTAGATGTTTTCAATAGATGTCTTACTCGAGATATAGTATGGATTACCCAAG

TATGAAGATAGAATGTTAAGTAAGGTTCATTGAAATCTGGAAGTATTGTAGGAAATAAAG

AACCTGATTGAATTAAGCAGAAAAAGACTCTAAGTAATCCAGGATTGGCAAGCAGTTATG

CAGATACTAGAAGAAGGATCTTCATATAAGTAGTCTCGATAGTTATCGCCTGAGGAAGGT

GAAATGAGAATCAAGGAAGCAAGGGAGCCAAATTTTAGCCGTTCCGAAACCAATAAGATT

ATAGAATGTGTAGGAAGCAAGCAGTTCAGAGGATTGAAAGGGAAAGTGTTTAGCTTTCAG

TGACATATGCAGTATAGTAGCCCGAGTAGCAGATACAGGAACAGTTCTTCTCCCCCTCCT

GGAATGACAGAAGTGTAAATTTCGAGGACGAAATTTTTTTAAGGGGGGTAGATTGTAATA

ACCCAAACCAAAATATCTAAAAATAAAGAAAATATATAAAAAGTAAGGAAAATATCTTCT

AGCGAAAAGACTATTTTGCCCTCGCATTATTTAATAGGGGGAAAATTAACTTTTTTATCG

AGAAAGAATTTGGGAATGCCGCTTGCGCATTGCGTAGAGCGCGGCGAAACGAGTTCGTAG

ACACGGAGTAGACCCAAATCGGAGCCATAACGAAGAAGATATGGTCTAAAAACTGCGAAG

GGCAAAACGGTAAATTGGCCAAAAAGTCAGACTTTTTATCTCTCTCTCTCTCTCTCTCTC

TCTCTCTCTTCTCCCTCGTCACTTTCTCTCTCTTCCCTCTCTCTCTCTTTCCTTCGCGTC

GCACCCAGCCGTGCGCCCGTTCGACTTTCAGGCGACGGCCCGGCCACCACAGGCCGAGCC

GATCTCCGCCGTGGGTACCATCGGGTCCGCCTCCGTCAAGACCTGACCGACCTCCACCCC

GGGGCCGCCCTGAATTGGCCGAAAAATCATGTTTTCCGACGGAGGTTCCTTCGAAGCCAC

CCGAACTTCCAGCTCAAAATTCTCCTTCGTTTCTCCACCAAATCGATCGAGTAAGGCACC

AGGAGCCCGACAGGATTGCAAAAGAGACCTTCGAAGGGTCAAAGTAGCTCGGTCCATCTG

TGAGTGGACTTTCTTTCATGAAATATTTTATATAAATGAGTTATATAGAGGTTTTCCATA

AATAAGATTTTATAAGTGATTTTACATTGATTTTATATAAAAGAGTTTTTATAAACGAGT

TTATTTGACTAAATTCCTTATTTGATCTTGAGTAAGCTTTATTGTGTTTCCGAGCATGAT

TAATACAGAAATAGTTCTATAAGTTGTGTGCTGCGTACTTTTACATGCTTAAGAGTTATA

GAAAACAGATTTTGAGGCTTATGACAGAAGAAATAGCAGCACAACTTGAGATTTCAGTTA

TTATTCAGATTTTTTCTAAGGAATTTATTTTAAAACCAGCTCGTACCCATTTATTTTTTG

GTGATTACCAAGAGTTGGACCGATGTCTACGGACATCCAGTCCGATTTCAGTTTATGTCA

GTGCACTTGACTTTGCCTCACGAGTTTCAGGGACGCTCCGAACGTGAGTGCCAGGATTTG

TGGCTCGGCAGACTTGGTGTCCCGAGACCTGCCAGGATTGCGGCTCGGCTGACTCTGTGT

CCCTGAGACCTGCCAGGATTGCGGATCAGGCTGACTACGATCCCCTGCATCCTGCCAGAG

CGACTCGAGCTGACTTGGTGTCATCGAGGAATCTGCCGGCGGGACAGGCTGATCATAGTC

CCCTGATTTCGCCAGTTTGCGGCTCGGGTAGCCTGCGTGACGCTCGAGACTTGCCAGGGA

ATTGACGGATAAGACAGGGGTACAATTAGGTGGTAATTTTTAAAGAATTTTAAGCTCTTT

TATACAGTTATGACTTTCAGCTATTTTATACTAGCTTCTCTGATTTTTCAGGCATTGATA

CATTTATATAGTTTTGTTCAGTTATACAAGGTTTGAGTACAGTACTTGTATACAAGTTTG

TTTTCAGTGTTTTATACAAGCTTTGATTTCAGTGCTTGTGAATAAAATTTATTGAGCTTG

AATATGATTTACATAGAATGCTTGAGTTTAGTATGCTTTAAATGAAGAGTACGTATTCAG

CTTATTTAACAATTTTTTTATAGTGGGGGTTATTATGATTGTTAAACTGTTTTGAAAAAT

TCTCATGTTTGGTCCACTCACATCTTCAAACTGTTTTCACCCCCAGGCCGTAGAAGTACG

CGGGATCCACCACTGGGCCAATCATAGCCTCCGCGCCAAGGTAGAGTTTTGTAGAAAATC

CTGGAAACCTTAAAAAATTTAGAGTATGCTCTGATATCTAGTATTAGTGGAAAAATGGAG

TTGAGATCTGTTACTTGCGATATTCTGGCTGTTGGGATAGATGTACTGATTGTTAACAGG

TGGAAAATTTTGGGATTGGTCAAAATACAAGGGAGACGCTGCCGAAATTAGACAGAAGTC

TAAGGGAATTTTAAAGAAAATTTGAAGCAAGAAGGGCAAAAAGGTCTTTTGTGCCCGACA

TTCGCCAGGTGTCGGACACGCACCGGACTTGGCTGGAATTCCAAAGCGGAAATTGGGTCG

GGTCCTGTCACAAGGGACCAAGTATTATTGAGGGCAAGGGCATCCAACTTAGCCTGTATG

GCAGCACACCATTAAGGATATGAAATTGCTTGATCATAGGTGGAAGGTTCAATGTGACCT

GAAATGTGAGGTAAAAAAGAACGATGGGATGGGGAAAAATGAGAGTAAGAAAGAAAGTTA

GAAAGAGGATATTTTGTACTGATAGGATTTTTAGTACCTGTGCAAACAGGGTTAAAATCA

CATAAAATACTTGCAAGAATACAAGGCTATTGTAGTATAAATGCTCATGCAAGGTCGTTG

TCCCCAAAGATTGTTTGACTAATTTGTAATTAAACTAACTCTTAATTAATAACTAAAATA

GATTTTATCAAAGATTTGAAGTTTGTGAATTTGAAAAGATTAAATTAACAGTAAAAGAAA

TCTGAAAGTTGGGAAATAATAACAGCAAAGAAGAAAAACGTTTTCTTTTTAAAGACTAAC

AATTTAGAGAAAACTAGGGCTTAGCCATTACCGTCACAATCCTATGCAATTCTATCAATT

ACTTATGAAGTTCCACATATATGCTTTGAAGGTTAGGTTTTCCTAATGCATATTTTCCTT

GCGATGTTCAAGCGAAAACGTATATCTAACATGCAATCCGTCTGTGATGTTCAGATCAAA

TATAAACATGCAAAACTCATTAAGCTTTGTGAAAACCCTTTGAAAAATCATGCAATCCTT

AAGAGCGTGATGTTCGCATTAAGTGAACTTACAACTACTAATCACAAGAAGCCATCCTCA

ATTTCAGGGTGATGTTCCAATAGAAATTGCATCAAATTACTTGTCTACATATCCTAATGG

TGATTAGTCATTAAAATAGATAGATAGTTTTAAATGCAATGATTAATTATTCAAAGCAAA

CATGCATACATTCATAAGCAAATTAATAAAATTACATATTCATGCTAAGGCTTATGGCCT

CGCCCTAGCAATTTGAATTAGTTACACATACTCATAATTAAAATAAAAGAAAATATCATT

AGAATAAATAGGAATAAAAACACCTTGAAGTAGAAGCTCTTCAAAAACCCTAGCCAAAGT

CTCTGTTTTTCCCATAATTGCATAAAAGGAAACTAATTCAAAAACCCTAAACCACAAGGA

AACTAATTTAAAATAAAATAATACTAGGATATAAAATCCAAATTGAATTAGGAGAATCTG

CCCACAATCTGCCCAGCCACGTTTTGGCCTCAAATCTCCTCAAAATCTGACCCAAGATAG

CTTGTTTTAAAGATAAAAATATTCTGAACACTTCTCCAGAAGGTTACGAACCCATCCAAG

GTCATCTTGGGCTCCAAAAACACAATAAAAGTCCAAAATGTCACTATTCCGGCACCGCGC

ACTACTTCTTTATTTTATTTCCAGAAATTAACAGCTTGGTAGAAAAATCTGAAATGGTGA

TACGATCAAGCTAAGTGACTCACATACGTCCTCCAACTGGAATTACTCCAAAATTCGTCC

ATTTGATTACGTTTTGCTCTAGAGAGAGTCGAAAGTCCTATATTGAAAATATAATTCAAA

GTATCAAAGTTCTTCTAAAATATTAACCAAAATGTATTAAGAATGAGGTTAAATATATAA

TATAAAATCGACTCATCATGTACCCGTAGGAATTGACGTCGGTTCAGAAGATTGGTCAGG

AGAAACAATAGCATGAGAGAGATGGTAGTCATGATGCCACGCATGTGCATATTTGGTGCA

AGTAGAGATACGAAGAGGAGCAGTGGGGTTGGGTGGAGAGGGCAACAAATCAGTGGCAGG

GATAAGCCTGGGGGTGTCGAGTCGGGGAGAGGAACCTAGTTCAGGTGGGGTGGGAGCCGA

GTCAGAGGTAGTGGAAGATGGGGGGTCTACATGGGCAAAGTCAAAAGAAGTAGGATATAG

TGGGTCCACAAGTATAGAAGGTGCCGGTTGGTTACGGGCTTGGTGGTTATGTGGCATTGG

TGTGGGCTCGGTAGGCCCAGAGGAAGAAGGAACGTCAGTTTGGGGTAGTGGCAGGACAAA

GGAAGGAGTAGGAGAATGAAATTGATAAGGAAAAATATTTTCAAAGAAGTGGACGTCCCT

ACTAACAAGGAATTTTTTGTGATCCATGTCATAAAGTTTGTAATCTTTTAGACCTGTTGG

ATACCCGATAAAGACACAACAATGAGTATGAGAATCAAATTTATGAGATGGATGGACAAC

AGTAGCATAGCTAAGACAACCAAAAACACGAAGGTGAGAGAGGGTAGGTAAACGATTATA

GAGAATCAAAAGGACTTTTATTAGATAATAAGGGAGTGGGTAAACGTTTTATAATGTAGA

CAGTGGTAAGAACACATTCACTGACAGGACCCGATCCTAATTCCACTTTGGAATTTGAAC

CAAACTCTGTGCGTGTCCGACATTTGGCAATTGTCGGGCACATATGTCCATTTTACCCTT

CCTGCTACAGTTACTATGAAAACTTTCTCTGGACTCCTACCGAAAATTCGGCAGAGTCTC

CCCTGTATTTTGACCAATCCCAAACTTTTTTTCCACCTATCAACAATCAAATAATTCTAC

ACCAACTGCCAGAATTGAATAACCAAGTATTCACCAGCTCCAGTTTTCCACTAAAACTAG

ATATCAGAGCATTTTCTAAAGTTTACAAGGTTTCAAGGGTTTTTCTACAAAACTCTACCT

CTTTTGGTGGCGCGGAAGCTATGAATAGCCCGGTGGTGGAATCTCTCGCACTTCTATGGC

CTGGGGGCGAAAAACAGGTTGAAAATGTGAGTGGACAAAATAATGTTCTTGAAAACAATT

TATAACCATAATAACCCCCATTTAAAAATAAATAAGGATGTAAATCGCTGGTTTGTCTCT

ATTTCAATACAAGGTATGCAAATAAGCATGTAAAAACATACTGATGAATATACTCGTATA

ACTGAACAACATATAAAGATATAAATCCGCAAATATCAAAGAAACTGGTATAAAATAATT

GGAAGTCACAACTGGATAAAAGAAAGCTCGAAATCCTTTGAAACTACCACATATTTGTAC

CCCTGTCATATCCATCAATTCCCCTAGCATGTCTCGGGCGTCACGCAGTCTACCCGAGCC

GCAAACTGGCAAAATCAGGGGACTATGATCAGCCTGATCCGCCGGCAGGTCTTGATGACA

CCAAGTCGACTCGAGCCGCTCTGGCAAGATACAGGGGACCGTAGTCAGCCTGATCCGCAA

TCCTGGCAGGTCTCGGGGACACAGAATCAGCCGAGCCGCAATCCTGGCAGGTCTCGGGAC

ACCAAGTCTGCCGAGCCGCAAATCCTGGCACTCACGGTCCGAGCGTCCCCAAAACTCGTG

AGGCAAGTCAAGTGCACTGACTAACTGAAATCAGACTGAATGTCCGTAGACATCGGTCCG

ACTGTGGGTAATCACCAAAGATAAATGGGTACACGGTGGTTTTAAAATAAACTATTTTGA

GAAAAATCTACTAACTCACTGCATTTCAGTAAAATCTGAAACTTATCTGCTATCTCTTCT

GGTAAAGTTCTCAAACTCTGTTTTTATATTACTGGAGCCTCAGTATCTAAGCATTACGTA

GATCAAAGTACTTAACAGTATAAATCATGCTCGAAGAAGCAGTTCATAAAACTTATTCAG

ATAAACTCATTTATAAAAGCTGATTTATATAAAATCAATATAAAATCATTTAAATAAACT

TATTTATATAAATCATTTATATAACTCATTTATATAAAATCATTCATGAAAGAAAGTCCA

CTCACTGATAGTCCGAGCTAGTTGGACCTCTTGAAGGTCCCTCCTGTGATCCTGTCGGGC

CTCTGGCGCCTAATTACCCATAAAGATTAAATTAATAAACTGCTGAATAAAAATAATTTA

AATTACACCCTGCCCCCGGCTCCTAGAAATACGTGCACTCGTTTTAAGTTACTCCTATGC

CTACCTTAGCTTTTTAGATAGTTAGAACGGTCTTAAGAGACTTGAAGCCTTACTAAGCGA

TAAATTACCAGACCGACTGATCCGGGACCCGTGGGGTCCATAATCTCCGATGGCCAATCT

GGGCTTCTCTGAAGGTTCCTCATAGAGAAGGAGCCATGTTCCGCGAATTTGGTCCGAAAC

GGACGGTCGGATTGGCCAAAATCGCATTATCGCTTTAAATTCAAACCCTAGCCCCAGGGT

TCGCGATTTCGGAGTATCCGGGACTCCGATTCGCAATCCGTCGAATCCTACATGATCCTG

GAATCATGTAGTACGACATATCCAAAATTCAGCGGGATCCGACAATTAGACGACACTGCA

CCCAAGGATCGCGCGGTATGGAAAATCCCTTCGGGGCTCAAACGGACTCCGAATCGAGAT

TCGCGAAATCCTACGCACTCGTGACGACACGAGGATCACAAAATTGCACAGAATTATTTC

ACCACCCTCACCCACGCTCCCCAGCACGCGCGGGCAGATTTCGGTGTCTAAAGCGAAACC

GGGTGCCGAAAAATCTCGGAACCAAGACTCCAAACTCCTATCCTAGGTAAAACACCCCAT

TTGGAATCACTTTTGTTCTTGGACATACCCCCAAAAGTGGCCGGAAATAGCCGATCACGG

CGGCTGAAGTTCGGCCGAATTTTCAAGTTGAAAATCGAGTGATCTAGAGGTGAAATCGAT

CGAAACCCGTACATCCATCAGTTAGAACACGAAAAATAGGTGAAAATCCATACCTTACTC

GAATGATTTGGTGGACAAACGAAGGAGATCGAGAGGAGAGAAGTTCGAATGAACACCGGT

CGGAAATCTCCAGTTTCCGACCAGTTCCAGGCGTCCCCCGGTCGGCGATGGGTCGGGGAA

GATCGGCGGGGTCGAGGCGGAGTTGACGGTACCCTTGCCGGAGATCGAGCCGGCCTGTGG

TGGCGACGGGAGCGGCTGGAAGGGAGAGGGACGGCTGCTGCGGGGGGGAGAGAGAGAAAG

GGATAAAAATATGACTTTTTGTCCAAATTACCGTTTTGCCCTTCGCGGTATTTTGACCGT

ATTTTCTTCGCTACAGCTCCGATTCGGGTCTACTCTGTGTCTACGGACTCGTTTCGCCGT

GCTCTACGCAATGGAGCAAACGGAGTTGCCAAATTCTTTCTCGATCAAAAAGTCAACTTT

TTTCCTATTAAATAATGCAAGGGCAAAATTGTCTTTTCGCTAGAAGATATTAATCCTTAC

TTTTTAGATATTTTGTTTTGGGTTATTACATTCACTCCAAAAATGTAATGGGATATTGGA

TTGAAAACGAAGAGCACGGGCGATATTTAATATGTGGTGGTGCTTGCGTTCTACCACACC

ATTTTCTTGTGAAGTATAAACGCAAGAGAGTTGAAACTCTGCCATGTGAGAGAAATAAAG

GTTGCATGGAAAGAAATTCAGAACCATTGTCAGAATGGGTTGTTTTAATGGAGAGATTAA

ATTGAGTTTGAACAAAAGCAATGAAATTTTTGAGAAGATATTGAGTGTCAGTGCATCTAT

TAAAATCATCCACAATAGTTAGAAAAAAAGGAGCACCGCAATGAGATTTAATGCAGTGCG

GCCCCCAAATATCACAATGTAATAATTGAAAAGGAAACTTGGAAGTAATATTACTTGAAG

GGAAAAGAATCCGAGTCTGTTTGGCCACAGGATAGACATCACTAGTATTGTCAAAAGGAA

CAGAAATAAAAGGAAACAATTTATGGAGGAGTTGGAGACGAGCGGGTGATGGGTGGCCAA

GCCGTTGATGCCAAAGATGAGAAGACGGTGGGATGTGACAAGAAATTGGTGATGGTGAAG

GCGGTGACATGTAGTATAGACCATCACTTTGTTTACCCCAATCAATCATCTTCCCCCGTA

GCCAAGTCCTGCAAGACACAAAATGTGAAGACCCATATTTTTAAAAAATATGAAGAATAA

ATATTTAACTACCTATTTAGTTATATATTTAAATTGATTATGCATATAGTTGTTTTACCA

TTTTAATAATTATATGTATTTTATTATATTAAATATGTATGTAATAAGCATGAGGTGGAT

AACATAAGCTGGAGGTGGCACCATTCCTTGGTTGCTTAAGGGACGTGTAAACACATAAGG

TTGTTGTCTTGCTATCCAAGTGCTAGCTGGCTTTACTTCATCAAATGTTAGCACTTATCT

CCAACCTCCAAACATACCAACCAATGATAGAAGGATAGTTGCCTTGCCGACTTTATTCTA

GTATAAATAGGAAGCTTACAAGTTGTTTACTACTACAAACATGCAACGTGAGAGAGAAAG

AGAGAGGTGATGAAATTTTTGGAAGAGATATAGGGTTTAGCTTTTATTTTCTTAAATTGG

AGCAATGCTTTGGAATCGAGGTAGGGATTTGTTGGTTACATTTACAAAGATTGATCTAAT

TAAATTTCATGAGCAATATATTATGCATGATATTATAATTTGATTAATATTGACATGTTC

ATATGTTATTGGTGATGGCTGGAATTCGTAGGTTGATAAAGAAATTATGGAAAAGAGGGT

AATGGGAAGGTTCTTTTGTGTAGTTGAGTCTAACCATTGTAGGGACCAAGTCCAGGCAAG

CCCGTGTGCATAGTAGATTAGGAAATCGGAGGTATAATTGTCAATTGGGTGATTTGGGCT

TGGAGATCAATGGTGTAAACAAGATGACTAGCAAAAAAGGAATTATGAGATAACTGATAT

GGGTGTTAGTTTATGTAATGGGGCATTCGGAATCACAGTAGTAAAATACATGGTATGGGA

CATGCAGGTTTGCTTGCCCTATTATATAAATTAACGGGTTGGGTAGAATTGAAGAGCATT

CATGCATTATCATTATTATTATTAATATTTGGCTGTGTGATTGCATGTCACTTAATTTAG

TTATATCAATCTATTGTGGTAAGTCTCATGTCCCTACTGAGCGGTGGTTGCTCATCCCTC

ACGTGTTTAACTTTTCAGATGAATTAGTGGGCAAGACCAGGACTAAATCAATTCTGGCTG

AATTAGATGAGAGTAGTACAGCTTTATTTATTTCTTGTACAGTTGTAAGATTTTGGAGCT

ATTTTTATAAGAGAAGTTTATTTTGAACCCATTTTTCGGCATTTGTTTATTTTATTGTAG

TTTTTATTTTCTCATGCACCGGGTGCGGGGTTTTCACATACCCCTAGTTCGGGGCGTGAC

ACAAAAATCAGGAAATAAGATGATGCAACAACGCAATGATTTAGTGATTTTACTGGCAGA

TAGAAGATTCAAATTGAAAGATGGAACATGAAGAACATCTGTGAGAGCAATGTCCTTATT

AAAAAGAATTGTGCCTATGGAATCAATGGGTATATATGAGCCTATAGGTAAATTAACGGA

GGGTACATGTGAGGTATTCTTGTCAACGAAACAAAAGGAGTCAGATGTAATGTGATCCGT

AGCTCCACTATCAAAAATCCAAGGATGAGAAAAAATGGAATTAATGGAAGGGAGAGAAGG

ATTAAACAGACCTACCTCATTAGCAAAAGCAGTGGAGTTGTCAGATAATGAAGGGGTGGA

ACATTTTACCATAGCAGCTGTAAGTTGTTTGCACCGTTCATCAGTGAGCCCATAGAGAAC

AGATTCCAAATTGGAGGCCGACTGGTTCGGTGGCATGGTGCGACCCATTGCCATTGGACC

GCTTGGACTAGTGAAAGGGAGCTGACTGGCCTATTGCACTAAAGAGGAATTGGCGTCCAC

TTGATGGGCTGTTGGTGTTTGTCGCGTACTAGAATTACCACCATAGTTGCGCCCAGAGAT

ACGGTTGCTGCCAGAGTTACTGCCAGTTGAAGAAGCAGCGTTGTGCCTGGGTGGGTACCC

GTGCAACTTGTGACAGGTTTCAACAGTATGGTAGTCACGATCACAGTAACTACAATGGAG

AGGATTGCCGTTGGCGTCATGTCCGCGAGATCGATTTTGAGAAAAATTATTACTTTTGGT

ATTTCGGACGGCTATTCCTCTTTGTTTCTCTTCTTGAGTAATCAAAGAATATGCTTTGCA

GACACTTGGGAGAGGATTCATAAGTAGAATTTGACCACGGACTGTGTTATAAGAATCATT

TGAGACCCATTAAAAACTGCATGATGTGATCTCGATCTTTCTATTCATTCTATGTTTTCA

TACCACCGCAAGAACAAGTAGGAGAAAGATTATAAGAAACAAGCTCGTCCCATAAAGCCT

TGAGTTTGGTGTAATATAAGGATACCGACATGGTTCCTTGAGTGTGAGAAACAATCATCT

TTTGGATCTGAAAAATTCTTGGAGCCTTGCCTTGGGAAAATCTGTCTTCAAAATCTTGCC

AAATTTCTCTTGCGGTTGCTGCGTAAACCACGCTATCTGCCAAGTCAGAGTCAACTGAAT

TTAATAGCCAGGAAAGAACCATCTTGTCACAATGGTCCCAAAGAGTGTAATCATCGGACT

TCTTGGTAGATCATGGTCGTTCGATGGAGCCGTCAACGAAGCCAATCTTGTTCTTGGCAA

GTGAGGGCAATAACAATGGAACGTTGCCAGGTTGCATAATTGTTACCATTAAGCTTCTTG

GAAACTAACAGAAAGCCTGGGTGATCAGAAGAGTGAATAAAAAGAAGGCTGGAAGACTCC

ATAACCGAATTTTTGTCAGCGGAGGAACTTTCTTTCTCACCCATGAAAATAAATCAAGAG

TCACCAGGATGGTGCTCTGATACCATGTAAAGTGAAATATGGAGAATATTTTCTCTGTTG

ATTGAATAAATCATACAGGACAATACAAGGAATATAAAAGATACACGAGGAGCTATTCTA

AGTAACTAACTAATCTAGGCTAGAAATCTGGTATGACTCACATAACATGGTGTTGTGTGC

ACTACAAGTAAATACAAAATTACAGAAGTTAAGGGATTTCTTTCCTAACAGAAATGAAGA

TGAACATCTTTTGAGGCCATTCTATCAAACATTCTTTGATGTCCTCTCTCTCTCTCTCTC

TCTCTCTCTCTCTCTCTCTCTCATGAACATAATTTTTTATTATCTTTCTTTTGTTTCTAT

TTTATAGGGTGCCATTCAGCGCGGTGAAAGATTTGAATATGGTTATCGATCAAATAAAAT

CCCGGCATGGTTGAGCCGTCGGTGTACTGAATCCACCATAACAATCCCTCTACCTCTTGA

TTTAGATGGTAAGAGTAAATGGATAAAACTTGCTCCGTGCTTTGTTTATGAAGCAGCTCA

GAAGCATGATAGTTTGGAAGATGTACCAGAATTTGATGAGGAACTGGGAGCAAACTTCAC

TCGCAATCACCGTATTGAACTCTATACAACTGAAGATCCTCATGAACGTCCCCAAGTAAT

TATTTACCACGACTGCAATTTAGCGGGACCATTTATACATAGGTGCTTTATACCACGAAT

TGACCTTAAGGAAACTTCAAATAAACGCTTGATCCGAGCTTCAATTACACCCAACAGCCC

AGGGACAAAAGTGACAGGGTGTGTGGGGTGAGCCTAATATAATTGGAAGATGTCCCCAAA

TTTGTCCAGAAATTGAATAAACACTATCTTGGCTATCAGCATCAACAAGAAGAAGATGGT

ATGAGAAGTATTTCGTCGACAAGCGGGGTTCAAACTCAACAAGCAGTACAAGAATAAGAA

ACTACTACTTCAACCAGGATTGTAGGTCAATTGAGAAGAAACGTGGTGTCATTAGGCAAT

GATTGTTATCATGCACACAGACACTGTGTGATCTCATTCTACTTTCAATATATATTGTAG

CTAATACAAAAAATGACTCAGCCAATACATTTGTACCAGCCTGTGAGCTGTACACTTGGC

ATGAATCTGGACATGTTAGTCCAACAATATGTGATGGCATATGAGTCATACACCTAGCTA

ACTAGCAATCTAAAAACCTGAAACAATTTATACTTCAACACTCCCTTCTAAATTGTTAAC

TATTTTAACACCAAGCAATCCTCTAAAATAGTTGAACCGATCTTTAGGCAGAGCTTTAGT

GAAAATGTCAGCCACTTGTTCTTTAGACTTGCAGTACCTCAAGTCCACCACTCCATCTTG

CAAAGCATCTCGGATGAAATGATACTTGCGGTTGATGTGCTTGGTTTTCTGATGAAAAAC

AGGATTTCTTGTCATTGAAATGGCAGAGGTATTATCCAGCATTAATGGAGTTGCCTCAGT

CTGCAATTCTCCAAAGTCTTCAAGCACAAATCTCAGCCATATAGCCTATGGTAGTAGCCT

CACTTGCGTTGACATATTCAGCTTCAGCTGTAGAGAGAGCTACACTATGTTGTTTGACTG

ATGCCCAAGAGAATGCTCCACTACCAAAGGAGAAGGCATAGCCAGAGGTGCTTCTCATGT

CATCCTGGCTTCCACTCCAATCACTGTCACAATATCCAATTAGGATAGCCTCTTTTCCTT

TCTGATATTCAATACCAAAGTCAATAGTGCCTTGAATATACCTTAGCACCCTCTTAGCCG

TTCCATAGTGTTTCTTTGTTGGACAATGCATGAATCTAGCTAATAGGCTAGCTGCAAACA

TTATGTCTGGTCTTGTAGCTGTCAAATACAGTAACCTACCAACCATCTGTCTATATTCAT

TTTCATCTGCAGGTTCATTTCCATCATCCTTACACAGTTTCTCACTTGCCACAAGAGGAG

TACTAACTGGTTTACATTGTTTCAAACCTCAATAATGTCAGTGCATATTTCTTTTGGTTT

ATGAAAATGCAATTTTTAGTTTGAATCACTGCCATACCAAGAAAGTAATATAGCAGACCC

AAGTCAGTCATTTCATACATTCTCATCATTTCAGTCTTGAAATCTTCAATTAATTCTTCA

CTGCTACCTGTATACACGATGTCATCAACATAGATTGAGACTATTAGAGTTCCAGAGTCT

TCTTTAGTCTTGCAGTACAATGTAGCCTCGCTTGTGCTTCTGACATAACCACAGCTAATG

AGATAGGCATTGATTTCCTTATACCAAGCTCTTGGAGCCTGTTTTAAACCATACAAGGCT

TTCTTCAATTTGTACACCTTGTCTTCATAGTTTGTAGTCACAAATCCATCCGGTTGATCT

ACATAAACTTCCTCCTTAAGCACACCATTTAGGAATGCAGACTTGACATCTAATTGGTAT

AGTTTCCATCCCTTTTGAGCTGCCAAAGCTATGAGAGTTCTAATTGTGTCAAGTCTGGCC

ACAGGAGCAAAAGTCTCATTGAAGTCAATTGCAGGCTTATGTGCATAGCCTTTGACCACC

AGCCTAGCTTTATGCTTCTGGATTGAGCCATTCGTCGCGCAAAACTAATATATAATTAAT

ATATAATTATAATATAGCTTTTGTTTTATTTTATAAAAAAATAAATTTGGTTTTTTTTTT

TGGGGTAACAAAATGAAATTCCAATAAAAAAAAATTATTTTATAAAATAAATGTGTGCTA

CAAGAGAAAGATTTAAAAAATAATTACGAAAATAAAAAAATTAATCGAATAAAGTTCCAA

AATCTACATTATCGTCTGGCACATGCGGTTCAGAGGTCTGGGGGTCTACAGGGGCCGGCA

TCTGGTGTGGATGCTCGGGATGGAAGGGCTCGGAGGTCGGCGCATCAAAATGCGGGGCTG

GAATGCCGGACTGTGCGAGGGACTGCAGAATGACCGACATCTGGCTCTGAAGAGTGGCCA

CCTGTGCTGTCAAAGCAGTGACCTGACTGTTTGACTGCGCTGATGAACGGGGTCTGGGTT

CCCTCCGCCTGGCATTCCCCATCCCTCGACAATATGTCCCCGGTCTCCTCCCTAGAGTCT

GATCTAATGTCTCCGTCAAGATCTGAAACCCAGCATCCTGTGGAGGATCCACAGACTCGA

TCGGAGTATCGGGAGGAAATTGGGAGGCGGACTCCTGAAGAACCAACTGGCTCCTCTCCA

CCATCGTCGTCTGTTTAGCATAACATAAAATATAAATTCAAACACTCATATAATGAACTT

TAAAGTTGAATCGTAACATAAGAACTAAAATTAAATAATACTTACATGAAGGGACTCGGC

CAACTCATTCCCAGGCCGAACATAAACGTCGCCAAAGACGTCGATCTCTGGGAATTTGGA

CCCCCCCTAAATTGAACAAAAGAAGACAAATATTAGAACGACAAAATAAATTAAGAATAA

TGAAAAAGTAAAAAATTAAATAAAAATTATTCTATTATTACCTGACGCCGTGCATCCATC

CGATAGGAGAAGGGCCTGGAACCCGAATGATGGAGAAGAGTCTTCTTCTTCCTATTGCCC

TTATTGACTTTGGCTTTATTCTGTTACACAAAAAATAAAACGTATTAGTTGAAATTGAAA

TTAAATATAAAAAATTAAAAAAAACATTTATAAGAAAATAAAATACAATATAAAAAATTA

CCACAAAAGCGGGCGCCTGAAAATGAGCGCAGAGCCACGCCCAACTATCCTCCCGCCCCT

TAAGCTCTTTCGGGCAACCCTCTTGAAGAGCAACTTGCGGATCATCGAACGCCTCAAAAT

GGTAGTGCAAGTCGCTCTTCCACTGCTTGTACCTCTCAGAGAAGATTCTGTTGACGTACG

CCAACGACTCTTCGTCAAGGTCCTCCAAGTTGTAGTTCGTCTGCAATAAATTAATTACAT

ACATTTAAATAATATAAAAACTACGTTTGAAAGAAAAAGAATGAAAAGGCCTACATACTG

ACAACTGGCCGCGAACCGCAGTCTTGGTCTCGTCAGGCATCACCTTCCAAGACTTCCATT

GCATCGGGCAATAGCTCCGAATGACATGACCAATGTCGTGGGCCAAGGAGCTATGCAACT

CCGCCGTCGGTGCAGCTCGATGTTGCTCGTCGTATCCGATGTTGATACGACTATTGGTCA

CCCGGGTGACCTTTGCCGTCTTCAATTGCCGACACGGCCCTCGGGTGTTCTTCTTTGCTG

CAAAGAAAGATGACATGAATGTTAAATAAAAAACAAATTTAACAATCAAATACACACACA

CACAAAAATTAAAAAACAACAAGAAGAAATAAAAAATAAAATTAACTGAATCAAATGGAA

AGCTATACCTGGCTGTGACCCCGACGCATCCTTGGATGTGGTGTCGGTGGTGCTGGCGGG

CCGATGACGCCGCCTGGTGCTAACAGGCTGCGCCACTGATGACGCTGATGAGGCAGGCGC

CTGGGACCCCGCAGGTCCAACTGCCAAGTGGTCCATCAGTGCTGGTGCAGTGGCAGCAGA

TGTCGGCTAAGTCGGGGGCTCCGAACTCTGCGTGGTCGTCACCGCCCTACGACGTCTAAT

CAGGTCTGACATCTGCACAATTTCATAAATACAAAAACGAATTAATAAAATATACGAATA

TCCGATGTTGATATGATACAATTCAGATTATTGGCCTAGGGTTTACGAGTTCGGAGTATC

CGGGACTCCGATCCACGATCCGTCGAATCCTAGACGATCCAAGAAATACAACCTACACCA

CACGTGAGTTTGAGTTCGATCCAACGGTCCGAACATAACAACCCCGGAAATCGCACAATA

GGCAAAATCCGTTCGAGATGCAAACGGCAACCAAATTCCAATCCGAAAAAACCTATGCGC

TCGTGACAACTAAAGGATTGCACTGGGACCTAATTCAGGCTGCTACAGTGCCCCACGCGC

CGCCCCAAAGCGATACCAATCGTCGAAAAATTCGTAAAACTAAGGGCCAAGGTTCCTATC

CTTGGTTGAAAACATCATTTGGGGCCACTTTTGTTCTCGGACATAGCCCGAAACCTGGCC

GGAAGTGGCCGGAATTGAAATTCAAAATCCGGCCGAAACTTAGGGTTTCAAAATAAGCGG

CCTAGCTCAACAATCCAACAAATCCTACCCAACCATCAGCTAAAGCATGAAAAATAGGAC

AGAAACCATACCTCAGGTGTCGGAATTGGTTGTCGGATGAGGGAGAATCACGGCGGAGCC

GATTCGGTGAAAACCGAAGAAATCGCGGCGGCTGGCTTGACGGTGACGGCGGGGGAGCTC

CGGGGTACCAGCAATGTGGCGGGCGTGGTCCAAATCCACCGAGGTAGGAGGCAGCGGCGG

CGGCGGGGAGAGAGATGGCGGAAGGAAAAATTTCAGAAATCTGGGGAAGAAGGAGGCCTT

CGCGTGGTTTCTGAAATTTTATTCGTTTTTGCGCGATGATTGGAAACTGTGATCACCACT

GTAGTATCTCTCCTAAACTCTTCCTGATTAGTATTACCTTCATCCCAGTACTGTGCGCAG

AGCGTACTCCTTGGTCTGCCTATTATCTGACTGGGACAGACATCACCACAGCATCCACAT

TGACCAACTATTAATCGGATCGTAACTAGGTACTACCTCTTACTTTCCAATCTTTATCTT

CTATTCTACTGTAAAAACTGCTGCATTCACGCCTTCTGTATACTTGTAGATAGGACTGAC

TATCATATCTGGCTCTGAAATACTTGTCCAATACCAATCAAAGAATTCTAGTATCGTTCT

TAACCAAATCCCATCAATTCTCTATTCCTTTGTGAAACAACCAGAACCTCCCAAATCTAC

TTTTCTCCTTCAATTTAATCCTCTTTTTACTCCTCGGTACCACCATTAACCCAATTTATA

ACTCTCACTCCTTTTTACTCGGTCCACCTATCTCACGGGTGGACTTCTTGGCTATTCAAT

CTCTACTAAAGTTAAAATTAATGACTTCAAGGTCTAGTATACGTTAGGGTCTCCTTGTTT

ACTAAGCACAGTAGGTACAACTCCTAACAAGCCTCTAACAAAGGATTTCTCTGTCAGTTC

AGATACCAGTGCAATTCCTGTCTGACTTGGAAATTTCATCCCCTTACGTCGGGATAACTA

TCTAATTTTCCTACGGATACCACCACCATTTGGGCCCAACTTGAGGAGAGAACGAGGATG

CACAGAGTGCGAAGTCTACAGGAAGTAAAACCTACGCTCTGATACCAAATTGACAGGACC

CGACCCAATTTCCACTTTGGAATTCGAGCCAAGTCCTGTGCCCTTCTTACTTCAATTTCT

CTTTAAAGTTCCCTTAGACTTCTGCCGAAAATTCGGCAGAGTCTCCCCTGTATTTTGACC

AATCCCAAAATTTTCACCTGTTAAATATTCAACAAAACCACACCAACAGCCAGAATAGGA

TAAACCAATATTTGCCAGTTTCAGTTTTTCCACTAAAGTTCGATATCAGAGCAATTTTCC

AAAGTTCACGGGCTTTCAAGGATTTTCTACAAACCCTACCTGACTTTGAAGCGCGGAGGC

TAAGAGTGGCCCGGTGGTGGATCCTGTGCACTTCTACGACCTGGGGGCGAAAAACAAGTT

TTTTAAACTGTGAGTGGACAAAAAGTGAAATTCTTAAAAACAATCCAGAATATAATACCC

CCGTTTTGAAATAACTAGGGATATATATATTTATATATCGAAAATTTAACTATATCTCAA

TATAAAAGATTCTATAAGCATGAATCAGTATGCTGATGAATAAACTTACATAATCGATCA

AATATGCAAAGATATCACTGCCTGGAGGAATCAAGAACTGGTATAACAGAATAAAGATAT

AGAGATATAACTGTGCGAATACTAAAAAAAACCGATATAAAATCGCTGAAAGTCATCCTT

GAATAAAAGAAAACTCAAAATCCTTTGAAAATACCACCTATTTGTACCCCTGTCATATCC

GTCAATTCCCTGGCAGGTCTCGGGCGCCACACAGTCCACCCGAGCCGCAAACTGGCGAAA

TCAGGGGACTATGATCAGCCTGTCCCGCCGGCAGATTCCTCGATGACACCAAGTCAGCTC

GAGTCACTCTGGCAGGATGCAGGGGACCGTAGTCAGCCTGATCCGCAATCCTGGCAGGTC

TCGGGGACACCAAGTCTGCCGAGCCGCAAATCCTGGCACTCACGGTCCGAGCGTCCCCGA

AACTCGTGAGGCAAAGTCAAGTGCACTGACATAAACTGAAATCGGACTGGATGTCCGTAG

ACATCGGTCCAACTCTGGGTAATCACCATAAAGAAACGGGTACATGGTGGTCTAATTAAA

AATCTGGTAACTTTCTGAAATCTGATAAATTGACTGTAGTCTCGATGCTGTTGCTATTCT

GTTATATAAAACTCGAGGTATGCTACTAAACATAACTTTCATCATATATGTAACTCGAAA

TATTTAACAGTATAAAACATGCTCAAAGATTTGCAAAATACTTATTCAAGATCAAATAAT

AAGATTCGCTTATAATAACTTATTTATAAAAACTTATTTATATAAAATCGAGATAAAATC

CTTGTATAAAGTCACTTATAAAATCTTATTTATGGAAACCTCTATATAACTTATTTATAT

AAGATAATCCATGAAAGAAAGTCCACTCACAGATGGTCCGAGCTAATTTGGCCCTTTTGA

AAGTTCCTCCTGCTGATCGGCTGGACCTCTGATGCCTGATTATCCATGAACAATAAATCA

ATAAACCGCTGAAAAGAAAAATGAATTAAATTAAACACCCTGCCCCCGGCTCCTAGAAAT

ACTTGCGTTCAAATCCAAGTTACTCTTGAGCCCACACTAGCCTTTTCAGACACTTGGACC

AGTTTCAAAAGTTCCGACGCTCAGCTAAGAGATACACTGCCCAATCGGCCGACCCGGGAC

CCCGTGGGTCCACGACCTCCGATGGCCAATCTGAGTTTCCCTGAAGGTTTCTTACAGAGA

GGGAACCATGTTCTGCGAGTTTGGTCCAAAACGGACGGTCGGATTGGCCAAAATCGCGTT

ATCGCTTAAAATCCAAACCCTAGCCCCAGGTTTCGCGATTCCGGAGTATCCGGGACTCCG

ATTCGCAATCCGTCGAATCCTACACGATTCTGACATCGTGTAGACCGACATATCCAAAAT

TCAGCGCAATCCGATTATTTAACGACACTGCACCCACGGATCGCGCGATATGGAAAATCC

GTTCTGGGCTCAAACGGACTCCGAATCGAGATCCGCGAAAACCCACACGCTCGTGACGAC

ACGAGGTCACAAAAATTTACAGAATTACATCACTACCCTCGCCCACGCCCTCCAGCACGC

TCCGGCAGCTTTGGGTGTCCAAAGCGATTTCGGGTGGCCGAAAAATCTCGGAACCAAGAC

TCCAAACTTCTACCCTAGGTGAAACACCCCATTTGGAGTCACTTTTGTTCTTGGACAACC

ACCAAAAGTGACCGAAAATGGTAGTTTCGAGCGGCCGAAGTTTCGGTCAGATTTCAATTC

GAAAATCGAACCCCTTAGAGCTAAAATCGCTCGAAGCCCATATACCCATCAGCTAGAGCA

CGAAAAATAGCTGAGAAACCATACCTTACTCGATCGATTTGGTGGAGAAACGAAGGAGAA

TTTCGAGCTGGAAGTTTGGGTAGCTTCGGACGAACCTCCGTCGGAAAACATTATTTTCCG

GCCAGCTCAGGGCGGCCCCGGGGTGGAGGTTGGTCAGGTTGTGACGGCGACACGGAGGCG

GGCCCTTAGGTACCCACGGCGGAGATCAGCTCGGCCTGTGGTGGCCGGGCCGTCGCCTGG

AAGTCGAACGGGCGCACTGCCCAGTTCGCGCGGAAGGAGAGAGGGGAAGAGAGAGAAAGT

GACGGGGGAGAGAGAGAGAGGGGTATAAAAATCTGACTTTTGGCCGAATTACCACTTTGC

CCTTCGCGGTTTTTAGACCATAACTTCTTCGTTACGGCTCCGATTCGGGTCTACTCCGTG

TCAACGAACTCATTTCGCCGCGCTCTACGCAATGGCGTAAGCGGAATCCCCAAATTCTTT

CTCGATTAAAAATTCAAATTTTCCCCCATTAAAATATGCGAGGGCAAATTGGTCTTTTTG

CTAAAAGATACTTTCCTTACTTTTTAGATATTTTCTTTCTTTTTAGATATTTTGTTTTGG

TTCTTACAGCCAGTCCGCCCAAATTTGGGAATTAGGGTTTAGCGGGCAATATTGAAATCT

TGCGTGACGCATTGACAAACGTCACGTCGCGCAAGAGATTTTCAATGGGTATTGCGCGAT

GAAAAATACTTTTATTCCGTCGTCGCGCAATGTTGGCAGTCCCCAATTTTGGGATTTAGG

GTTTAGCGGGCGATATTGAAATCTTGCGCTACGGATCTAAAAGCATTTCGTCGCGCAATG

TTGGCAGTCCGCCAAAATTTGGGATTTTGGGTTTAGCAGGCGATATCCAAATCTTGCTCG

ACGCATGGATGAGCTGTACGTCGCGCAATGTTGGCATGGCCAAATTTGGGATTTAGGGTT

TCGCGGGCGATATTGAAATCTTGCGTGACGCATGGATTAGCAGTATGTCGCGCATTGTTG

GCATTGGCCAAATTTGGGATTTAGGGTTTCGCGGGCGATACTGAAATCTTGCGCGACATA

TAGTTAAGGTTTTCGTCGCGCAATGTCTCGCAAAAAACCCTCAATAATATGTGGAAAAAA

CCATTCGAAGAGAAAGGCCTGGCTTCTCTCTTGAGCGACGACGAACACTTCTCGTTGCGC

AAAACCACCCAAAAAAATTTTCACCAAGTGTCCAGGGAGGAAGAGTGGGGGTGAAAGTTC

TGGCTGATATTGCGCGACGAGGTCACTGTTGTCGTCGCGCAATGTCCTTAAAAACTCGAA

ATCAAATGTATGGAAAAACCGTCCGAAGAGAATTAAGGCTGGCTGCGATTGCACGACGAA

CAGGTACAGCGTCGAGCAAACTTATGCACCCAAAAAATTTTCACTAAGTCTCCAGACGGA

GGAAGAGTGGGGGTGAAAGTTCTGGGTGATATTGTGCGACGAAGTCATGTTGTTCGTCGC

GCAATGCCCTTACAAACTCGTAACCCAAAATATATGGAAAAACCCTCCGAAGAGAAAGGC

TGGCTGCGATTGCGCGACGAACAGTACAGCGTCGCGCAAAACACCCAAAAAATTTTCACT

AAGTGTCCAGAGTGAGGACGAGTGGAAGGTGAAAGTTCTGGTGATATGGCGCGACGAAGC

TAACGTGTTTTCGCCGCGCAAGCCCGTCCTAGTTTTAGTCGCGCAAGCCCGTCCTAGGCG

GGCTTTGTGCGACTAAACGCGTGTTTTCGTCGCGCAAACCCTGTACCCTAAACCCTAAAG

CCTAAAGCCTAAACCCTAAACCCGAAACTAGTTTTCATGATTCAACCGGTAAACTTGTTT

TTTTATACTTCGAGATCGTATATGCCGAAAATCGAAACAAACAAACATTAAGAGATCAAG

TAACAGGACGAAACTTTTCGACGGTTGTAAATGAAAAGTCATGATTTAACGGTTATTTTA

GCTCTGATTTGTAAGATTTTTTACTGCTACTTTTCTGTACCCAATACGAATACAATTAGT

GAATTTGATCATCAATTTCAGATGTTTACACTAGGGTGATAAGACAAAATGTTATGTTAT

GTTTAACGAAAGTATAAGTAAACGATTCATTGTTGGTGAATATATGATTGCTATGGCAAA

CGCATTCTACGAAAGTAGTTTCAGCAATCCAACCATCAAACTTGTTTATTTGTACTTCGA

GATCTTAGACCCCAAAAATCGAAAAAAAAAAACATTCGGAGATCAAGTAACGGGACAAAA

CTTTTGAACGGTTATAAATGAAAACTCATGATTTAACGGTTATTTTAACTCCGATTTTTA

CGAATTTTTACACCTACACTCCATGACCCTATATGAATACAATGAACTAACTCGATCGTC

AATTTAAAATATATATGTGTATATATAATATATATTGTAAAATCATATACATATTTGTGC

GACGAAGAAATATTTTACGTCGCGCAAACGTTCATCGCGTTGACGCATGAAACTTTGTCG

CGCAATAATTTAACTCCACGCGACTAGTGTGATATAGGTCGCGCAAACTTATGCACGACG

AGATTTCTTCGTCGCGCAAAATTTGCACGACGATGCGTACTTCGTCGCGCAAAATTTGCA

CGACGATGCGTACTTCGTCGCGCACGGGTTTTCAGTTTTGAAAAATGTTTATTGTCAATA

ATTTTCAAACTTTGTATAAAAATGTTTGGATATAATTATTTATCAATGTTTTATAACAAA

TTTTGTATCAGTTTCATTTTGTAAATGTGTTTTGTGAAAAAAAAAAATTACAAATTGTTA

TTAAAAGGGTTTGGATATATTGATTTACCAATGTTTTATAACTACATTTATTTGAATAAT

GTTTCAGTTGTCCACATATATAAGATTTGGATATTTGTACTACATATATTCGAACATTTG

TATTACACAAAGTCTGTACACAAACATAATAAAATTAGAAAAAATTTAATTATTCATCAT

CTGAACTATAATAACTTTCGTTATCGTTGCTATCATCACTTTCGGTCTCCCAATCCTCCT

CCTCCTCGTCTGTTATAACTACATCATCTTCGCTGCTCGGGCCCACTGCAACATCGAATC

TTGGGAGATCCCCGAGGTCAATTGTAATTGACTGAATCGGTATTTCGATTGAAGACACTC

CTTGAATTTGAAACGGTTCTTGTATAACATGTGTATCTCGAAGTGTATCCGCATCATTTT

CCATTGATGATTCTAAACGTTGGTCAGCCACATTGTCGACGTCGTTGTCAGTGTTCCCGT

CTAATTCTGGTATAGCATACACGTTCCTATGATCCATCTTCTGAACAACTTTCCAACTGC

TCCTGGCTTTAGGGTCATCTAGATACACAATTTGTGTTGCCATGTTCGCCAAAATGTAAG

GGTCGTCATCATACCAAGTTCTGTTAATGTTCACAGATAGTAAGCCATGGTCGATTTTAA

CACTTCCCGGCCTATTTGGGTTGCTATCAAACCATCGACACTTAAACATGATCACTTGGT

ATCGATCTTTGTAAAGCAATTGAACGACATTTGTGAGTTTGCCATAGAAATCAATGTTCG

TACTTTCGCCTCCACCTGGGACATGGACACCGCTGTTTTGCGTACACAACTTGTCATCTC

GTGCAGCCCCCAAAAATTTAACACCGTTAATATAACAACCCGAAAACAATTCTGCGCGAA

TAGGGCCGAAGGCCAAGTTATATAACTCATCACTGTAAGTGGGGGAATTCGATGATTTCA

ACTTATTCACCTAATATAATAGAAAACCATTCACATGTTAGTCTGATAAAATTAAATACT

AAAAAATTTATATTTATCAAATACAAAACACTTATAACTTACAGATTCCAACAACCATTG

TGGAAACAATTCACGTTGTTTCTGGGCAACTAAATGTGATGGATGTTCTCGCTTCATCAT

CTCTTCATGCTCATCTAAGTATGCCATTATCTCATCACAATTGTTCAGTACGAACCAATG

CGCTACTTCCATGTCATTTGTAGAAAACGACTCTCCTCGTCCAGGATCTCCAAAGGGCCG

TGCGCTTTGGGCAAAAACAGAAAGTTTTTCCTTTCTCATACCTCCGTCGTTATTACGCTG

AGGACGATTGAAAGCCATCTCCACATCTTTTAAATACATTCCACAGAAAGTAAGCGACTC

ATATTGAACCCAAGCCTCTATAATTGATCCTTCGGGCTTCGCCCTGTTGCGTACACTTTT

TTTCAGCTCTCCGAGAAGCCTGTCAAAATAAAAAGATATGATACAATTTAGCAAGCCTGT

GATAATGATGCATACACAAACGAAAAAGATTATATACCTTTCTATTGGATACACCCAGCG

ATAGTTGACAGGACCAGCAAGCAATGCCTCTTCTGGCAAGTGAACCATCACGTGCATCAT

ACTTGTGAAGAAAGCTGGAGGAAATATCATCTCAAACTTGCATAGGACTTGGACAATGTC

ATGGCGCAACTGCAATATGTCTGTCTTTCGCAATGTTTTTGCCGTCAGTTGGGAAAAAAA

TCTGGACAACAACATGATTGGCTTCACAACATCTTCCGACAATAGATGTCGAATACCCAC

AGGAAGTAGGCGTTGCATAAACACATGGCAGTCATGGCTCTTAAGTCCAGTAAATTTACC

CCCGTCAACATTCACGCAACGGGCGATATTAGAAGCATACCCATCAGGAAACTTTACAGA

CGATACAAACTTTAGAAACTCTTTTTTGTCATTCGGTTTCAGAGAAAAGAATGCAAGATC

CCTTCTAGCTTTATCACTATCCCTGTTCATCCATAATCCCCTTCGTATGCCCATTCTTTC

CAAATCAAGACGGGCTTTGATTGTGTCCTTTGTCTTGCCGTCGATATCTAGAATCGTACC

CACCAATGTGTCAAATACATTTTTCTCAACGTGCATCACATCTAGATTGTGCCTCAATTT

GAGTTTCGACCAATATGGGAGCTCAAAAAACATAGGCTTGTGAGTCCAGTTCATATGTGT

AGAAGGTCTGGTCCGGCTAACTGTTTTTCCGAACGGAGGAAAATCCAAACGGTTAAGCTG

TTCCACGATCTCATCACCGGACCATTCTCTTGGTCTGAGGCGACGCTCTGTGTTCCCGTC

AAACTCTTTATCTTTTTCCCGCCACTTGTGGTCCCATGGCAACCATCTCCGATGACCAAG

GTAACAAACTTTTCCTGCGTGCCAACCAGAAGTTACATCTTCCTTGCATACAGGACATGC

CATATAACCCTTTGTGCTCCACCCAGAAACCATTGCATTTGCTGGGAAATCATTCACAGT

CCACATAACTGCTGCCCGCAAAGTGAACATCTTCCCAGTTGATTTATCGTACGTGCGAAC

ACCGTTTGTCCATAAATCCTTCAGCTCATCAACCAGCGGCCTTAAGTAAACATCGATTGA

CCTCCCAGGATCCTCAGTTATCAAAACAGTCATCATCATGTATTCTTTTTTCATGCATTT

TCAAGGCGGCAAATTATATGGAAATACGAAAATCGGCCAAGTGCTGTGGTGTTGGTTTAG

AACCCCATACGGATTGAATCCGTCAGTGGCAAGTCCTAATCTCACATTTCGGGGATCAGC

AGCAAACTCGGGGAACTTTCGATCGAACTCTTTCCATGCCTCCTCATCTGCAGGATGCCG

CATTACATCATCGTCTACCCGTTTTTCCTTATGCCATCTCATGTCTGTTGTAGTGTGCGT

CGACATGTACAATCGCTGCAACCTAGGTTTCAGGGGCAGATAACGCATCACTTTTTGTGG

GATCTTAGTCGTTCTATTCTGAGATGTCATTTTGAACCTCGACTCATTGCATATAAGGCA

TGTATCCAACATTTCGTGCTCCTTGTAGAATAACATGCAATTATTTTTGCAGGCATGAAT

TTTTTCATAACCCAATCCAAGACCATTCAACACCTTCTGTGCCTGTTTATGATCTTTCGG

CAAACAATTGTCCATCGGAAGCATTCTTTTGAAAACCCCCAAAAAGTAATCGAAACACAG

GTTCGACATACGATACTTTATCTCTTCGTGCATTAGCTCTACAATGGCCGTGAGAACGGA

AAAGCTCTCGCACCCCGGGTATAACTCTTGGTTTGCATTTTTTAATAGTTTTTCATATTG

TTCAAACTCCGCGCTGTCTATTGGTGTTGGCACGTCATCTTCCCTTTAATTATTGATGTT

CGTCGATGCAAATGGAAAAACATCCTCTATAATATCCATGACTTGATCATTATGATCCAC

AATAGGTTCAACACTGTCCATTCTTGTGGCATTTGAAGACGAAGCATGGTCTAATTGTTC

CCCATGAATGCTCCAAATGCTATATGTCTCAATCATTCCATTCCTTACTAAATGAAATCC

AACATTTTCAATTGTCTCCCACAACGTGTTATTGCACCTCCTACAAGGACATCGGATTCT

AGTTGCACCCGGGTTCTGTCTACGTGCAAACTCAATAAAATCCTCGATTCCATCCAAGTA

TTCGTCTGAGCATCTATTCGGGTTCTGCTCATAATTCCTGAAACCATAATCACAACATAA

CAATCATACGAAGTTATAATGTCACCTTATGCCTCCGGTAAGGGCTCTATCCCATTCGGG

AATGCATAGTTCTATCACGTAACCTACGGCTACATGAGATTAGATTTCAACATGGATGAA

TTTCGGCAGCATCTCGATACAGTTCTTGAGGTGGGCATAGGTATTAATACCTACGTACCA

CCCGAAGAACGTACCGAGATGACACCGAAATCTCCACAATCGAAACCTAATCCTGTAGCC

GTGGATTACGTGACAACACTATGCATTACCAAACTGTCCAAATAGTGGGACAATTCAAGA

ATTAATTGGACCGCAGTCATGCTTTACGCATCCATGCACGAAATCCAACTAATCTCGAAC

GGGAAACTAAATGTTACTAAACATACAAATAAAAGTACGAAGTTAATTTCAATTAACTTC

GTACATCACAACACATTGTGCTACATTACACACAATTTAACAACATAATATAAATATAAT

TTCAGAAAAAATTAGTACACATAACAAACTAACTTTAACATTTTTAATATAGAACACAAA

TAAAATACCTTCTCAATCGTTCTCCACCAATCGCCAATTACAAATATCCCTAACGAGAAG

AAAATTAATAGCGTCAGTACGAATTTACATTAATACTTAAAAAGAAACGACAATAAATAC

TTACCCGATGAACGTACTGAATTTCCAGCAGAGACAGCGGGAGAGAAGTTGAGAGAGAGG

CAGAAAGGAAATTTTTTTCCATTCTGCCTCTGCAATGGCAGTGTTCTGATATGATGAAGA

AGACTTTGCGCGACGAAGAACACAGTTCGTCGCGCAAACTACCGTCGCCCAAAACCTTTT

TTCCGTCGCAAAAATCGAGTTTTTTCGTCGCGTGAAAACCTATCGACAAAAAAACTTTTG

CGCGACGGAATATATTTCCGTCGCACAAGATTTTGTCGCGCAAATTCTTGTCCTCGTCGC

CCAAAAACAAAAATCCGTCGCGTAACAAATATGTTTCGTCGCGCAAGATTGTACCGCCTT

CTCTTTTACGCGACGAAATATGTTTCGTCGCGCAAACTAAAATATCGCCCTCAATTATTT

TGGCGCCAAATTAAAAAAACCCTTCGTTTTCTTACGCGACGATAACCTTGTCCGTCGCGC

TAAGTTCTCTTTTGTGACGAAAAATGTCGTCGCAAGAGTTTTCGGAGCCAAATCACCTCG

CTTTTACGCGACGAAGTGCTTCATCGTCGCGCTATGTTTTCTTACACGACGAGTGGTCTC

GCCGCGCAAGTATTTGGCGCCAAGAGAGTACCCGGGGTTTTTTTCTCCTCTTTGCGCTAC

GAATCTGTTTTTATGTCGCGCTAATATGTTTTGCGCTTCGAAAACTTTAGTCGCGCAAGT

TCTAAATTTTTTGCGCGACGATTATTTCTTCTCGTCACTTTATTGCGTTTTGCGCGACGA

AAATATCTTCGTCGCGCAAACTTCTGTCGCGCAAATACTAAAACGTACTAGTGGGAGCAG

CTGTAGTCACCTGAATTTGGCTAGAGATGTTGTCTATCATTGTTTCTTCATATTCCTCTT

CAGAGCTTCTGGGAGTATCTTCAAGCCTCATTGGAATTGTGATAGAATCAAAAGTTTGTT

TATCCCAATCCCATAATGAGTTCTCATCAAAGATCACACTTCTAGATGTAGTGACCTTGT

TGGTTTGCAAATTGTACACTCTGTACCCCTTTTCACATTTGCCATAGCCAACAAAAATGC

ATTTTTCAGCTGCATCCTCCAGCTTAGATCTCAACTGACTAGGCACATGGCAGAAACAAA

CAGAACCAAACACTCTCAAGTGCTTAACTCCAGGCTTTCTTCCACTAAAAGCTTCAAAAG

GAGTGGCATTATCTAAAGCTTTTGTGGGACATCTATTCTGAATATAGACAGCTGTATTCA

CTGCCTCTCCCCAGAATTTATATGGTAGTTTCTTTTCTTTCAGCATTGTCTTGCTCATTT

CTATAATGGTCCTGTTCTTTCTCTCAGCAACTCCATTTTGCTGAAGTGAATAGGCCACAG

TTAGTTGTCTTTATAAACCAATTTCTTCACAAAACTGCAGAAATTCAGTGGAAGTATATT

CTCCACCTCTGTCACTTCTAAGTTTCTTGATTTTGTACCCGCTTTGAAATTCCACCATAG

CCTTAAATTTCTTGAAAATATTAAACATTTCTGACTTAAAGTGCATGAGATACACCCAAC

ACATTCTAGTGTAGTCATCAATGAAGGTCAAGAAGTATTTGTTTCCACCTATTTTTGTAG

TCTGCATTGGCCCACAGATATCTGATTGAACTAGTTGCAGAGGTACACTCGCTCTCCAAG

CTTTCTCTTTACCAAAAGCCTCCATGTGAGATTTTCCAATTGCACAGTCTTGACAAATTC

TGTCTGCATTGCCAATTTCAGGCAATCCATATACTAGCTCTTGTTGTTGTAACAACTTCA

AACTCTGCATATTCAAATGACCAAACCTCCTATGCCAATACCAAGTGGATTCTTCAACTG

TTGCTTTCATAGCCACAGAGTTTGCATATTTTAGTGAGAGTGGAAAACATCTGTTTCCAG

TCATGGAAACCTTTGCCACCAAATTTTCCATTGACCTGTCATCAAATATGTCAACCACTG

AATTGCCAAAAACCAGATAGTAGCCATGCTCTACCATTTGACCTACACTAAGTAACTTTT

CATCCAATCCTGGTACAAGCATAACCTCTTTGATATATCTAGGCCCAGGTTTAGTGTCAA

TCACCAAAGTGCCTTTACCAGTTGCTTGCACTAAGTCTCATGTGCCCATTTTCACTTTTG

CAGTGACCTTAGTATCAATATTGACTAGGAGAGATGCATGTGAAGTCATATGGTTGCTGC

ATGCACTGTCCACATACCACACACAATTATTTTTCACTATTTCAGCAGCATGACAGGTAT

AGAACATGATACCTTCTTCTTTTTTGTGTGATGCATAGTTGGCAGCTTGATCTGATTTTT

CTTTGCATTCCTTGGCTATATGCCCAACCTTCTCGCATTTGTAACATTTTGGTTTACCTT

TGAACCAACAGACACCATAATGCAGCTTGTCACAGGTTTTGCAAGGAACCTTTCCTCCCT

CAGTTTGACTTTCCTTCTTGGAATTACACTACTACAAAAAAGCAAACAGACGACGGTAAA

TCACCGTCGTGTATTAAGATTTTCAGTGGTCGTGGAATCCACCGTCATCTTTTCCATCAT

AAACCACGACGCTAAATCACCGTCGTTGTTAAAATATACAACGTCATCAACAAACACAAA

ACGACGTCTTTGTACTGCAAAAAGAACGAAAAAAGAAGTCGGGGATGAGAATAGACGACC

AACTCTCTAAAAAAACCGTCGTTAAAAAGGAAAAATATGACACATATTAACAGAACAAGG

CCGCAAGATAGACATACAACGACGAAACATAAAAGAAGACGCCGTTCAATATAATTTTCA

CGACAACAAAAAATGACATCTTTAAGAACATTAGAAGACGACGTTATTGATTCCTACTAC

GTCGACCATATAAAAACAGCCGTCGTATAATACATTTTCCACTTCGACTTTTCTATAAAA

GATGACGTGAAAATAGTTGTCAGTGTCATTATTTACGACGTATGTATTTATAGACTAGAC

GTTGAAATCACAAACAACGTCGGTTACGAATATATGAGAGTCGTAGTACAAAATTTTTAC

GTCCTATTTTCAGAAACAAACAACGACGATAAATATTAGCCGTTGTTAAATAAAACAACG

TCGAAAGAAAATAAAAACAGACGTGTTTAGTCTTCATAAGTTTTAAAAAATAGTCGAGGA

TGAGAATAGACGACCAACAAAAACAAATCACCGTCGTTAAAAAGGAAAAATATGACACAT

ATTAGAAGAACAAGGCCGCAATATAGACATACAACGACGATAACTAAAACAATACGCCGT

TAAATATACTTTTCACGACAAAAAAAAATATGACATCTTTAAATACATGAGAAGACGACG

TTAATGAAAAATACTACATCTCTTATGTACAAAAGACCGTCGTGAAATAAGTTTTCCACT

TCGATTTTTCTAAAAAAGATGACGTGGATATAGTTGTCAGTGTCATTATTTACGACGTAT

GTATTTATGTAGTTGACGTTGAAATGCGAAACAACGTCGGTGTAATTTATATAGTCGACG

TTGTATTTATATGTATTCATTACTCACGACATACTTATTAATGTAGACGTCGGTATTATG

GGATTGGAGTCGTGTTATTTTGGATTACATGGCGGTTCTTAATCCTTCCCCGACGTGATA

TCCTGAATAATTGCTTCACAATAGTGTCGTGGTTCTTCATTGTTACGTTGCTTTAAGACG

TCGGTAAATATGCTGAATAAATGGTCAAACATAACGTCGTGTTTTATTTGAACAGAGGTT

TTTTATGCGGAATCTAATGATGTAATCTGGATCCAGTATTTTTTGCACTGATTGTTTTGT

GGCCAAAACCTGTATAATGAAAGTGAAGAAATCACATTACAATATATTATAAAATGAATG

TCATACACTGCCAATTACATAAGCATCATTCAATCCATATATCTTCACACCCATCTCGAA

TATTTTGACCACCTAACTCACCCACCAGTTCATCAAATGTGTCAACATTTGGCATCCCTC

TAGTAAATGGCTCACACTCAATTATCACATCACCTAAGACTTCATCACCAATAACATCAT

TATATTCTCTATTAGGCATTGATAAAACTACCGACCAACCACGATGCATCGGGTCGTCAA

CAAAAAATATTTGTTTGACTTGAGAAGCCAAAACAAATTGGTCATTCCTATGTCCAATTT

TACTCAAATCTACATGGGTAAATCCAAGTTCGTCGACTACAAGACCAGAACTATCTATCC

AATCACACCTAAAGACTGGGATTGTAAACTTTTGGCAGTCAAGGTCCCAAATTTCTTGAA

TGACACCATAGAAACCCATATTTGAGAGAATTGGGTTTTTATCCTTGGCACTAGCAACTT

GCATGGTTTGTGCAAGTAAATAAACTCCACTATTTTGAGTTGTCCACACATCATCTTGTG

CCTTGATATTGAATTTAATACCTTTAATAAGATAGCTCCTATATAATGGCACTGCCATGT

TTGGACCAGCTGCTAGCCACCTTAAATTTTCTGATACGCCATTATTGTCTTCCTCAGGTT

CACTTTGAACCTGCAAATTAATCAAATCTTAATTCAATCTAACATAGAAATCGAATCAAG

AAAATTAAAAGAGAAATATGTTATTCAACAACATATACCTTGAAGCGTAGCCATTGAATG

AAAGTGCTATTGTGCTTATCCTGCAGCCACTTTGTTCTCTTTCTAAATTTTGGATAAGCA

GTCTTGATGTGGATCATATGTTGCCTACATGACACGAAAAATTCAAGCATTACGGTCCCA

AATATATAAGATAAACATAAGAAATAATTTAAAATGGAAACTTAAAGAATAAAACTCATA

CGTACTCGATATAAGGTAGGACTTCCTCCGTATTCTCCAAGACATATAGATGTGCTTGAT

TCAACAGGTCCTGATCAACTACGCTCACTGTGCAACCTGATAATGGCTTTGAAACTCCCA

TCTTTTGGCTTGAAGGCACTCCAACTGTACTAACATCAGATAAATGCTGAGTACAAAACT

CTACCGCTTCTTCAGCTATATACCGCTCAGCAATGCAACCTTCGGGACGAGTATGATTCT

GAACATACCCCTTCAGCACTTTCATATATCTTTCAAACGGATACATCCACCTAAAATATA

CTGGCCCACATAGACGAACTTCTCTGACAAGATGTACTACTAGATGAACCATGATATCAA

AGAATGAAGGGGGAAAGTACTTCTCAAGTAAACACAGAGTAACTACTACATCTTCTTCCA

ACTTATCTAGCTTGGAAACATCAACAGTCTTTGCACATATAGCATTGAAGAAGAAGCACA

AACGAGTTATTGCATACCTTGCAGGCTTCTCCAAAACAGAACGAATTGCCACAGGGAGCA

ATTGTTGCATTAAGGTATGACAATCATGTGATTTAAGGCCAAGAAGTCTTGAATCTTGTA

AAGATACAAGATTTTTAATATTTGAAGAATAACCTTCAGGGACCTTCATACCATAGAAAG

AATTACAAACCTCTCTCTTCTCTGCTCTTGACAAATTCCAAGGCCCAGGAGGCAAACGAG

TACGTCTTTCTCCATACTTGGGTTGCAAATCAGTTTTGACCCCCATGTTCAATAAATCTA

ATCGAGCAGCAATCCCATCTTTATTTTTTCCAGGGATCTCCAGCAATGTACCAATGATAC

TATCGCAAACATTCTTCTCAATGTGCATAACATCTAGGGCATGCCTCACAGGAAGGTATT

TCCAATACTCGAGATCAAAGAATATTGATTTCTTCTTCCAACAAACTCTGTCACCATTTT

CAACCATATGCAGCACTTCTTCTCCGGTTAATGGCTCGGGAGGTATGCCATATTCAGGTT

TCCCATTAAAAGCTGCACGTTGCCTCCTATATGGATGATTGATTGGTAACCATTTTCTAT

GCCCAATGTAACAAATTTTGTGGCCATTTTTCAACCTGTGACTAGGTGTATCATCGCCGC

ATATTGGACAAGCTTTATATCCTTTAACAACACAACCAGATAAGTTTCCATAGGCGGGGA

AATCATTAATTGTCCACATTAATGCAGCTCTGAGTGTAAAGTATTCTCCATTATGTGCAT

CATACACTCCTCTAATCCCAACCCACAAAGATTTTAAATCATCAATCAAAGGATCCAAGT

AGACGTCTATATCATTTCCGGGTTGTTTAGGACCGGAAATCAATAAAGTTAACATCATGA

ACTTTCGTTTCATGCACAACCATGGAGGGAGATTATATGTAACTAAGATAACCGGCCAAC

AACTATATCTGCTACTTAGAGAACTGTGGGGATTGAATCCATCAGATGAAAGAGCCAATC

TCAAGTTTCTCGGCTCATTACCAAACTCAGGCCATTTATCATCAAGAAGTTTCCAAGACA

GGGAATCTGCCGGATGAGACATCTGACCGTCAATTGATTTTCTAGCAGCATGCCAAGTCA

AACTCTTAGCTGTCTCATGTGATTGAAACATCCTTTTAAACCTTGGAATTGGAGGAAAAT

ACCACACCACCTTCGCTGGCACACCCTCTTTCAAGATTGAATCTTTGCCTTGCTTCCACC

TTGAGATACCACAAGTAGGACAATTAGTTGAATCCTCATACTCCTTCCTATACAAGATGC

AATCATTGGGGCATGCGTGCATTTTCTCATAACTCAGCCCCAATGCACACAAAGTCTTTT

TAGCCTCATACATAGAGGTTGGTATTGTATTTCCTTCTGGAAGCAAATCGCCTTGAAGTA

TCAATAATTCTGTAAAACAGACATCACTCATCCCATGTTTTGCCTTCAAATTATACAACT

TCACTAATGCCGATAACTTCGTGTACTTTCTACAACCAGGGTACACTGGTTGATCTCCAT

CCCCAATCACATTGGCAAACTCATACGGATCGGAACCAAAATCACCAAAATCACCAAAAT

CATTATCATCCATATCAATTTCTTCAGACACAAAACTGTACCTACTATGGCCATCTTCAA

CATTTCTACTAGCATTAGTAGTTGCTTCCCAAGGTTCTCCGTGAAATGTCCAATTCTTAT

AGCTTTGGTCAATTCCATTAAAGTATAAGTGATCCCTTATAATTCCAACCCCAAACACCT

TCAAATTAACACATTTAACACATGGACAACGGATATGTGTTGTAGTTAGAAGATTTTCTA

CAGCAAAGTTCAAAAATGCTTCCACCCCAAATTCATATGCCTTAGATCTTCTATCCGAGT

GCATCCATGACTTATCCATCTCCAACACTATAACCTATTATCTATAATAAAGTGAAAATA

CAGGCAATGACCATCACTATAGCAGATTATACAAAGATTTAATAGCAAGTAATACACAAC

AGAGACGACGGGTTTTAAACAAAAGCAGACGTATTTGGCATATATAGACGACGGATTTTC

AACAGACGTCGTCTATTATAAGTAATACAAACGACGGTTTATGAAACACCGTCGTGTATA

AGTAATACAACGACGGTAGTTTAACATTCGTCGTGTAATCGTCGTCGTATGCCTTAAAAT

TCGTCGTCTCAGTTTATATTCAAGAACACAAAACCCATTAAGAACACAACATATATATGA

AACCAAGCAAGAAACCAACATATACATGAAACCAAGCAAGAAACCAACATATACATGAAA

CCAAGCATGAAAACAATAAATCCATTCCCAATTTAACATAACATAGGGTGATTAAAAGAT

CCGACAAAATTAAATCCATTCCAAATTCAACATAACAGATAATGTAATCCATGAACCCAT

TAAACAGAAAATCCATGAACCCATACCTATAAGCACATTATTAACTGATCGCAAAGCATA

CAACCTAATATCATTCAATGAATTTACAAGGGGAAGTTAGGGTTTGTACTTACAGGTTGG

ACGAGCTCACAGATTCGACGGAGCCTGGAGAGTGCAACTTTTAATTAGAGCAGAAGTGAG

AGAGCGAAATGTTATGTCGCGCGAAATCTGAAATTTTAGTTTTCAGGGTTCGAAGTATAA

GAATAAGAGCCAAATCTAAAAAATTTAGGTCGCGCGAAAATTTTTATAGCGCGCGCGTTT

TTAAACGTCGAAAGAAAAACCAAGGCGAAAGTTCTACAAGGACCGACGTAAATTTAATAT

TCCACGACGGAAACTTCATTTTTCGGATTAAGAACGTAAGGCCGTTCATTTTGAAGCTTC

ATTTTTCGGATTAATTTTAAATTTATTTTGAATATTTTTATATAACGACGACTGAAAAAC

CACGTCGGAGTTAAGACATTAATGACGTTGTAAATTTTATAAACCGACTTGCATATTAAA

ATGACGTCGTTTAAAGCATAAACCATGTCGTATATTTTACTTCACGACGTAAAATACGTA

TTCCACGACCCAACCATCGTCGTCAAAAATTAATACTCTAAACCCACAAAACTAAATTAT

ACAATTTAAAAAATTAAGTTTATAAAAGGTTTTATGGTTTTACAGCCTAACATATACCCT

AGACTACCCAAAAATTTTACTTTAAAAATAAACCCCAATAAATAACAACCCTAATCTCTA

AACCCTAGACTCTAAACCCTAAACCATAAAACTAGAAGTGTTTTTATGACTCATCAAAAC

AATTTGGTTTTGGATGGTCATTTTAAATTTTTGTTATTCAAAATGAATTTTTTCAAGGTA

TATGTGGAAGAAAATATATCAATGACTTCATAGGAGTTGACTTTTCATTTTTCTGATTTA

TTTTTAATTTATTTTGAATATTTTGATATAAAAATAATAAAATATTCTTATCACAACAAC

AAACCACGTCGGTGTTAATAAATTGAAGGCGTTGTAAATTATAATAGACGACTAACATAT

TAAAATGACGTCGTTTAAAGTAGAAACCATGTCGGTATTATAATGAACGACGTAACATAT

GTATTCCACGACCCAAACACCGTCGTTAAAAATTAATATCCTAAACCCTTAAAACTAAAT

TATAAAATTTAAAAAATTAAGTTTATAAAAGGTTTTATGGTTTTAAAGTCTAACATATAC

CCTAGACTACCCAAAAATTTTACTTTAAAAATAAACCCCAATAAATAACAACCCTAATCT

CTAAACCCTAGACTCTAAACCCTAAACCATAAAACTAGAAGTGTTTTTATGACTCATCAA

AACAATTTGGTTTTGGATGGCCATTTTAAATTTTTGTTATTCAAAATGAATTTTTTCAAG

GTTTATGTGGAAGAAAATATATCAATGACTTCATAGGAGTTGACTTTTCATTTTTCATTT

ATTTTGAATATTTTGATATAAAAATAATAAAATATTCTTATCACAACAACAAACCACGTC

TGTGTTAATAAATTGTAGATGTTGTAAATTGTAATAGACTACTAAGACGTTAAAATGACG

TCGTTAAAATGAGAAACCATGGCGGCTATTTAACTATACGACATAAAATATATATTCCAC

GACTTAACCGCTGTCGTTACAAAGTACTATCCTGAACCCTTAAGAAATTGAAGATGTTGT

AAACCATAAACGACTCAATTGTTCAAAGAACGTCGTCTAACTATCCTTTGACGTCGTATT

TGTTTAAAACGAAACAACAAAATACGATGGTTTTTGACTTTATTAGGTCGTTGGAAAAGT

ATTACACGTCGGTTACGCAATTTGTATGTTTGTTTGTTTTTTTTAATTTAAGAAAACCTC

AACAAATTTTTAGATTACATATTATAGAGAAAATTTGTACGTTATACATAAATATCAAGT

GTTCAATAATTGCCCTATTTAATAAATTGGAAAACAGTCTCTGCCCATTCATTCCGGACT

TCATCAATGGCTTCTTGTGGGTATGAAGCTTCCTGGTTTCCCTTGGCATACTGCATAAAC

AATGACTATTAGGGTTTATCAATAAAAAGAAATTCAATCACAGACGACGATATTTTTCAT

AACCGACGTAGTATATCTATTCAACGACGGTAATTTTACATGACCGACGTTGATAATACA

AAAAACGACATGAAATGTATGAATGTAATTTCTTCTTACCTTATTCTCAAACCCCAAGGA

AGGATCCATGATGATGTCCCTCATGAAGCGCATAACGTAATACCCGCATTCGACACTGCT

GGGTTGCTTTGGTGTGCCTGAGAGAGTTTTCCAAATTACATTTTTACGTCCAGTTCGGGC

TATGTGGGTATTATATATTTTTATGGCACTGTTCACGATGTTTTTCGCCTCCTCGTCGAC

CACACGTTGACCTGGCAGAGGATCCAGAAAATAGACGGTCTCCTTCTTTGCTCTTACAAT

CAGCAAGATCCAATGGCGGCTGCACAAAATTAATATAAAAACGACGGTTATTTACAAAAA

CGACGTATTATAATAGTTCACACGACGAAATAAAGTAGAAAACGACGACATTATATATTT

ATCACGACGACAAGTAAATACCGACGTGGTTAGTTGTTTCAAACGACTCAATAAAATAAA

ACACCGACGTGGTTAGTTTATACGCTGTCATTTATTGAAAATCATAAAAACAAAATCCCA

AGGAGACTAACCCTGGATTGTAAGGCATCATGAAAATCTGTTCACCGTCTGTCTTCTGAA

GTCGAGCTGCTACCAGTCGTGATCTGTCAGCTATTGTGCCAGAGTTGGCACTGACTGTAG

CAGGGTCGATAAAGCCAACCATGCTGCACATATTTGCTTGTTTCAAAACATCGTGTAAGT

GCCTAAATAAATAAAATAATATAAACAAGTCAGCACAAAAAACCACACGACGGTTATTAA

TAACAAAATGACGTATTATAATATTTAAAACGACGTACTGAAATAGATGAAGACGTTATA

ATATATTTATCACGACGGTACACACTAACGACGTATATAAACCAACGACGTATTATTTTA

GAAATACGAACCTCATATATACAGCAACCACAGTAGCTCCAATTTCTTCCATGCCTGCAA

ATTGTGTAATATCTTCAGGCAGGAGGAAGGTCTCGCGATCACCACCAAACACCTCCTTAT

CAATTGTAAATTCCAGGATCTTATCCTCAGGCAAGAGTGTCGTTTCCACATAACAACAAA

GGCTTTTTAAAGAAGATGGCGCCTCCATATTTGAATAATCACCAACTTCATTAACCTGTT

TAAAGAAATGAAAAAATGACGTGGTAATTCAATAAAGACGACGGTTTAAGGAATAAAACG

TCGTCTGAAAAGTATTTAAACGACGGTGACAAAACCGTCGTGGTAAATGTTTTATTACGT

CTTAAAACAATTCCGCCGTCTAAGTTGTAAACCAACGACGGATTAAAGAAAAATAACGAC

GTCTGAAATTTTGAAAAACAAATAAAAAAGGCAAAATTATGTGAACATACCTTGTCGTCA

TGCTTTTCTTCATCTTTTTTCTCCTTCTCTTCTTCCTTCTCTTTTTCTCTTTTTTCTTCT

TCCTTCTCTTCTTCTTGCTGATGTTCCCCATTTTTGGCTTTATCATCCTCATAATGGAGG

GATTTCACCTCACCTCCAGAGCAGCTAGCTTTGTCAGACGACATAGGATTTTTGGGGCTT

TGGCTAATTCTTTGTTTAAGCATGCCTGGATCAAAATTAGGAATTAATTGGGAAAGCTGA

CTAAGGAAATGCTCCCTCTCAGCCTCTACCAATTGTTTTGTTCTAGCCTCCATCCTTAAG

GCCTCTTCCTTTGCCTTAGCCTCCATCTTTTTAGTCTCTTCTTGAAGGAGAACTCTTAAA

CTCTCCTTCAAACGGTCGTCAAAGCTAACCCTCTGTGGTTTGGGTAAATTGAAATACTGC

CTTGGGGAAATCCCAGCACCTACTCCTCTCAATCTGCCAGGATGCTCGGGGCCCAAAGCC

ATGGTCAGCACATCTTTGCTGCCATCTACTCTGACTTTGCCTTCGGAGACTTGTTTCTGC

AATTCATCCTAAAACAAAGATATACAAAAAACACGACGGTTATTTATATACAAACGACGT

ATTATAAAATTTCACACGACGCAAAAACATATAAACCGACGTTATTATAACTTATCACGA

CGGTAGTTATACCACCGACGTCTTATGCTATAATTACAGAAAACATAGTGATTCCTATTT

AGACCTTTAACGACGTTATTTAAAAAGTGTCGACGTGGTAATACAATTCACACGACGCTA

AAATGAACCACCGACGTCTATAATTAAAGAAAACAGAGTAGTGATTCCTAATTAGACCTT

TAACGACGTGTTTTAAAAGTTTCGACGTGGTAATATCATTCACACGACGAAAAAATATAA

CATCAGACGTTAAAAGAATTTTTCACAGTTTAATAAAAATTTAAAACAGAGTGAGTAGGC

TTACAATTAATTTTGCTTTTTCTGCCACCTTTGGATCGGGGATGTTACCATGTTTGTCCT

GTCTAGCTCTCTTCCATAAGGTAGATCGATCAATTTCTACCCCAGGCATGGTTTCCTCCA

ATTCATCCTCCAATCCAGCATATCCTTTTCGAGACAATCGATGATTGTACTCGAGTTTCT

CCCTAATCTGTGCATGTTGAGAATGCACAGACTCAAAATCTTGGGAAAGCCTTGAAGCTA

CAAAGGCATCCCACTGTGCTTTCTCTATGAATTTATAAGTTTCCGGGGGATGGCTTAATT

TCTCCTTGTCATTGGTGTATGGAAGGATATAATGCCTCGTTAGTGTAGACTTGAAATCCT

TCCATTTCTTGGCAGCTGAAGCTAAAACAGATTTCTTGCCCCCTTGACCTACGACAAAAG

CCATGTCAACTGCCTCCCATATCTGCTCCTTAACATCCTTGGGGATTTGGGACCATTTCT

TGTCCACAAGGGGAACTCTGGAGCGAGCCAAGACACCAATGTACGACTGCATTTCAATAT

GTGCTTGGCCAATACCTTTCCCCATTTTATTGTACTCAACAATTGGCCTCAGTTTCTGAA

GCTTTCTCTTCACAACACGAGGCATTGTGCTCATACCTCGACCAGTACTCTTTGAATCAT

CAGAGATTGTTGTCTGGCTGGTTGGTTCTGTCTCAGCAGATGATGAAGCAAACTTCATCT

TCTTCGAAGACTTCATCTCCTTCGAAGACTTGTCCTGAGGAGCTACCATTCCAAGCTTCT

TGGAGCCAGAATCCTTAGTTTGTTGTTGAGAAACCATTTTAACTGACAAAACTGCAAGTG

AAAATATTTCAGGAAAAGATTAAGAACACAAACGACGGTTATTAATAAAATACGACGTAT

GATCTGATTTTAAACGACGCAATGAAATAATCTCAGACGTAATAAATGTTTAAAACCATT

ATTTATATAACGTCGTGGTACTTATGTTCACACGACGTCAATAGTAATACACCGTCGTGG

TAAAACCATTAATTATATAACGTCGTGGTATTGATTTTCATACGACGTCAATAGTAATAC

ACCGTCGTGGTATTAACATTCACACGACGTAAAACAATAAGACAGACTGTCGTCTATATT

AGATACGAACCCTAGTTTCTTTTTCTTTATATTTTATCAAATAAGGGAAAAACAAAAACC

ATTGTTCAGAGAAATCAAACCTGCAATTAAACAAGCAAATAAAAAAAGGCTTTATATTAA

AAACAACTTTGTCAATTTACAGACGACGCTAGAACGACTGACGAACCGTCGTGGTATACA

TATTTAACCACGTAAATAAGAACTACCGTCGTCTTATTTAGTTGAGGTAGTTGTCTTCAA

AGTTGGAAACTTTCCCTCTTTGTCTGTATATTTTATCAAACTTGGAAAGAAGTAAAATGA

TTTTGGCCAGAAAAAAGGTAAAAAGATTTTTCAAACAAAAAACGTATGAGCATGCAAAAC

AGTAACCAAAATAGTGAAAATGAAAGCTTACCTTTTGCGTGCAATGAAAGCAAGAGGAAG

ATGATCGATGTTTAAGTTCAGAGACGACGAAGAAGACGAGAGGAAGACGATGAGAAACTC

TGACAGTGCTCTGACAGGAAAAAAGACGAAAAGTTTTCTCTGGGAGATTGAAATTTTTAA

TCGGGTTTGGAAACAGTGCATGTGTAAGTCAAGTAGACGAAAAGTTGCTTACATATAAAA

ACATTTCAAAAAAATAATACGGCAGTTACATATAACTACGTCGTTTTTAACTAACACGAC

GCAATATTTGTTTTTGAGACGTGGAATCGTAAATTTAACGTCGTCAGGTTTAATATAACC

GTCGTTAGTATTTTAATTTTTAAATTATGAATAGAATTTTTTTTCCCGTGACTTTTCAGT

TTATTTTTCAATTACAGTGCGTTATAAATAGAGTTTTTTTTCCGGAGGAAATTTAAAATG

AAGGAAATTAATATTCAGGAATATGTAAAGTTTCTTTTTTGGATGACAGATTTAAATAAA

AAAAGAATATAAAAACAACGACTGTTTTTGTTTTAATGTCGTCGTTTTTAGTCGTCTTAA

GTAAGACTAAGACGACGTGGATTTATTAATTTACCGACGTTGATTGTAAAATTTAAACGG

CGGTTTTTGGTTCGGACCGCCGTTGGTTTTAAAAATTTATTCTATAAATTTGTCGCTTTC

ATTCATTTTCGCGGTTAAAATTTAGGGTTCCAAGTTCTTCCTCTCTCAGAGACACTGTCT

CTCACATCTCAAAGACAATAATTTGGATGTCTTTCTCAAAGAGAAATTGAGCCAAGAAGA

AGCTTGTCAACTAGCCTTCTCACTTCCTTGATCAAGCCTGATAGGACACTTAGTGCTTCT

GAATACTCCTTGCTTTCCATCAAAAGAGATGCAAGCCTGGCCTCCACTCGCTGTCGAAGG

AAAAGTTCGCTTCTCAGGGAAATCTGAAGATCAGATGTGCCTGATCCTCTGCTTGATTTT

CTTTACTAAGAAGATCCGTCAGATTTGTGATAGCCTCTTCCTTTATCAGTAGAGCTTCAG

AATGCGATAAAGAATAGAAATGGCTTCAGATGGCCTCTAAAGCATGAGCAATCGAATCAG

TAGTTGCTGGGAGATATGATGAAGACATTGTCAATGCTTTTCTCGTCGTTTATATTAAGG

TAGATTTAAAATTACCGACGTAGATTATTTTGTTAAACGAGAATGACGTCGATATATTTA

TATTTTCCGTCGTTGTACATTAATTTAATACGGCGATATTTTGTATTTTAAAGCCGTGGA

TTTGTTTGTAATTATACGGCGTCTTATACGAAAACCTCGTCGTGTTATTTTATGAGTAAA

AACGGCAGCTTTTATTGAAATCGTATCTTTACTTTCTACGTCGGTCGATTCATAACTTCT

GCCGTTCTTTGTTGGCTTTGATTTTACACTGCGGAAATCTGTTTCAAATAGACGTCGGTT

GTTTATTTAGGTACGTCGTTGTTTTTGAACAGCCATTTGAATCTCTAATTTTTAGTTGTC

TAAGGTGGTATTACACGACGGTGTTTCTAGAACCGTCGTCTTTTTAAAAACCACGACGGT

TTTCACTTAGACCGTCGTTGTTTTCTGGTCGTCTTTTTCACTTTTTGTAGTAGTGTTATT

TTCAGACTTAGAGTCCCACTTCTTCCCTTTCTGAGATTTCCAATTCTTTTTAGACTTGAA

ACTCCCTGCTTGCCCTGTGTTTGATTGATCTTTAGAATTCACATTCAGACTATAGAATGC

CTTCTCAGTCACTCTCTCATTATGCCTTTGCAGCTGTTGTTCATGAGATTTTAAAGAGCC

TATAACCTCTTGAACCCCTATAGATTCTGTATCCTTTGTTTCCTCTATAACTTCAGCAAT

AGAATCATATTCTCTAGATAAACTAATCAAGAGTTTTTGTACAATTCTTTGGTCAGTTAA

CTCTTCACCATAAGCCTTCATTTGATTTACTAGCTCAAGTAGTTTAGTGACATATCCAGA

TAATGATTCATCATCATGCATACGAGTGTATTCAAATTCTCTTCTAAGAGCTTGAAGTTT

CACAGATCTCACCTTCTTGTCACCCCTGAACTCTTGTTGCAGGATCTCCCATGCTTCCTT

AGCCGACCCTTTCAATGCAATCCTGGGAAAAATGTCATCAGAAACTGTACCTTGGATCAG

TCCCAAAGCCTTAGCATCCTTTGCAACATTCTCTCCCGAGCAAGCTTTTGAGTTGTAGTC

AGAGCTTCTTCTTCTTCCTTCTTAACAGATTGCTCATATCCATTCTCAACCATATTCCAC

AGCTTGTGAGATTTGAAGATTGTGCACATCTTTATTCTCCAGAAATCGTAGGTACTTCCA

TTGAAGATGGGAGCACGAAGCTCACCCATGCTAGATCCAGCCATGTGAGACTAGATAGAG

CTCTCCTCAGCTGGATTGCTGTAATTTCACTATGCCCAGTCTCATATCGTAACCTGGACT

CTCGATACCATGTTGGAGTTTCTGTATATTAGTAAGTGTTCTTACACAGAAAGTATATGA

GGCAACGATTTAGAGAGAAAAGAGAGAGAGAGAGTCTGTTAGGCAATGATTGTTATCATG

CACACACACACTGTGTGATCTCATTCTAATTTCAATATATATTGTAGCTAATACAAAAAA

TGACTCAGCCAATACATTTGTACCAGCCTGTGAACTGCACATTTGGCATGAATCTGGACA

TGTTAGTCCAACAATATGTGATGGCATATGAGCCATACACCTAGCTAACTAACAATCTAA

AAACCTGAAACAATTTATACTTCAACAATTGCTTGAAAAACTATTTGAGGTACTCTCTCT

CTCTCTCTCTCTCTCTCTCTCTCTCTCTCTCACACACACACACACACACACAAACACACA

AAAAGAATGACGACTGAATTACTGATATCATGAGTACGTTTGTCCCTAGGGATTACAGCT

GGGCCTTCCGCATCTCTATGATTATGGTTTTATTTTTCCTCTACGTGAAAGATTAAAGTG

GTTTAGTGAACAAAGCAGCACGTCTGCATGCACAATAAATTTAGCTCTTCCTCAAAATCT

GCACAACGATGAGAAATGGGCAGGACTTTCGCTATATGTTGTCTGCAGTCTGCCCCCAGG

TGCTTCACTCACTACACGCACTTTCTATGAGATTCATTTGTATACCCCTATTGAAGTTGT

GGGCCATAACCAACTGATGCATCGCCTACAGCGGCGGATCTAGGAATTTTTCAACGGAGG

TGCAATATTTAAAAGCTAAGAGCTCAAAACCCAAAAAAAAAAAAAAAAAAAAAAAAACCA

AAAAAAAAAAAGACAACTAAACATCCTTATAATTTAAACAATACTAATATTATTCATAAT

TAATTAAAATAATAACATTACAATTAACCTACAATTGTTGCCGACGAGGTTTCATATCAT

GAAAACGTCGCATTATATTTGCATTACTAATACAAGAAAAAACATCTTTCTCAATGTAAA

CAACCAAGCTATCACTCAACCATTGATTCTCCATTTTATTACGCAATGGTGTTTTCACAA

TATTCATAGCTGAAAAAGCTCTCTCAACTGAAGATAACATATAGATGCATTTAGTTATTA

TTTAATATTAGCCTAGTATGTAAAAATAAGAAAATGAACCTTTTCTCAACATCTCTTTTC

TCCTTAGCTGAATCCAAAACTTTGGGATAGGAATCAGCTGTAAAAGATTGCTGATCTAGG

GCCTGAAGTTTAGATTCGATATATGGAGTCTTATTTTATTTAGTACTTTTATGTATCTTT

TTAGTAGCAAATACTTAGTTTAGCATTTTTTGTTAAATTTTCAATTTATTTAGAATTGGC

AGTTTTTATTAGAAATATCCTCTAAGTGGGCAGCAGCCTTTGGGCTTTAGAAAGTAGCAT

GGGCTACATCCAATAAGCTAATATCTTCTGCCCAGTACACTTAGCACATTATTAGTTCTT

CTTGGTCTTCCTCATCTTCTTTTCATTTTCATCTTGTCTTCTTCTTGTTCTTTGCATGGG

TTACTCAGATCTGATGATGATCTGATTCTTCTTCTCTCATCCAATTAACCCATCAGAGAA

ACTATGATTGATATGGTTCTATTGGGTTCGTGATGAAGGAGAAATAGAAGCTGGGTATTT

TTAATATGCAATGGTGAGAAGAGGAGCTGGGTTCAAAATCAGGTGGCATGGGGCCCTATA

TTCTCAGGCATCAAAGGCTTATGCCTCTTCCACCTTCGCGCAAAAGCGCTGCGAGGCGTG

CCCTTTTCCAGATTTCCGCACAACAGCCCTACGCCCATGGCATTTTCTGACAAATCAGGT

GACAGGTCCCAGCCCAGGTGGTTTGGGCATTTCGTCGAACAGGTGCTGTGCATCTCGTTT

CTGAAGCAGATGTGCAATTCCAGCTCTTGTAATGGACTTCTCTGGCGACCGGAGGTGCAG

GTGCACCTCTTTGCGCCTGCACAGGTCCGCCACTAATCGCCTAATGGTATCTTCTCCCTT

GGATGACAATGAGGGGTCACATCGGCTCCTCATTGTTCATCTCCAAGGTTCACAAAAAGT

GATATCCATCAAACTCCAATTCTTGTGCCGGGAATTTGATAATTTACTTATGAAGGACAA

CGAATCCATTCAAGCACTCTTCAAAAGTTTCTAGTTGTTAGTTTAAGGGAGAAGAGGTTT

AGAGAAGGGTTGGTAGTGTTTGGGACAATAGCTTAACTTGGGAAGGAAAACTTTTGAAAG

AGTAACAAAGTAATTTTATTTTATTGATTGAAATAGGAAAGGTACAAGGTTTTAATACTT

GCGTTGGCTGATGGCTAGAGTTGGACAGCTGTAGTTTTTTAGCTTGATTACATTGAGAGG

CTATAGGCTTATTTATAGGAATACTTAGTCGGCAACTTTGCTTTGGACAATTCGGTTGCA

CAGTAACCGACTATTCCAAAGTCATTTTCAGCCACAAATTGTCTTCTAATTCTAGATTTC

TTTAGTTATAATACATTATATAATTAGTAGGATGTTTCTAGTGACTTTAACTTGTCAACT

TCTCTCAATTTTTCTAGATTATTCTATCTTATTTAAGACAGAAATATGCATTAATTTTAG

GATGATACAATTCCAAACAAGAAAATCGTTGCGAAAACTTTAGGAAGCCTACTTATACCA

CCAAAATTCAATCATGTCGATGCCGCAATAGCATAATCGAAAGATTTTATCTACTCTTGC

TTCACATGAACTAATTAGTTCAGTGGAAGCACATGAAAAAGATGAACAGACAAATCCGTG

GAGCAAGCTTTTCAATCCAAAGTGAATTTCAAGGACAAAGTAAGACAAACAAAAAGGGGT

AGAATCCAATACAATGGATCGGCCCTTTCATTGCTTTAACCACAACGAAAATGGAAACCA

ACTAATTCTTATAGTTTATTGGTATTCTCTTATTCATGTTGGAAGAGGTCTCAAGTTCAC

ATCTCTCCTCTCCATTGCTAAAACTAATAATAGTAAATGTTACCATATGGTCTAAGTTGT

GCTTTGAAACTATGACTTAACGGTTGATTTGGTTTATCATTTTAGCACCATGACATGATA

AAATTTCATAGGTTAGATTTTTCTGTACACATTCATCTTTAACAAAAGAGACGAGCTGGT

CCTACTAAGTTGGTCTTCGAACCAGAAGGAGAAGGGCTTAAATTTTAGATTGGGTCTATG

ACATAAGAATGTCACTAGCTCTTTGGGCCCTATAATAGAGTTTGGATCCTACCTTATATA

GATGATAAGTTATTTCTGAAATTTCAATCGTCTTTATGGCTTTTTGGTGTGCTTGACTAT

TCTCCCCTCACCAGAAAATTTCTTGGAGATGTCGAAATATGCACATTTGTTAGTGCTAAT

TATGCGTTCGATTAAATGTCTCCAAGAGACTTTGTCCACTATGAATCTAAGGGAATTTTT

ATTGTTTAAATTTTAAGAGTATTTAGTTGTATAATTTTTTTTTGGTGGAACTTTTAACCT

TTTAAATATTTTCTAAAAAAATTCTGACTCTGCCTGCACCAAGTCCATAGCAGCTGCCAA

ATAGAAAAAAGCAGGCTAGTACCTTCAATATAGCTGCACAGCAGCATGTGAAATTATGTG

TGAATAGGACCTACCCAAACTGAAAAGCACCAACAACCAGATCTCCCACTTGCCCTGTGT

GGCCTCATTCAGTACTCAGGTTCCCCCACTGAACTGGTCTCAGATTCATACTTTCACATG

TATATGCTGCAACGGTCCTCACTGCAGAAGTTTCTCATATGGGCCATCTCCAACTTCCCT

AGATAAAATACCTAAATGATGCCCCAATTTCCCATGCCCCCATGTGCCTAACACAATTGG

CAGTTTATGGCATGTATAGATACTTGGACCTTATAAAATTATAACCCTTGGTTTCTTTGC

CCCACTAGACCAAACCAAGCCTGCAAATGAAGCATATATAGCACTGTGATCCATACTAGG

GACTGTTTCGGAACCCACATGAGGCTCCACTTCAAACCATGACAGCTGTGGACTTTTGGC

ACCTTGCCCTTTTCTATTGGCAAATACGCCTCCTCCCCGGGAAATAAGAAATCTGTCAAT

TACAGTTTGACATGTTTTTTTTTTTTTTTTTTTTTCGGGTGAAATAAGGGGAAATTCCCA

AAAAACAACAAATAAACACCACGCATGAGTCGAGGCCTACTAAGACCAAACAAGTCATCC

ACCAAGAGACCCAAACAGGAGCCTAATCAAAAAAGCAAATTTCCATGTTCAGATTATAAC

TCCATTTTGCCAAACAATCCGCAACACAATTCTTTTTCCAGTAAATGCGGACAATGTGAC

AATTTCCAATTTGCCTCCTGAGATTGCAACAACTATGCACCAAAGCAGCAAGAGGATGAC

ATTGCAATGAATTGATCTGTTGAACAAGTGAGACAACAACAACAACATTCATTTCAATAG

CAAGGTTAGTGATATCTTTCTCAACAGCAAGCTTTAGACCAAAATGTAGGCCCCAAACCT

CAGCCTTCAGAATATGACCTTGGGCCAAATTCATAACAAAACAGTTTGACAAGTTTGTAA

AAAGATAATTTGTGTGTTTTATACAAATTAATTTTGTATGCGTGTGAAACTGTAATTTTT

AAGAGCGAAGCAGTGTCATCTTTTTATTTTAAAAGCTCATTCCTAGGTCAATTCACAACT

GAAAACTACATTAACGGCCATTGTTAAATATTCTGGGAATCTAGATGATAGAAACGTCCA

TCAGTACACTTCTCATCTATCTTGATAATTGTTTCAAGACTGATTTTCTCTAGAATTTTG

TCCTTGATAGAATAAATACTCCAATACTTCTTTGCTGCGAATTTGAACCACTCAAAAGCT

AGTGGTGGCAAATAACCGCAACGTAATGTCAGCCGCCCACCAATAAAACCTAGCTTTTAA

GATTCTACACGGAATTATTTGGTGGCTCGTCTCGAACTAGATTTCAATCTATTAATTATG

TTAGATTGTTATCTAATTTAATAAGTCTGATCCTATAATTTATAAGCTCACCAAATAATT

GTCAGAACATTAGTCTAATTTAATTGAAATAGTCTAAGTAACTTGTATTAAATACAAGTA

TTGAAATACATTCTTGATCCCTACATTTTATTCGGATTTTCAATTTTGTATTTCTAAATT

CGGCTAATTTATTCTACGAATTTTAATCTGTTAACAATTCGGGTCATTTTTGCCTGATTT

TGTGCCCTCACAGATCGAGCAAATTAACGTCAAATGAGACCAAAATTGTTAACATGACAA

AGCATAAGACCAAGTTCGTCAATTAAACATGGGAACCAAAATAAAAATTCACATGGGTCA

AATATGGCATTTAACCCTAAATACGATGTCGACATAATTGAAAGTGTAGGGAAATTTAGA

CATCATGTTTGTTAGATTGCAGCTGGTTGATAGTTACATATGTATATGTAGGTGGCATTG

TGCATTCTAAATCTAAACAAAGGGACTTCCGAGATACAGCCAGTCCAAAACCCTCTGGAA

AGTTGGCGACTTATGGCAACCGTGTATCCACAGCTGTCCACCAAACACCAGCTAACTAAT

TTGACCCATTCTCCATTCAAAAGAAAAACTAAGCAAGCATATTGACCGATTCTTCCCTAC

CTTTATGACTAAGCGACCCATTCTTGGAGCTGTTTTGACTTTGTCATTGTATCTAGACAC

TCTTAAGTCCAACCTCGTGTCTCACTAATGTACTATGCCCTTGCTGTTCTTTTTCGAGTG

GGGAATTTAAACACGGAATCTCGGATGCAAGAATTTTTACTCTTAACCATCTGAGCTACA

AGATACTTGCAAATATTGTGAATTCCCAATTGTATACACCTTTACTAAGCCACTGTATTG

TAGTAATATGGTTATACAGGCTGATTTCTAGGCATTAAAAATGTAATGATTCACCATTAC

CACTAATATTACGGTTGCACAGGCCGAGTTCTAGGCATGAAATATGGTATGATTTTGCTA

CGATCAATCGCGCCTGCGTGACCAGGGAAGCTTTCCTTCATATACTAACTATTGCTTCAA

AGTTTTACTCTCTCCTTTGTCCGACCCCATGATATTATGGAATCCCTGTAAAACCCTCAT

GTTTGATGTTGGCGGCTACAAAGAACTAACTGCAGCAACCTGGTTTAGTCTGACTAAATT

AAATTCGGAGTAGTTTTCTAAACTGGTAGCTACAACAGTTCAGAGTAATGTTTAAGGCAT

CCTCGCGGATGGTTTTCTTAGCTCGATATTCAAATTTGCCGAACAAAACCATAAATCAGC

TCTCATTTGCCCATGTGGGGTCATTGTAATTGTTCAACATTGCTCCAAGTCTAAAGTCAT

CTCTGTTCTAAACAAGCAAAGAATGGGGGAAAAAAAAAAAAAAAAATCCAATCTATGCGA

TGATCTGACACAATCATATATGAAGCTTCATCACCACTTTTTCTTTAAGACTAAAATTGA

GAAAGATTTGAAAATACGAATAAAGAAATCAAAGAAGTCGGTCGCTATCTGGGTTGAAAT

ATTTGGCTCAGTGATTGATACAAGCCAAACCATCCAAAAATGAGTCCATGGTATAAATGT

CTAGCCCTCTAGTCCGTCCAGGGGCCCCTCCTTTGAGGGATGACACATCCAGCACAATCC

TGCTAAGACAGCGTAAACGTGTAAACAAGCTGAATATCTTATGCATAGAAGAAACTAACA

ATGAAAAAGATACTTACATGTTCGTAGATGCTTTTCCCAGTTCCGGAGTTTTAGAGTTTA

TGATGATTCTGTGATCAATTTTGGCTACTGAATGGTCCATAGGCAGATCAACCACTTTAG

GAGCTGAAATGGAATTTTTAACACGTTAAATTCAATTCAATTAATTGCATATGAAACTAA

TAAGAACCAAGTATACAGTAGGACTGAATTGAACCATTTCTTACATGATTTAGGAGGAGC

AGTGACAGTTCCATCTAGCCCCAAGAGTTTCTACAAAAATGAGGGAATATGTTATCACTA

AGCAGTAAAAACTATCAACTGATAAGGAAATTAGTAAATATTTAACATCGCTAGAAGCAT

TGGTGAAATTTTAAGAAGGAAGGAACACATACCTTAAGACTCTTGTAACACTCTTTCAGG

TTATCATTATACAGCATATGATGAAAGATGCCTGACGATTGCCCTTGCTCAATCTCAGCC

TTGGCATTTTTAAGTCGCTTTAGGACCTGTTCCTCTGTCTCAGTTCCCCTGTTTAGCATA

TTTAAGCGTACTCAGAATTGGCAATGTGGAAGAGGAATTTGAGAATGAGAAAAATAAAAT

GGCATCTCAAACTACCTTGCACGAAGGCGCTTCTCAAGCTCCGCCATTGAGGGTGGACAT

ACAAAGATGAAAATAGCTTCCAGTGAACTAGCCCTCACGGATCTTGCACCTTGAACATCA

ATGTCAAGAATGCATCTCTACATTGAAAGCAAACAACATTACAGAATAAATAACCACAAG

AATCTCAGTAAAGAAAGCAGTGCTTGTGAGAGAGTGCCACTAACATAATTTAACAATATG

AGGGCGAAATCTAAAATACATAAAATATCCTCATAAGCTTTTCACAACTTTGACCATGAC

ATTAAGGATTATATGAATTAGCCTCTAATTGTATCCCATAAACCACACTACAATCTAGAA

AAGTTGTTCCAAATACAACTAATCATCATGCAGATGCTTTGATTCACCGCATGACAAATC

CATATAGCAAGTTTGACATGATAAAGAAAACGACCAAGCTCCAACAATAATCAAAATAGA

AACCACTGATCAAAGAAAGTTACCTTCCCATCATCTGCTACCACTTCTACTGCTTCAACA

CTAGTTCCATAGAGATTTCCATGCACAGAAGCAAACTCAAGGAACTTCCCATCTTCTATC

TCTTTCTCCATGGCACTTCGCTCAATGAAATGGTAATGGACCCCATCCTTCTCCATAGCT

CTAGGAGCACGGGTTGTGTGGCTCACAGAGAACCCAAACATGGAGGGATATTCTTTCATG

AGCATGGATATTAGTGTCCCCTTACCTACCCCAGAAGGACCACTAATAACAATTGGCTTC

TCAGCATAGCCCCTCACACCCTTACTCCAGGCAACCACGTCAGTGCCCAAAATTTTCTTC

TGCTGCCTCACATATTGGGTGTCCACCTAAAACAAAACAATTTTAGATTTCAGAATAACA

TAGTAATCATCTAAACATAATACGTGATGGACAGAATGGTGTTAACTGAACAATTCCACT

TTATTTAACAGCAACAAGGACAGGAGTTATTTTGTTCTCGCATCAATTGAATATAATTCT

ATTTCCAAACTTATTGTTTGAAACTCCTACTATCATTTGGTGCAGTCTCATTTTATCTTC

CATTGTCTGTCTCAAACTTAAACTATTACTAAATGAGACGACATTGTGACATCTTTTATT

CTGTTCAAATGAACTCTCCCTTATCATATCTGAATAGTTTCACAGGTACCCACCTCAAGG

AACCAAGCAAAATCGTCTGGGGTGGAACCATTCTTAGTAATCAATATTCGGTCTTCACTT

AAAAGCACTGCTGAAAGGCCCTTACAGGTTTTGGGTTTTGTTCCCAACACAGTAGGATAT

ATCCTGCAAATTTAAACCAGATATCAGTAACAAAATCAGACAAAAGCAAGTCAATACCAA

GAGTCATACACTTACCATTCACCAGTAGATTGGTCAAAAATCTGAACTCCAATTGACAAT

GGCGATTCCTCACAGGCTCCACCAATCACATACTGTCAAAAAATGCATTGGCAATATGAT

TGACCTGGTGGTAAATCACTGAAGAACAAACAGGGACATTGGATGACAAAATCATATTGA

TATGTTAAATATCATACAGTTCTGTTGCCCATGAGAATGGCTGTTTCACCGCCTTTGGAT

TTTATATCAAATCCATTTCCATATCCCTTCTGCAGCTCATCAACAATGAATGCTGGGGCT

TCTCCCTGATGACAACAACCAAAATCAAATCACATCAAAGGAACCATCTTGCTTCACATG

CAGACATCATTTCTAATTACATTCCTGATAAAAGTTAATTTATTAACCAATATTAAGTAT

AAACTAAGGATGAGGTGCTTACCATAGAGAAATCTGTTCCTGCAATTCTAGATTCCCCCT

GATCAGCAGAAATGGAGAGAGATGAGAGTAATTCAATATTTTCTGGATCTAAGAAATATA

TGAAAAGTTCAAGCCAGACGAGGCATAAAAGGCAGAGATTCCAACTAAACGTCTCTCTAT

TGCATTTGGAGAAAACAAAAACAACAAGAGTTTGAGCTGGAACCAGGTAAATACGATAAT

GAAGTTCAAAATCAAAACACAAATGCCCATGTCCTAATCCAAGATAGAAATACACATACA

GAAAGGGAAAAGCGATGCAACAAAGATACAATTGTTGGTTTTTTGTCCAAATCTCTTTAA

ACCCATAACAAAAACCAATTAGGAAGAGAGCAAAGAACATCAAAATGCAAACAAACATAA

ACCCACCTTAATATAGCTAAAACATATGGATCAAACCACTAACAAAGGGTTAAAACAACA

TTTGGAATAAAAGAAAAGGCTGAAAATATCAAACAGATTAAGACCCAGGTACCTTTGTGC

AAGCAGTTATTGCTCTATAACCAAAAATAAAAAGCAAGCTTATTTCTTGGCAGGGGATGT

ATTGTTCAGAGGGCCAAAGAAGGATTTAAAAGCCCACTTTGGGTGTATCTTTTTCAAGCT

GAAGTTTTGTGAAGGCTTGCAATCAAATCAACACGAACTTGGGATGGAAAAAATCACAAG

GGTGGTAGAATAAGTTGCAAGATATCCCATGAAGCATTCTCATATTTGTAGCATACAAAA

GAGGGAATCTTTAGTCCAATAGTCCTCCCTAGGAAGAAGTAGTGGTGAGAGAGAGAGAGA

GAGAGAGAGAGAGAGAGAGAGAGAGGCAGAGGCAGCAGAAGAAGAAGAGAGAGAAAGAGA

AAAGGGGTGGCCTTGTGGTCGCATTCAATTCAC
