# Supplementary material for: Ma Orthologous Genes in Prunus spp. Shed Light on a Noteworthy NBS-LRR Cluster Conferring Differential Resistance to Root-Knot Nematodes
Source: Front Plant Sci. 2018 Sep 11;9:1269. doi: 10.3389/fpls.2018.01269 (PMC6141779; doi:10.3389/fpls.2018.01269)
Supplement: Supplementary file 6 [file Table_6.DOCX]

**Table S6:** Transcription of the Ma orthologues in different Prunus species (A) and identification and localisation of kinase and Ma orthologues among diverse dicot genomes (B).

1. Transcription of the *Ma* orthologue in different *Prunus* species.

| **Bioproject** | **Archive** | **Experiment** | **Prunus species** | **Cultivar/clone** | **Reads mapping onto the Ma/ RMja/Prupe.7G65400 transcript** |
| --- | --- | --- | --- | --- | --- |
| PRJNA295439 | SRP063632 | SRX1187015 | Prunus salicina | Akihime | Yes |
| PRJNA295439 | SRP063632 | SRX1186990 | Prunus salicina | Formosa | Yes |
| PRJNA295439 | SRP063632 | SRX1186836 | Prunus persica | Mibaek | NO |
| PRJNA295439 | SRP063632 | SRX1186835 | Prunus persica | Jangtaek | Yes |
| PRJNA295439 | SRP063632 | SRX1187170 | Prunus cerasus | Schattenmorelle | Yes |
| PRJNA295439 | SRP063632 | SRX2393702 | Japanese plum | Yeonsik | Yes |
| PRJNA295439 | SRP063632 | SRX2393701 | plum | Gadam | Yes |
| PRJNA295439 | SRP063632 | SRX2393700 | European plum | - | Yes |
| PRJNA295439 | SRP063632 | SRX2393699 | plum | Hollywood | Yes |
| PRJNA295439 | SRP063632 | SRX2393698 | Prunus persica | Baekcheon | NO |
| PRJNA295439 | SRP063632 | SRX2393697 | Prunus persica | Cheonhong | Yes |
| PRJNA295439 | SRP063632 | SRX2393696 | Prunus persica | Cheonjung | Yes |
| PRJNA295439 | SRP063632 | SRX2393695 | Prunus persica | Janghowon | NO |
| PRJNA34817 | SRP013437 | SRX150227 | Prunus davidiana | Clone P1908 | Yes |
| PRJNA34817 | SRP013437 | SRX150233 | Prunus ferganensis | - | Yes |
| PRJNA34817 | SRP013437 | SRX150255 | Prunus kansuensis | Clone P1429 | PARTIAL |
| PRJNA34817 | SRP013437 | SRX150226 | Prunus persica | Bolero | Yes |
| PRJNA34817 | SRP013437 | SRX150228 | Prunus persica | Earligold | Yes |
| PRJNA34817 | SRP013437 | SRX173257 | Prunus persica tissues: embryos and cotyledons | - | NO |
| PRJNA34817 | SRP013437 | SRX150230 | Prunus persica | F1 Contender x Ambra | PARTIAL |
| PRJNA34817 | SRP013437 | SRX173255 | Prunus persica tissues: fruit | - | NO |
| PRJNA34817 | SRP013437 | SRX150234 | Prunus persica | GF305 | Yes |
| PRJNA34817 | SRP013437 | SRX173254 | Prunus persica tissues: leaf | - | Yes |
| PRJNA34817 | SRP013437 | SRX150254 | Prunus persica | Lovell (Clone PLov2-2N) | Yes |
| PRJNA34817 | SRP013437 | SRX150239 | Prunus persica | Oro A | Yes |
| PRJNA34817 | SRP013437 | SRX150243 | Prunus persica | Quetta | Yes |
| PRJNA34817 | SRP013437 | SRX173256 | Prunus persica tissues: root | - | Yes |
| PRJNA34817 | SRP013437 | SRX150247 | Prunus persica | Sahua Hong Pantao | Yes |
| PRJNA34817 | SRP013437 | SRX150251 | Prunus persica | Shenzhou Mitao | Yes |
| PRJNA34817 | SRP013437 | SRX150253 | Prunus persica | Yumyeong | Yes |
| PRJNA295439 | SRP063632 | SRX1187169 | Prunus mume | Wallyoung | Yes |

1. Identification and localisation of kinase Prupe.7G65700 and *Ma* orthologues among diverse dicot genomes.

| **Phylogeny** | | | | | **Species - Genome reference** | **Kinase Prupe.7G65700 orthologue and localisation** | **Ma/Rmja/Prupe.7G65400 orthologues** | **co-localisation in a same cluster** |
| --- | --- | --- | --- | --- | --- | --- | --- | --- |
| Dicots | *Rosids* | *Rosales* | *Rosaceae* | *Prunoideae* | Malus x domestica - GDDH13 v1.1 | Yes - MD12G1068200 - Chr. 12 | Yes - MD12G1068700 - Chr. 12 | YES |
|  |  |  |  |  | Pyrus communis - v1.0 | Yes - PCP021942.1 - scaffold01354 | Yes - PCP042053.1 - scaffold01354 | YES |
|  |  |  |  |  | Prunus persica Lovell - v2.0.a1 | Yes - Prupe.7G65700 - Chr. 7 | Yes - Prupe.7G65400 - Chr. 7 | YES |
|  |  |  |  |  | Prunus avium - v1.0.a1 | Yes - Pav_sc0002207.1_g540.1.mk - Chr. 7 | Yes - Pav_sc0002207.1_g580.1.mk - Chr. 7 | YES |
|  |  |  |  |  | Prunus mume - V1.0 | Yes - XM_008242650.2 - LG8 | Yes - XM_008242656 - LG8 | YES |
|  |  |  |  |  | Prunus dulcis - BAC 43O1 | Yes - Chr. 7 | Yes - Ma - Chr. 7 | YES |
|  |  |  |  |  | Prunus cerasifera - BAC 76H19 | Yes - Chr. 7 | Yes - RMjaGC2 - Chr. 7 | YES |
|  |  |  |  |  | Rosa chinensis - v1.0 | Yes - RC2G0340900 - Chr. 2 | Yes - RC1G0038200 - Chr. 1 | NO |
|  |  |  |  |  | Rubus occidentalis - v1.1 | Yes - Bras_T09080 - Chr. 6 | Yes - Bras_T07095 - Chr. 4 | NO |
|  |  |  |  |  | Fragaria iinumae - v1.0 (FII_r1.1) scaffolds | Yes - FII_iscf00049134.1 - FII_iscf00049134.1.g00003.1 | NO | NO |
|  |  |  |  |  | Fragaria x ananassa - v1.0 (FANhybrid_r1.2) scaffolds | Yes - FANhyb_rscf00000074.1.g00001.1 - FANhyb_rscf00000074.1 | NO | NO |
|  |  |  |  |  | Fragaria vesca - v4.0.a1 | Yes - FvH4_6g26380.1 - Chr. 6 | NO | NO |
|  |  |  |  | | Cannabis sativa - assembly ASM186575v1 | Yes - Cannabis sativa PK25108.1_1.CasaPuKu - Scaffold18395_2 | YES - PK17873.1_1.CasaPuKu - scaffold21832_1-scaffold2966_3 | NO |
|  |  |  |  |  | Ziziphus jujuba- assembly ZizJuj_1.1 | Yes - XM_016043495.1 - Chr. 12 | YES - NC_029683.1 - Chr5 | NO |
|  |  |  |  |  | Morus notabilis - (assembly ASM41409v2) | Yes - XM_024163356.1 - Scaffold1211 | YES - XM_010105907.2 - Scaffold1662 | NO |
|  |  | | | | Arabidopsis thaliana - TAIR10 | Yes - AT2G41890.1 - Chr 2 | NO | NO |
|  | | | | | Solanum lycopersicum - ITAG Release 3.20 | Yes - Solyc03g063650.1 - Chr3 | NO | NO |
