# Supplementary figures and images for: Ma Orthologous Genes in Prunus spp. Shed Light on a Noteworthy NBS-LRR Cluster Conferring Differential Resistance to Root-Knot Nematodes
Source: Front Plant Sci. 2018 Sep 11;9:1269. doi: 10.3389/fpls.2018.01269 (PMC6141779; doi:10.3389/fpls.2018.01269)

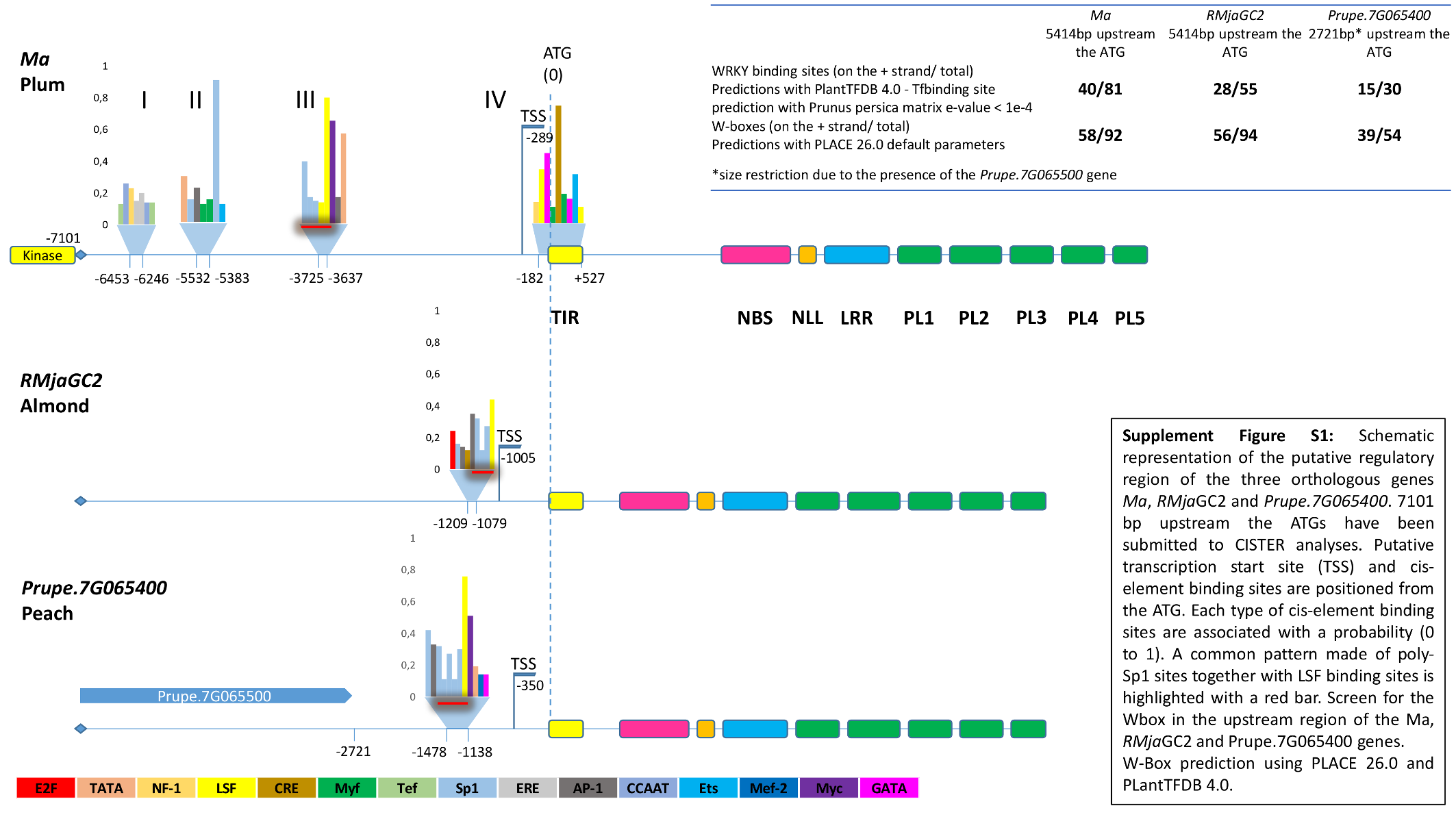

Supplement: Supplementary file 7 [file Image_1.TIF]

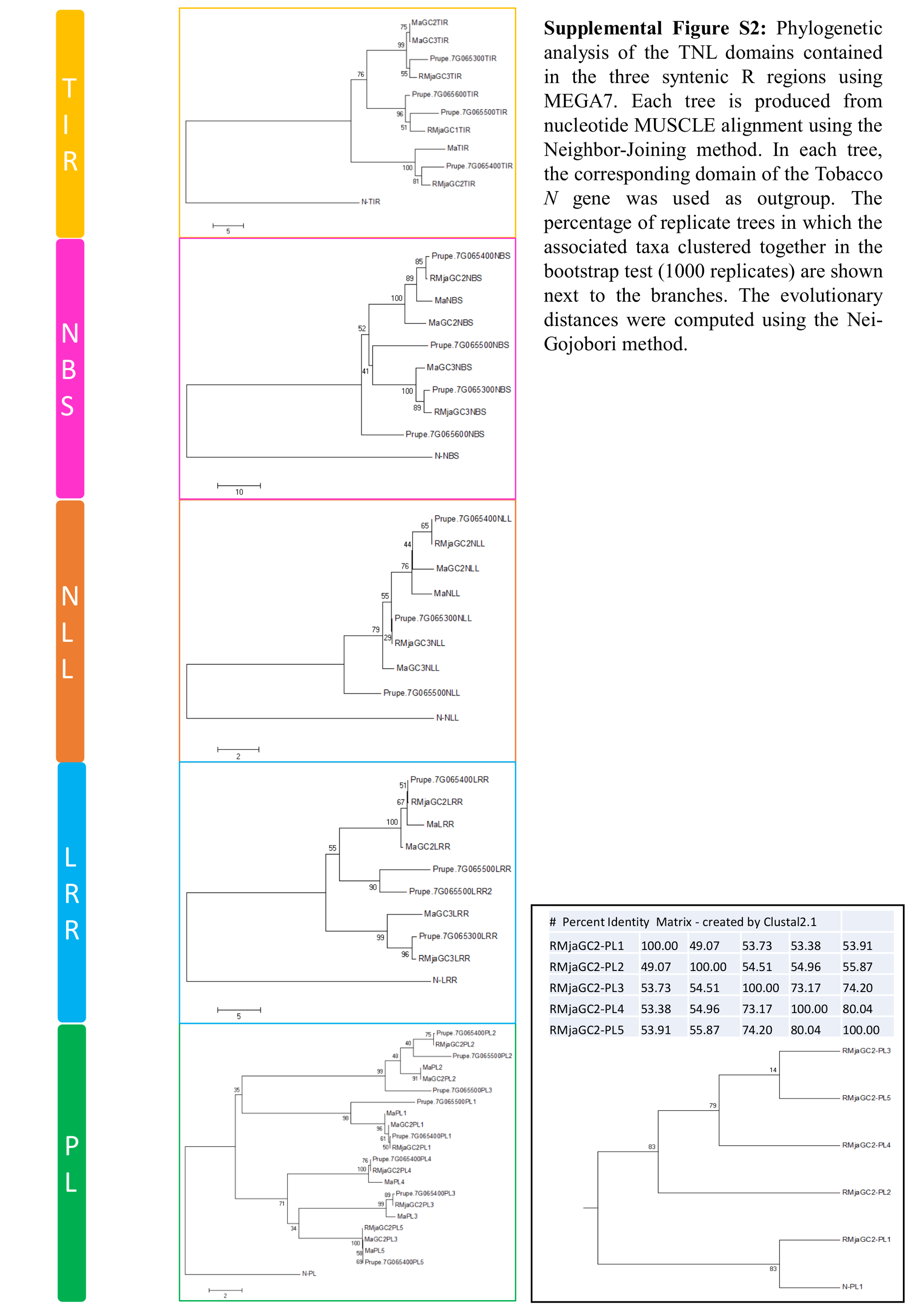

Supplement: Supplementary file 8 [file Image_2.TIF]
